# Agricultural investments and hunger in Africa modeling potential contributions to SDG2 – Zero Hunger

**DOI:** 10.1016/j.worlddev.2018.12.006

**Published:** 2019-04

**Authors:** Daniel Mason-D'Croz, Timothy B. Sulser, Keith Wiebe, Mark W. Rosegrant, Sarah K. Lowder, Alejandro Nin-Pratt, Dirk Willenbockel, Sherman Robinson, Tingju Zhu, Nicola Cenacchi, Shahnila Dunston, Richard D. Robertson

**Affiliations:** a International Food Policy Research Institute (IFPRI), Washington, D.C., USA; b Commonwealth Scientific and Industrial Research Organisation (CSIRO), 306 Carmody Road, St Lucia, QLD 4067, Australia; c Independent Agricultural Economist, Washington, D.C., USA; d Institute of Development Studies, University of Sussex, Brighton, UK

**Keywords:** Agriculture, Agricultural investments, Hunger, Africa, SDGs, Food security

## Abstract

We use IFPRI’s IMPACT framework of linked biophysical and structural economic models to examine developments in global agricultural production systems, climate change, and food security. Building on related work on how increased investment in agricultural research, resource management, and infrastructure can address the challenges of meeting future food demand, we explore the costs and implications of these investments for reducing hunger in Africa by 2030. This analysis is coupled with a new investment estimation model, based on the perpetual inventory methodology (PIM), which allows for a better assessment of the costs of achieving projected agricultural improvements. We find that climate change will continue to slow projected reductions in hunger in the coming decades—increasing the number of people at risk of hunger in 2030 by 16 million in Africa compared to a scenario without climate change. Investments to increase agricultural productivity can offset the adverse impacts of climate change and help reduce the share of people at risk of hunger in 2030 to five percent or less in Northern, Western, and Southern Africa, but the share is projected to remain at ten percent or more in Eastern and Central Africa. Investments in Africa to achieve these results are estimated to cost about 15 billion USD per year between 2015 and 2030, as part of a larger package of investments costing around 52 billion USD in developing countries.

## Highlights

• Increased agricultural investments alone will not achieve SDG2 in Africa; complementary non-ag investments will be needed.
• Climate change could lead to 16 million more people at risk of hunger compared to a scenario without climate change.
• Investment in agriculture can more than compensate for the negative effects of climate change.
• Enhanced agricultural R&D can reduce the prevalence of hunger by 55 million people in Africa.
• Multi-model ensemble used to more holistically assess cost and benefits of increased agricultural investments in Africa.

## 1 Introduction

The Second Sustainable Development Goal (SDG2) builds off the progress achieved under previous hunger eradication efforts (e.g. Millennium Development Goals; United Nations, 2015a) and presents the ambitious target of ending hunger globally by 2030 (United Nations, 2015b). Achieving this goal will require a multi-pronged effort that recognizes that the “last mile” to eradicate hunger will be the most difficult (Chandy, Kato, & Kharas, 2015). The next push will have to confront more binding environmental and resource constraints (Rockström et al., 2009, Tilman and Clark, 2014) even as food demand continues to increase due to a growing population (Kc & Lutz, 2017) and their growing demand for richer diets (Bodirsky et al., 2015, Pingali, 2007, Tilman and Clark, 2014). Climate change further complicates eradicating hunger by reducing agricultural yields, driving up food prices, reducing food availability, and increasing negative health outcomes (Springmann et al., 2016, Sulser et al., 2015, Wiebe et al., 2015).

Since the 1960s, increasing agricultural productivity has been critical for reducing poverty and hunger globally (Dercon and Gollin, 2014, McArthur, 2015, Pingali, 2012). However, Africa South of the Sahara (SSA) has benefited less than other regions from past investments and continues to have low agricultural productivity by global standards (GYGWPA, 2017). Closing agricultural productivity gaps alone will not be sufficient to ensure food supply can keep pace with growing demand (van Ittersum et al., 2016). Therefore, achieving SDG2 will require specific attention be given to agriculture in Africa and will have to take into account the particularities of the region and encompass a range of important regional crops beyond just the key staple cereals that fueled the Green Revolution (Pingali, 2012).

In this paper we present scenarios of agricultural productivity enhancements based on current and envisioned CGIAR efforts focused on the developing world. We then estimate the costs of achieving these projected improvements in the agriculture sector applying methodologies developed in the field of R&D investment and productivity gains (Alston et al., 2011, Esposti and Pierani, 2003, Evenson and Gollin, 2003, Nin-Pratt, 2016, Nin-Pratt et al., 2015). We then estimate the impacts of achieving these improvements on agricultural productivity, average incomes, commodity prices, food demand, and hunger using an augmented version of IFPRI’s IMPACT modeling system (Robinson et al., 2015), which was extended to include GLOBE (McDonald, Thierfelder, & Robinson, 2007), a global general equilibrium model, to capture the macroeconomic impacts of increased agricultural productivity (Rosegrant et al., 2017). Finally, we compare these results to several recent reports on reducing or eradicating hunger to highlight potential points of uncertainty with respect to the contributions of public investments in agriculture towards SDG2.

## 2 Materials and methods

### 2.1 Current levels of agricultural investment

We begin by providing rough approximations of current levels of investment in agriculture to provide context on agricultural investment scenarios explained in greater detail in following sections. The private sector is by far the largest investor in agriculture (FAO, 2012, Lowder et al., 2015) along with being the largest investor in measures to address climate change (Buchner, Trabacchi, Mazza, Abramskiehn, & Wang, 2015).

A dataset newly released by the United Nation’s Food and Agriculture Organization (FAO) shows that, in recent years, farmers in developing countries have invested approximately 153 billion USD annually on agricultural capital—similar to the $156 billion estimate of investments made by farmers in developed countries (Table 1).^1^ (Further details on private investments in Appendix S1). Government investments in agriculture have been smaller than private investments globally, with this difference being more pronounced in developed regions where government investments are about a third the level of private investments, compared to developing regions where it is about half. Public spending on agricultural research and development (R&D) (not shown in Table 1) is also an important component of government spending. Beintema, Stads, Fuglie, and Heisey (2012) show that in 2008, it accounted for almost $32 billion globally (about 25 percent of annual government investments in 2010–2012), with Africa and West Asia contributing about 11 percent of global investments.

**Table 1:** Average annual agricultural investments/spending in millions of constant 2005 US dollars, by source.

Region	Private investment (2010–2012)^1^	Government investment in agriculture (2010–2012)^2^	Development flows to agriculture (2013–2014)^3^	ODA for climate adaptation and mitigation through agriculture and forestry (2013–14)^4^	Agricultural spending by dedicated multilateral climate funds (2013–2014)^5^
Africa	10,027	3104	3163	1443	97
Northern Africa	1941	1140	59	15	0
South of the Sahara	8086	1964	3104	1428	97
Eastern Africa	2306	591	1267	652	42
Central Africa	388	138	29	7	3
Southern Africa	1553	479	45	23	3
Western Africa	3838	756	1114	369	47
Other developing	142,635	71,286	5057	2034	227
Developing Countries	152,662	74,390	8219	3477	324
Developed Countries	155,969	54,082	–	–	–
World	308,631	128,472	8219	3477	324

*Sources:* Author compiled following a method similar to that used for FAO (2012).

1 Private investment is gross fixed capital formation from FAO (2016a).

2 Government investment in agriculture is approximated as one half of government spending on agriculture. Government spending is from the SPEED database produced and made available by IFPRI (2015).

3 Development flows to agriculture from FAO (2016a).

4 Overseas Development Assistance (ODA) for climate adaptation and mitigation through agriculture and forestry from OECD (2016).

5 Agricultural spending by climate funds from ODI (2015).

As shown in Table 1, globally, development flows to agriculture (ODA to agriculture) are much smaller than private and governmental investments in agriculture, while dedicated funds for climate change adaptation and mitigation in agriculture are even smaller. However, ODA flows to agriculture are significant across Africa. In SSA there is a reliance of public budgets on ODA support with over three billion annually from ODA compared to less than two billion from government investments.

Although ODA for adaptation to and mitigation of climate change in the agricultural sector is quite small compared to other sources of finance, development assistance is the most easily tracked of any source of climate finance. Consequently, much discourse and analysis focuses on international public finance of climate change. Private sources of finance for climate change, whether domestic or international, remain largely hidden, which greatly limits our understanding of the overall financing of climate change adaptation and mitigation efforts (UNEP, 2016).

Work by Buchner et al. (2015) shows that if we consider financing (public, private, domestic, and international) to adapt to and mitigate climate change across sectors, we see that the private sector is, again, the largest source of funds. In 2014, about $391 billion was spent on the financing of adaptation to and mitigation of climate change; most was spent in the country of origin, with the bulk of investments made in East Asia and the Pacific, Western Europe, and the Americas. About $12 billion was invested in countries in SSA. Most public and private finance has funded mitigation efforts and the generation of renewable energy. The amount of private finance for climate change adaptation and mitigation allocated toward agriculture, forestry, and land use is unknown, but about six billion in ODA (of $148 billion of public finance) was allocated to these sectors, with about an even split between mitigation and adaptation. Public finance for climate adaptation and mitigation encompasses national, multilateral, and bilateral actors, with contributions from international organizations representing only a small fraction of the assistance (Buchner et al., 2015).

### 2.2 Modeling framework

To explore the role of increased agricultural productivity for food security in Africa, we use IFPRI’s IMPACT system of models (Robinson et al., 2015). IMPACT has been used extensively in projecting future agricultural production and demand and changes to food security globally and regionally (Delgado et al., 2001, Nelson et al., 2010, Rosegrant et al., 1999, Rosegrant et al., 2001, Sulser et al., 2011). It has been used to explore alternative agricultural productivity futures (Islam et al., 2016, Rosegrant et al., 2014), including the potential impacts of climate change on agriculture (Ignaciuk and Mason-D'Croz, 2014, Nelson et al., 2010, Sulser et al., 2015). IMPACT is linked to spatially explicit crop and hydrology models to integrate natural resource constraints on agricultural production (Robinson et al., 2015, Rosegrant et al., 2002). Additionally, IMPACT represents the global agriculture sector with a high level of geographic disaggregation and broad commodity coverage as compared to other similar models (Mason-D’Croz et al., 2016, Robinson et al., 2014), which makes it a good tool to analyze the potential of investing in African agriculture across a range of commodities.

The IMPACT system of models has, at its core, a highly disaggregated, global partial equilibrium multi-market model that simulates 62 agricultural commodity markets in 158 countries and regions. This multi-market model is directly linked to grid-based biophysical models (crop and hydrology models) that provide inputs on the impacts of changes in temperature and water availability at 0.5-degree resolution that are aggregated to summarize effects on agricultural production in 320 sub-national geographic units (Food Production Units or FPUs), which are the intersection of national boundaries with water basins. The DSSAT crop-modeling suite (Hoogenboom et al., 2012, Jones et al., 2003) provides estimated yields of crops at the grid-cell under varying management and climate scenarios. Crop land and crop allocation are determined at the FPU level using stylized land markets that simulate cropping choices based on changes to marginal revenue of crop production, such that total cropland and specific crop areas respond over time to changing crop productivity and prices. Water availability is modeled with the IMPACT suite of water models at the grid level and aggregated to FPUs, with water demand determined through crop/livestock life cycles, cropping patterns, and competition with non-agricultural sectors at FPU levels. IMPACT solves for equilibrium prices that clear world commodity markets by equating global supply and demand annually out to 2050. Food demand is simulated for all countries and regions based on changes in average income, population, and prices. Food demand then serves as an input to estimations of changes in food security, such as the population at risk of hunger (Fischer, Shah, Tubiello, & van Velhuizen, 2005) and malnourished children (Smith & Haddad, 2000). For more details on IMPACT see Appendix S2, which summarizes Robinson et al. (2015).

**Fig. 1:**
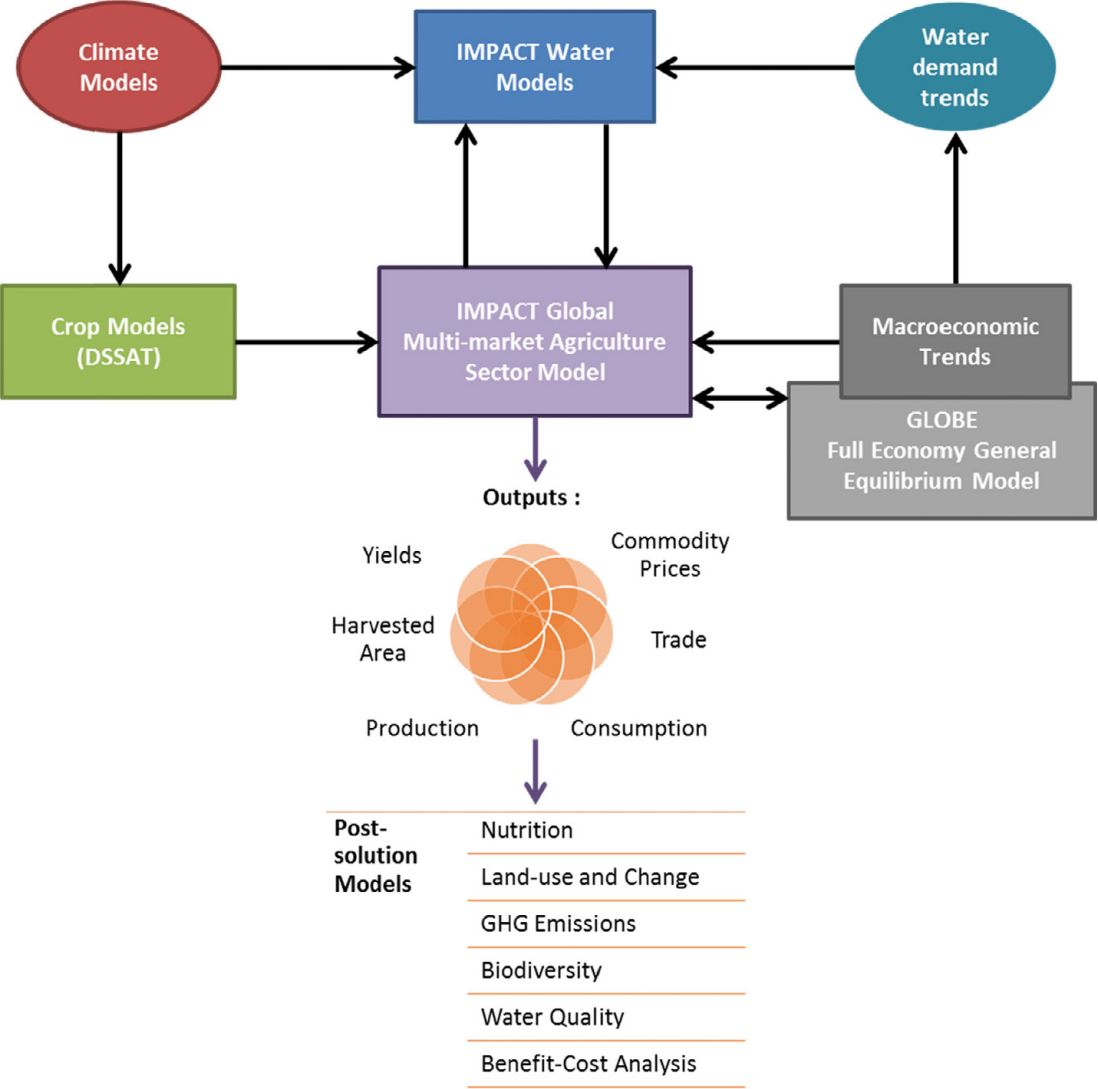
The Extended IMPACT modeling Framework. *Source:*Rosegrant et al. (2017).

As a partial-equilibrium model, IMPACT does not endogenously model the feedbacks between the agriculture sector and the broader economy. Investments in agriculture, particularly in developing countries where agriculture contributes a large share of total GDP, would be expected to have significant economic spillovers (Dercon and Gollin, 2014, Timmer, 2002). To better assess the potential of investments in agriculture to spur economic growth, the IMPACT framework was augmented to include an iterative link to GLOBE (Willenbockel et al., 2018), a global computable general equilibrium model (Fig. 1). The extended dynamic version of GLOBE employed here was initially calibrated to the GTAP 8.1 database (Narayanan, Aguiar, & McDougall, 2012) and represents global economy-wide production, demand, and trade for 24 sectors in 15 geographical regions. This version incorporates capital accumulation, population growth, labor force growth, and technical progress, and features a stylized representation of the technical substitution possibilities among different energy sources in production using a state-of the-art KLEM (Capital [**K**], **L**abor, **E**nergy, **M**aterials) technology specification. For more details on GLOBE see Appendix S3.

Changes in agricultural productivity from IMPACT are passed to GLOBE as factor productivity shifters that generate equivalent impacts on agricultural producer prices. Price changes lead to knock-on effects for non-agricultural sectors, ultimately leading to changes in real household incomes, which are then passed to IMPACT that incorporates these income dynamics into its food demand system. To bridge the varying levels of geographic aggregation between the two models we exploit the fact that the household real income deviations from the baseline are highly correlated with the initial shares of value added generated by food production. Using the fully disaggregated GTAP database we estimate the food value-added shares in GDP for 135 countries to downscale changes in real income from GLOBE to IMPACT. For more details on the coupling of IMPACT and GLOBE see supplementary Appendix S4, which summarizes Willenbockel et al. (2018).

### 2.3 Scenario assumptions

To explore how additional investment could affect agriculture and hunger in Africa, we ran three scenarios:

1. A “no climate change” scenario with current baseline model productivity assumptions (Sulser et al., 2015, Wiebe et al., 2015) and a constant 2005 climate called **NoCC**.
2. A scenario with baseline productivity assumptions and strong climate change impacts called **CC**.
3. A productivity enhancement scenario under climate change, called **COMP,** where productivity gains from additional investments in agriculture were added to the CC scenario.

#### 2.3.1 Baseline socioeconomic assumptions

The baseline socioeconomic assumptions start with the “middle of the road” scenario (SSP2) of the Shared Socioeconomic Pathways (SSP), a set of global scenarios developed for the IPCC’s Fifth Assessment Report (Moss et al., 2010, O’Neill et al., 2017, O'Neill et al., 2014). The SSP2 scenario corresponds to the medium variant of IIASA-VID-Oxford population projections, where global population reaches 8.3 billion by 2030 with an economy of US$143 trillion (Dellink et al., 2017, Jiang and O’Neill, 2017, Kc and Lutz, 2017). SSP2 is a useful contextual scenario both at the global and regional scale and has served as a point of reference for several regional scenario exercises, which downscaled the SSPs in East (Vervoort et al., 2014) and West Africa (Palazzo et al., 2017).

Under SSP2, expected changes in population and economic growth vary substantially by region (Table 2). Population growth is concentrated in the developing world, where population grows at more than one percent per year adding, by 2030, almost 1.4 billion people globally (0.5 billion in Africa), compared to about 0.1 billion in developed countries. Economic growth is also fastest in developing countries, with an average annual growth rate of five percent compared to two percent in developed countries. Africa grows at the developing country average (5.2 percent), with Western and Eastern Africa growing somewhat faster at 6.5 percent per year. Central, Northern, and Southern Africa grow below the regional average at 5.0, 4.7 and 3.9 percent respectively. Robust economic growth across Africa pushes up average incomes, with the regional average annual income nearly doubling to $5000 by 2030. Nevertheless, by 2030, average annual incomes in Africa would still be below the developing country average, with only Southern Africa exceeding the developing country average.

**Table 2:** Regional changes under SSP2 for key socioeconomic indicators in 2010, 2020, and 2030.

Region	Population (million)			GDP (Trillion USD)			Average annual income (000 USD/person)		
	2010	2020	2030	2010	2020	2030	2010	2020	2030
Africa	1032	1279	1538	2.8	4.5	7.7	2.7	3.6	5.0
Northern Africa	223	260	293	1.2	1.8	2.9	5.2	6.9	9.9
South of the Sahara	863	1086	1326	1.7	2.9	5.0	2.0	2.7	3.8
Western Africa	304	386	479	0.5	1.0	1.9	1.7	2.5	3.9
Eastern Africa	321	407	498	0.3	0.6	1.1	1.0	1.5	2.3
Central Africa	127	162	200	0.2	0.4	0.6	1.9	2.4	3.1
Southern Africa	58	63	68	0.5	0.8	1.1	9.0	12.3	16.6
Other Developing Countries	4508	4903	5205	26.6	48.4	76.1	5.9	9.9	14.6

Developing Countries	5778	6460	7058	31.4	56.0	88.2	5.4	8.7	12.5
Developed Countries	1102	1167	1222	36.1	45.3	55.0	32.8	38.8	45.0

World	6879	7626	8280	67.6	101.3	143.1	9.8	13.3	17.3

*Source:* SSP population (Jiang and O’Neill, 2017, Kc and Lutz, 2017) and GDP (Dellink et al., 2017) downloaded from SSP database (IIASA, 2013). USD are reported in constant 2005 terms.

#### 2.3.2 Baseline climate change assumptions

The second scenario (**CC**) adds climate change to the baseline socioeconomic assumptions. Work done in the Agricultural Model Intercomparison and Improvement Project (AgMIP) has explored various dimensions of uncertainty around climate change impacts on agriculture. First, there is uncertainty on future GHG concentration levels, which will depend on economic growth and technological development. There is also uncertainty to the effects of increasing GHG levels in the atmosphere on changing temperatures and precipitation patterns and the ultimate impact of these changes on crop yields (Rosenzweig et al., 2014, Ruane et al., 2018, von Lampe et al., 2014). We use the **CC** scenario not as a projection of climate change impacts, which ideally would include multiple climate models to give a range of potential outcomes, but more as a benchmark to provide context of the potential benefits of increased agricultural investment. As such, we designed the **CC** scenario to be a more “extreme” climate scenario to explore diverse alternative climates similar to other modeling exercises (Mason-D’Croz et al., 2016, Nelson et al., 2010, Nelson et al., 2014).

**Fig. 2:**
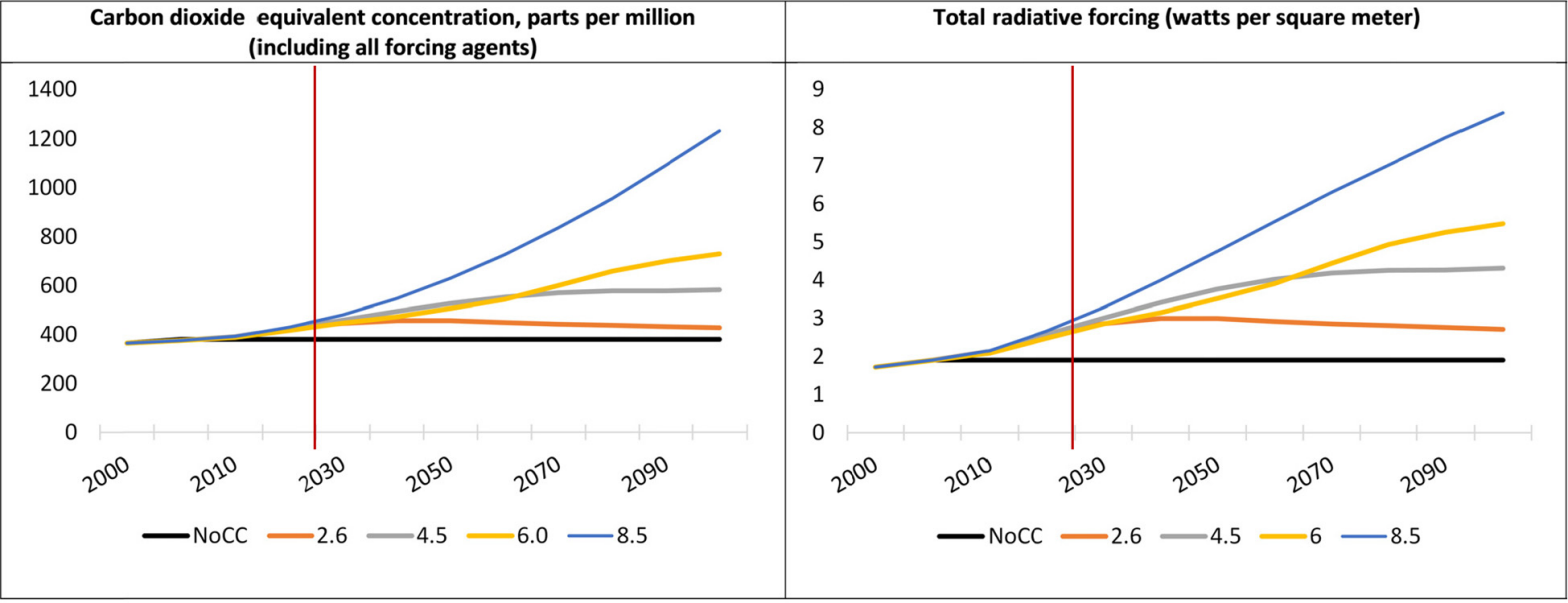
CO_2_ equivalent concentration and radiative forcing for IPCC's four RCPs and a NoCC (constant 2005 climate) scenario. *Source:* Based on Robinson et al. (2015); Data downloaded from the RCP Database version 2.0.5 (IIASA, 2015. RCP 2.6: van Vuuren et al., 2006, van Vuuren et al., 2007. RCP 4.5: Clarke et al., 2007, Smith and Wigley, 2006, Wise et al., 2009. RCP 6.0: Fujino et al., 2006, Hijioka et al., 2008. RCP 8.5: Riahi and Nakicenovic (2007). *Note:* NoCC—no climate change or constant 2005 climate; Red line represents the 2030 endpoint for achieving SDGs. (For interpretation of the references to colour in this figure legend, the reader is referred to the web version of this article.)

The IPCC’s Fifth Assessment report (IPCC, 2013) provides a range of future climate scenarios called Representative Concentration Pathways (RCPs). Through 2030, there are limited differences in atmospheric concentration of GHGs across the four RCPs ranging from concentration levels between 445 ppm and 480 ppm in 2030 compared to approximately 375 ppm in 2005. Radiative forcing by 2030 ranges from 2.9 W/m^2^ to 3.3 W/m^2^ compared to 1.9 W/m^2^ in 2005. The RCPs begin to diverge more significantly by mid- and end-of century (Fig. 2). In the **CC** scenario, we have chosen to apply RCP 8.5, the most severe of the RCPs, and which a rising radiative forcing pathway leading to 8.5 W/m^2^ (∼1370 ppm CO_2_ eq) by 2100 (van Vuuren et al., 2007, van Vuuren et al., 2006).

**Fig. 3:**
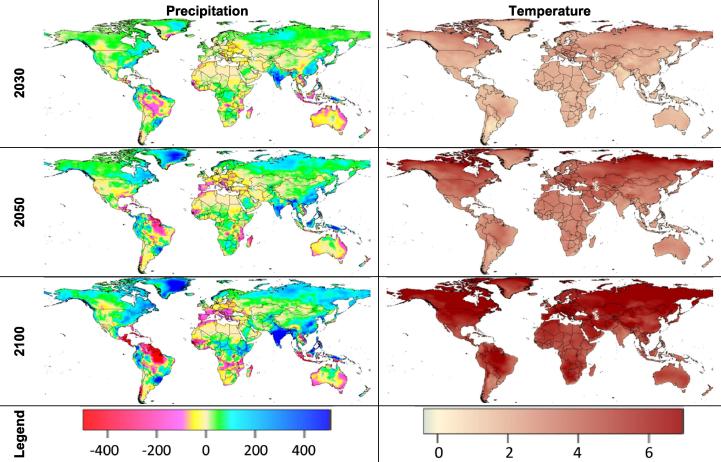
Change from 2000 in annual precipitation (mm) and maximum temperature (°C) for HGEM using RCP8.5 by 2030, 2050, and 2100. *Source:* Climate data comes from CMIP and ISI MIP (Rosenzweig et al., 2014, Taylor et al., 2012, Warszawski et al., 2014) and are downscaled for use in the crop models.

Climate models vary in how they simulate the effects of a given GHG concentration pathway on temperature and precipitation. In this report, we focus our analysis on the climate realization from the Hadley Center’s Global Environment Model (**HGEM**; Jones et al., 2011). We selected HGEM because this climate model projects the most severe global consequences to agriculture from climate change with respect to changes in temperature and precipitation under RCP 8.5 (Fig. 3) and thus represents the most negative global climate scenario of those scenarios that were available from the freely available dataset of the ISI-MIP fast track initiative (Warszawski et al., 2014). At the other extreme, the “No-Climate-Change” (**NoCC**) case presents a future climate with no additional climate change.^2^

In the **NoCC** scenario, aggregate crop productivity in Africa is assumed to increase by 38 percent between 2010 and 2030 (Sulser et al., 2015). The changing temperatures and precipitation represented in Fig. 3 would lead to a negative biophysical shock that would decrease 2030 yields in Africa by about seven percent, excluding possible economic adaptations. The negative effects of climate change in Africa are higher than for the average across developing countries or the global average (five and six percent respectively).

#### 2.3.3 Comprehensive agricultural investment scenario assumptions

The third scenario presented is a comprehensive investment scenario (**COMP**) for agriculture and the rural sector, which combines investments in agricultural research, resource management, and infrastructure in developing countries. This scenario is drawn from related work (Rosegrant et al., 2017) assessing the potential impact of various investment levels across the CGIAR on agricultural development and sustainability.

The **COMP** scenario was selected for this analysis for three main reasons. First, it responds to the question of what outcomes could be achieved through increased public funding to agricultural R&D. The CGIAR has a long history of successfully converting research into agricultural and economic development (Evenson and Rosegrant, 2003, Renkow and Byerlee, 2010) and this scenario builds on this network having been developed jointly with experts across the CGIAR system and embodies an optimistic, but plausible, scenario of the potential for improvements to the agricultural sector in a future with higher investments. Second, the investments represented in this scenario are well suited to responding to the problem of underinvestment in public goods technologies, in which the CGIAR specializes. Tassey (2005) identified the four primary causes of underinvestment as: 1) technical complexity; 2) long time horizons between investments and returns; 3) challenges of scale and scope of research; and 4) leakages. The CGIAR specializes in the provision of global goods technologies and the heart of its mission is to work on the types of research most likely to see underinvestment. It also plays a critical role in developing and helping diffuse technologies by building regional research capacity that, in turn, should increase the region’s ability to absorb new technologies (Aghion & Jaravel, 2015). Third, with limited knowledge of what the future will bring, we believed it was better to look at a scenario that would help prepare for the future (Nordmann, 2014, Vervoort and Gupta, 2018), instead of trying to predict the “best path forward.” Given the broad future uncertainty, we also believed it reasonable to follow portfolio theory and consider a broad and diversified mix of investments (Markowitz, 1952, Markowitz, 1991). The **COMP** scenario is just such a mix, encompassing investments across the agriculture sector.

All the interventions specified in **COMP** are assumed to begin after 2015, with the scenario projection horizon going to 2050. The 2030 results presented in the following sections show the outcomes of these interventions at the midpoint of the overall scenario. While we focus on investments in Africa, the analysis includes investments in all developing regions, recognizing the importance of spillovers in technology and market effects to eliminating hunger in any one region and the difficulty and high cost of trying to do so in isolation.

The **COMP** scenario considers the role of improving agricultural productivity throughout the developing world, focusing on the potential gains in reducing yield gaps from increased CGIAR investment in agricultural R&D. The target yield improvements were quantified through consultations with CGIAR scientists on plausible yield gains from increases in research budgets. These yield gains were quantified for the developing world at the country level, differentiated across irrigated and rainfed systems, and incorporated knowledge on varying regional production levels and local research and extension capacity.

**Fig. 4:**
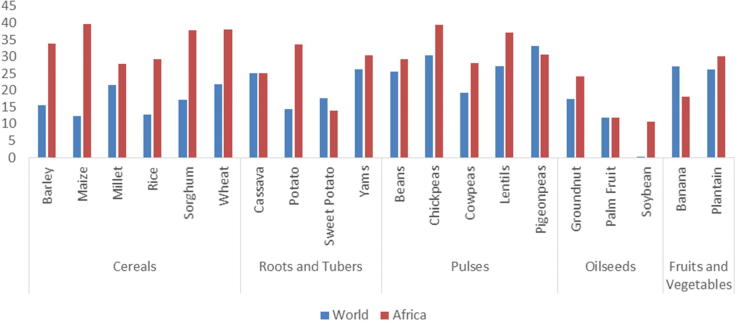
Target crop productivity improvements in Africa and globally by 2030 (percent change from the baseline climate change scenario, HGEM). *Note:* These figures summarize productivity improvement assumptions in the COMP scenario. All productivity targets were specified at the country level and disaggregated by irrigated and rainfed management. The World value includes Africa.

Fig. 4 summarizes the target improvements in crop productivity globally and specifically in Africa. Agricultural productivity in Africa lags significantly behind the rest of the world and, therefore, the productivity gains possible in this region are significant in percentage terms. Africa in general is projected to be catching up with the rest of the world in the coming decades and productivity gains are generally projected to increase at a faster rate than the global average. Assuming some level of convergence may be optimistic, given that African cereal yields have not grown at a rate significantly faster than the global average since 1990 (FAO, 2016a), however it is consistent broadly with the SSP 2 narrative, which projects economic convergence (Table 2).

**COMP** also considers the critical role of water in sustainable intensification of the global food system. Expanding the area equipped for irrigation is a powerful option in terms of both increasing crop yields as well as reducing the risk of production volatility due to changing weather. At the same time, however, water can be a scarce resource and the expansion of irrigation in a sustainable fashion requires investments in water use efficiency. Water use efficiency investments in all targeted regions increases efficiency by 15 percentage points. Table 3 summarizes the targeted increases in irrigated area, water use efficiency, and soil water availability.

**Table 3:** Scenarios target increases by 2030 in irrigated area and soil water availability, by region (percent increase relative to baseline).

Region	Irrigated area	Water use efficiency	Soil water availability
Africa South of the Sahara	30	30	15
North Africa and West Asia	5	25	5
Africa and West Asia	13	27	14
Other Developing Countries	5	25	6
All Developing Countries	6	25	8
World	5	20	6

High transportation and marketing costs reduce the potential revenues that producers can make while increasing the prices that consumers face. The **COMP** scenario also includes investments in improvements to transportation (roads, rail, and ports) and energy infrastructure (expansion and improvement of electrical network), which are major constraints to transportation and storage of agricultural production. These scenario assumptions were represented as declines in the price wedges between the farm-gate price and the prices consumers face.

### 2.4 Estimating scenario investment costs

Potential investment costs are assessed quantitatively for the **COMP** and baseline scenarios (**NoCC** and **CC**) with the understanding that the timing of investments is inherently different according to the targeted driver of agricultural production (e.g. crop yields versus irrigation expansion). Infrastructure investments in irrigation, as well as investments in improving marketing efficiency, require more up-front spending to expand and improve irrigation and transportation networks, whereas investments to increase agricultural yields through increased TFP require a longer-term stream of research and development activities (see Appendices S5–S7 for more details on investment calculations).

Infrastructure investments are estimated using unit costs of construction and maintenance. However, given the challenges of capturing the “lumpy” development of new technology, where progress can be observed in fits and bursts, with advances in basic science pushing continued gains in applied science for a period of time (Tassey, 2005), we decided to structurally model the process of converting R&D investments into agricultural productivity gains. To do this, we follow a long literature of translating R&D investments into changes on sector productivity (Alston and Pardey, 2001, Alston, 2018, Alston et al., 2009, Ball et al., 2013, Esposti and Pierani, 2003, Fuglie, 2012, Fuglie, 2017, Griliches, 1958, Griliches, 1979, Griliches, 1994, Huffman, 2018) and chose to employ the perpetual inventory methodology (PIM), considered the “the workhorse of R&D stock estimation” techniques (Hall, Mairesse, & Mohnen, 2010).

The PIM includes not only the building of the stock of knowledge, but also acknowledges the process of “creative destruction” as new methods of production make the old obsolete (Schumpeter, 1942). We follow Esposti and Pierani (2003), who link the parameterization of the PIM (in particular the decay rate and R&D elasticities) with the type of research being modeled (basic, applied, and developmental research). Additionally, we recognize different regional and institutional returns to R&D investment (Evenson & Gollin, 2003) and disaggregate agricultural R&D by region and source of public investment (CGIAR or National Agricultural Research Systems (NARS)). This allowed us to represent the variation of regional and institutional capacity, as well as in what types of research each institution specializes (i.e. CGIAR tends to work more on basic research). We further apply lessons from Alston et al. (2011) to explicitly incorporate spillovers into the process of the building of stock of knowledge.

#### 2.4.1 Baseline investments

The additional public investments included in the baseline for Africa and West Asia^3^ are projected to be more than $9 billion per year, with about $7.5 billion in direct government investments in agriculture (excluding infrastructure and the CGIAR Col 2–5 in Table 4). This will require government investments in agriculture to more than double currently estimated levels (Table 1) by 2030, a significant increase, but one that is plausible given the low levels of investments compared to other developing regions and the economic growth projected for Africa under SSP2 (Table 2). Globally, the additional investments assumed in the baseline are more than $86 billion per year, with about $34 billion coming from direct investments in agriculture, which can be categorized primarily as either investments in R&D or in irrigation and water resource use.

**Table 4:** Projected average annual baseline costs between 2010 and 2030 (billion 2005 USD).

Region	Direct investments in agriculture					Infrastructure (6)	Total (7)
	Agricultural R&D		Irrigated area (3)	Water use (4)	Soil management (5)		
	CGIAR (1)	NARS (2)					
Africa South of the Sahara	0.68	0.85	2.98	0.13	0.88	0.17	5.7
North Africa and West Asia	0.06	1.12	0.81	0.07	0.64	0.90	3.6
Africa and West Asia	0.74	1.97	3.79	0.20	1.52	1.07	9.3
Other Developing Countries	0.41	3.38	3.77	2.02	2.10	24.50	36.2
All Developing Countries	1.16	5.35	7.56	2.22	3.62	25.57	45.5
Developed Countries	.	13.26	0.56	0.17	.	26.92	40.9
World	1.16	18.61	8.12	2.39	3.62	52.49	86.4

*Note:* CGIAR investments focus on developing countries, and soil management investment data are available only in developing countries.

In IMPACT, improvements in agricultural productivity are embedded in growth rates, which represent historical trends on agricultural yield growth and biological yield potential (Robinson et al., 2015). To assess the future costs of the projected yields, we have calculated the required investments in agricultural R&D to achieve given changes in agricultural productivity using PIM. The CGIAR contributed about three percent of total public R&D funding in developing regions; however, in SSA it contributes about twelve percent (ASTI, 2016, Beintema et al., 2012). The projected investment costs for Africa are $2.7 billion per year, with CGIAR investments projected to follow historical trends and keep pace with GDP growth from 2010 to 2050 (4.1 percent compared to 4.4 percent for GDP). Investments grow faster than GDP to 2030 (6.3 vs. 4.7 percent) to account for research lags between initial investments and productivity growth. NARS investments are also projected to increase across the region from $1.6 to $2.4 billion by 2030, an increase of over 50 percent.

In 2010, Africa and West Asia accounted for about 10 percent of the world’s irrigated area, about 32 million hectares. Total irrigated area in IMPACT is projected to increase in the region by approximately six million hectares by 2030, with this increase split evenly between SSA, and North Africa and West Asia. In SSA this is an increase of over 33 percent, whereas for North Africa and West Asia, the increase is about 13 percent over 2010 levels. In addition to expanding irrigated areas throughout the region, the baseline scenario considers that new water resource management technologies will be implemented over time. There is significant variation globally on the efficiency of water management systems due to irrigation technologies (e.g. drip vs. furrow; Rosegrant et al., 2002, Ignaciuk et al., 2015) and crop management (e.g. no-till, Rosegrant et al., 2014). The baseline scenario assumes improving regional water use efficiency based on historical trends, which leads to a 4 percent decline in water use intensity per hectare (m^3^/ha). Using cost data on water projects from Inocencio et al. (2007) and FAO’s AquaStat Database (FAO, 2016b), the average annual cost of investments to achieve this level of improvement to the irrigation system amounts to about $4 billion per year from 2010 to 2030. The majority of the investments is dedicated to irrigation expansion, particularly in SSA. Improvements in soil management were estimated to be more than $1.5 billion per year with SSA requiring about 58 percent of costs for Africa and West Asia.

The economic growth projected as a part of the baseline SSP2 assumptions is significant across the developing world, particularly for SSA. This growth will require investments in expanding and maintaining transportation and energy infrastructure, which are critical for not only the agriculture sector, but the economy as a whole. Following Rosegrant, Magalhaes, Valmonte-Santos, and Mason-D’Croz (2018), we estimate global infrastructure investments to be about $52.5 billion per year. In Africa and West Asia, the investment levels will be much smaller than in other regions at about $1.1 billion per year, which may seem counterintuitive due to lower levels of infrastructure in the region. However, a significant share of infrastructure investment is focused on maintenance and preventing deterioration. The low levels and quality of existing infrastructure throughout Africa and West Asia lead to lower investment costs in the region as investments are focused on new construction instead of maintaining older infrastructure.

#### 2.4.2 Comprehensive agricultural sector investments to help end hunger in Africa

The **COMP** investment portfolio represents a broad array of additional investments across different parts of the agricultural system. If these investments were to occur simultaneously, we would expect the total additional cost for all developing countries to be about $52 billion per year from 2015 to 2030 (Table 5), an amount comparable to assumed baseline infrastructure investments. The additional direct investments in agriculture (excluding infrastructure) account for about $26 billion per year, of which about $8 billion per year would be in Africa and West Asia. The total investment cost for Africa and West Asia, including infrastructure, is almost $15 billion per year (29 percent of total investment across all developing countries).

**Table 5:** Total additional cost of Comprehensive Investment Scenario (billion 2005 USD).

Region	Direct investment in agriculture				Infrastructure (5)	Total (6)
	Agricultural R&D (1)	Irrigation expansion (2)	Water use efficiency (3)	Soil & water management (4)		
Africa South of the Sahara	0.67	2.76	0.41	1.76	4.52	10.1
North Africa and West Asia	0.01	0.83	0.61	1.28	2.06	4.8
Africa and West Asia	0.67	3.59	1.02	3.04	6.57	14.9
Other Developing Countries	0.07	4.34	9.25	4.19	19.36	37.2
All Developing Countries	0.74	7.93	10.27	7.23	25.94	52.1

*Note:* Additional investments are specified only in developing countries.

**Fig. 5:**
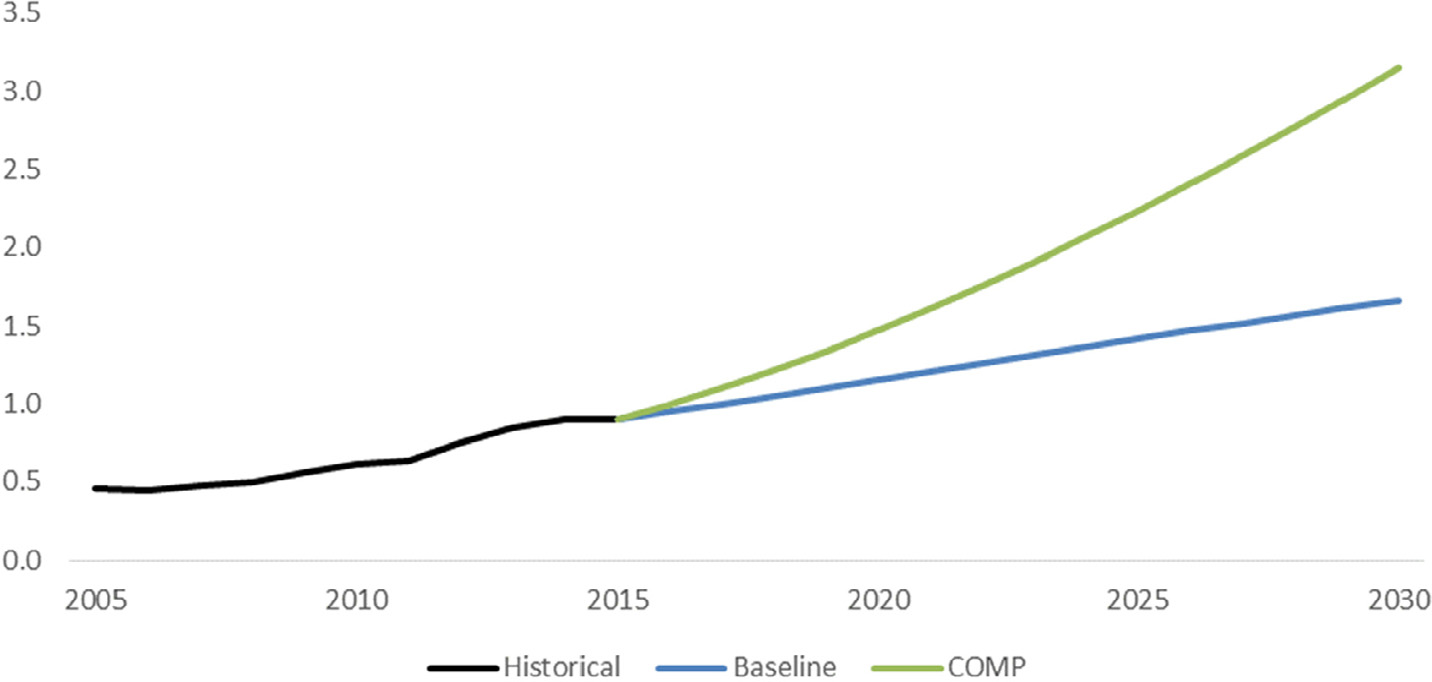
CGIAR investment streams in the COMP and Baseline scenarios (billion 2005 USD).

To achieve the additional productivity enhancements projected in the COMP scenario illustrated in Fig. 4, total CGIAR investments in agricultural R&D would start to diverge from the baseline in 2015, increasing to more than $3.1 billion by 2030, almost double the investment level in 2030 in the baseline scenario ($1.7 billion) (Fig. 5). This amounts to an average annual investment of $0.74 billion per year in additional investments in CGIAR research. These investment increases will not be uniform across regions, with nearly 90 percent of additional investments ($0.67 billion per year) needed in SSA.

Plausible investments in agricultural research to boost yields by 2030 will not be sufficient to eliminate hunger in Africa. Additional investments to improve the productive capacity of agriculture and boost the sector’s efficiency will be required. These investments are also critical towards improving the sustainability of the agriculture sector ensuring that gains achieved by 2030 can be maintained moving further into the 21st century. The additional agricultural investments considered in this analysis include investments to improving the capacity of water resource management (more efficient irrigation technologies, implementation of soil water management technologies), as well as further expansion of irrigation beyond the assumed expansion in the baseline.

The additional investments needed to expand irrigation, and improve water resource management throughout the developing world are significant, more than $25 billion per year (columns 2–4 in Table 5), nearly doubling baseline investments for irrigation expansion (Table 4). About $7.7 billion, or about 31 percent, of this additional investment will be in Africa and West Asia. Most of this investment in Africa and West Asia will be in irrigation expansion ($3.6 billion, 47 percent) and soil management ($3 billion, 40 percent). Within this region almost two thirds of the investments are concentrated in SSA.

Agricultural markets throughout the developing world suffer from market inefficiencies due to poor infrastructure. To reduce these inefficiencies, and thereby reduce transportation and marketing costs it is necessary to invest in building new infrastructure as well as improving and maintaining current infrastructure. These investments enhance productivity along the value chain, increase the speed of moving commodities to markets while also improving storage capacity, all of which improve market efficiency by better matching supply and demand over time. In this scenario, infrastructure investments focus on expanding and improving energy and transportation infrastructure. The cost of infrastructure investments is based on Rosegrant et al. (2018), where infrastructure investments across the developing world in road network expansion and improvement, rail expansion, and increased electrification were estimated to cost almost $26 billion per year between 2015 and 2030. Investments in Africa and West Asia account for about $6.6 billion (25 percent) of the total infrastructure investments with the majority dedicated to the improvement of road connections.

## 3 Results

The developing world is projected to experience important improvements in overall well-being and reduced hunger in the **NoCC** scenario. The investment scenarios described above, combined with other drivers, will play a significant role in this positive trajectory. Economic growth is particularly important with average incomes in the developing world more than doubling between 2010 and 2030 in the baseline scenario. Africa experiences fairly optimistic growth in per capita incomes of more than three percent per year during this period. Although, we should note that changes in average incomes can still mask significant diversity of income and poverty levels at the household and individual level. Climate change (**CC**) is projected to slow global income growth by about 1 percent over this period and have stronger effects in the developing world, especially in Africa and South Asia.

### 3.1 Income

Coupling the GLOBE and IMPACT models allows endogenous determination of the aggregate income effects of changes in agricultural productivity. The **COMP** scenario, combining high investment in agricultural R&D, irrigation expansion, improvements in water use efficiency and soil water management, and reduced marketing margins, sees significant increases in income and manages to more than offset the negative effects of climate change on incomes by 2030. Globally, average incomes increase by almost $500 per person (about three percent) in 2030 compared to the **CC** scenario without added investments. Developing countries generally benefit more, with Africa seeing average incomes increasing by more than five percent (over $200 per person). The regional average masks variation within Africa, with larger growth observed in West Africa ($300 per person) compared to Central Africa ($60 per person) (Table 6).

**Table 6:** Comparing average income (per capita GDP in thousand USD) in 2010 and 2030 by region and scenario.

Region	2010	2030		
		NoCC	CC	COMP
Africa	2.7	5.0	4.9	5.1
Northern Africa	5.2	9.9	9.8	10.2
South of Sahara	1.9	3.8	3.8	4.0
Western Africa	1.7	3.9	3.8	4.1
Eastern Africa	1.0	2.3	2.3	2.3
Central Africa	1.9	3.1	3.1	3.2
Southern Africa	9.0	16.6	16.5	17.3

Developing Countries	5.4	12.5	12.4	12.9
World	9.8	17.3	17.2	17.6

### 3.2 Agricultural productivity

**Fig. 6:**
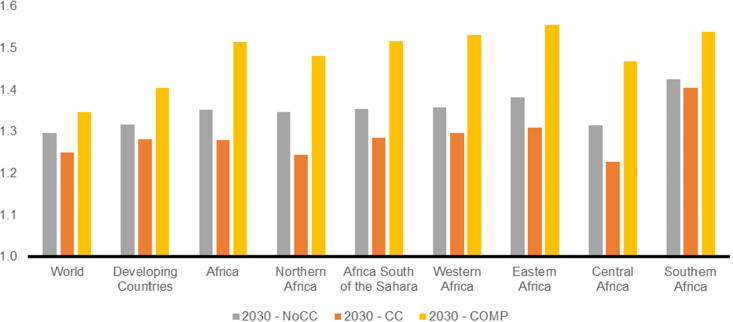
Average Aggregate Crop Yields in 2030 (indexed, 2010 = 1.0) by region and scenario.

Agricultural productivity is projected to increase by about 32 percent in the developing world between 2010 and 2030 in the absence of climate change (**NoCC**). Climate change reduces this potential productivity growth by four percentage points (with variation by crop and region) as yields respond to changes in temperature and precipitation and, in turn, to changing prices. The additional investments in agriculture in the **COMP** scenario have significant effects on yields, with gains more than offsetting the negative effects of climate change and increasing global crop productivity in 2030 by 40 percent compared to 2010. This is 12 and 8 percentage points above the **CC** and **NoCC** scenarios, respectively. Africa gains significantly in this scenario with crop productivity increasing by 51 percent over 2010 levels, from 47 to 56 percent, depending on the region (Fig. 6).

### 3.3 Commodity prices

**Fig. 7:**
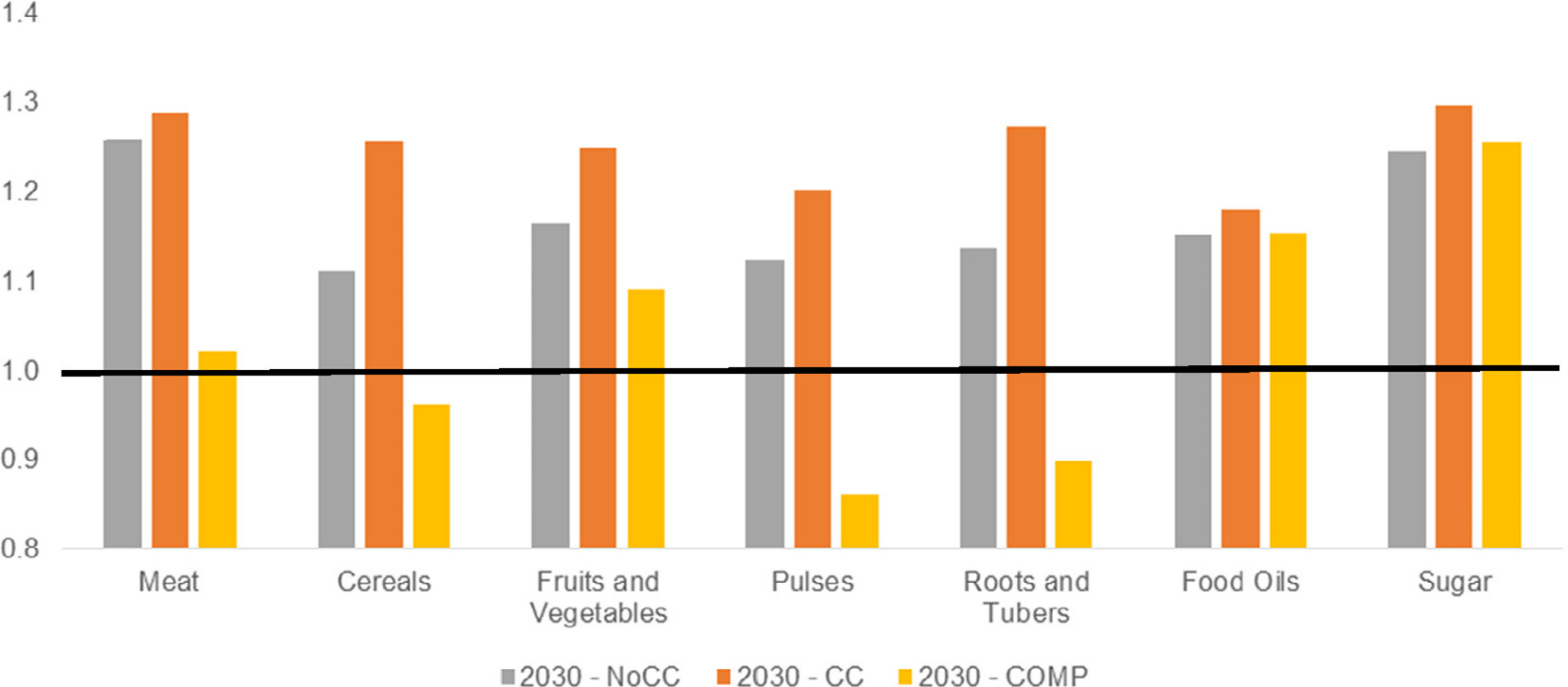
World Prices in 2030 (indexed, 2010 = 1.0) by commodity group and scenario. *Note:* The black line represents 2010 price levels.

The interplay of food prices and income ultimately determines the ability of consumers to purchase the quantities of food they demand. Growing population, increasing incomes, and climate change are all factors that will likely lead to higher food prices. By 2030, prices are projected to increase between 10 and 30 percent from 2010 levels under a baseline without climate change (Fig. 7). Climate change, on average, increases prices an additional 3 to 15 percentage points, with cereals and roots and tubers seeing the largest price increases due to climate change. In the **COMP** scenario, the increases in agricultural productivity are able to not only offset the price increases caused by climate change, but also, in several cases, are able to more than match the added demands caused by population and income growth. For example, average prices for meats, cereals, pulses, roots and tubers fall to or below 2010 levels. Processed commodities like food oils and sugar, which were not targeted in this scenario, see smaller price declines, but still return to levels projected without climate change.

### 3.4 Food availability and hunger

The average kilocalorie availability across the developing world increases from about 2700 kcal per person per day in 2010 to almost 3000 per day by 2030, or the recommended daily intake of an active 20 to 35-year-old male (USDA, 2015). Africa starts from a lower base than the developing country average with 2500 kcal per person growing to 2700 by 2030. Northern and Southern Africa are above the developing country average, both achieving over 3000 kcal by 2030 with or without climate change. Eastern and Central Africa lag behind the rest of Africa with initial kilocalories per person around 2100 in 2010 and staying below 2500 in 2030 in both scenarios with and without climate change.

**Fig. 8:**
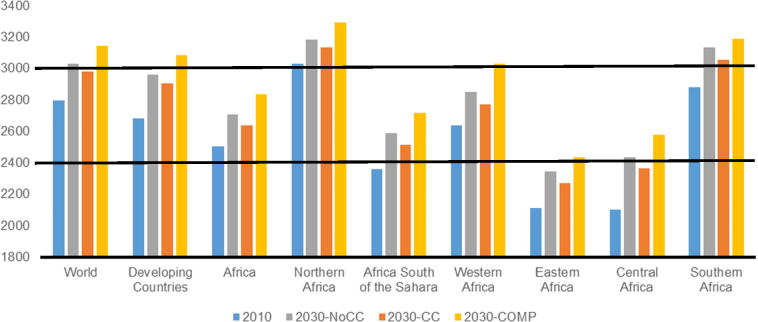
Average food supply (kilocalorie per person per day) in 2010 and 2050, by region and scenario. *Note:* The line at 1800 represents the daily minimum requirement. The black line at 2.400 represents recommended daily consumption of an active 20 to 35-year-old female and the line at 3000 represents the recommended daily consumption of an active 20–35 year old male (USDA, 2015).

Increasing incomes combined with lower food prices due to productivity improvements in the **COMP** scenario help to drive down food insecurity (along the dimension of food supply) globally with calorie availability increasing by 15 and 13 percent relative to a baseline without climate change in developing countries and Africa, respectively (Fig. 8). The largest gains are in Central and Western Africa, where kilocalories increase by six percent compared to the **NoCC** scenario. Higher consuming Northern and Southern Africa see smaller gains (two to three percent) with Eastern Africa in between at four percent.

What does it mean to achieve SDG2 and eliminate hunger? Few would argue with the goal, but actually defining it in practical terms (let alone achieving it in absolute terms) is more challenging. Changes in average income, prices, and food availability are useful targets for broadly driving down hunger but will still miss parts of the population. For example, some people remain hungry even in rich countries due to gaps in social protection measures and other factors. Therefore, we follow one of the scenarios analyzed in FAO (2015) *Achieving Zero Hunger* report, which adopted “a prudential threshold of five percent of the population.”

**Fig. 9:**
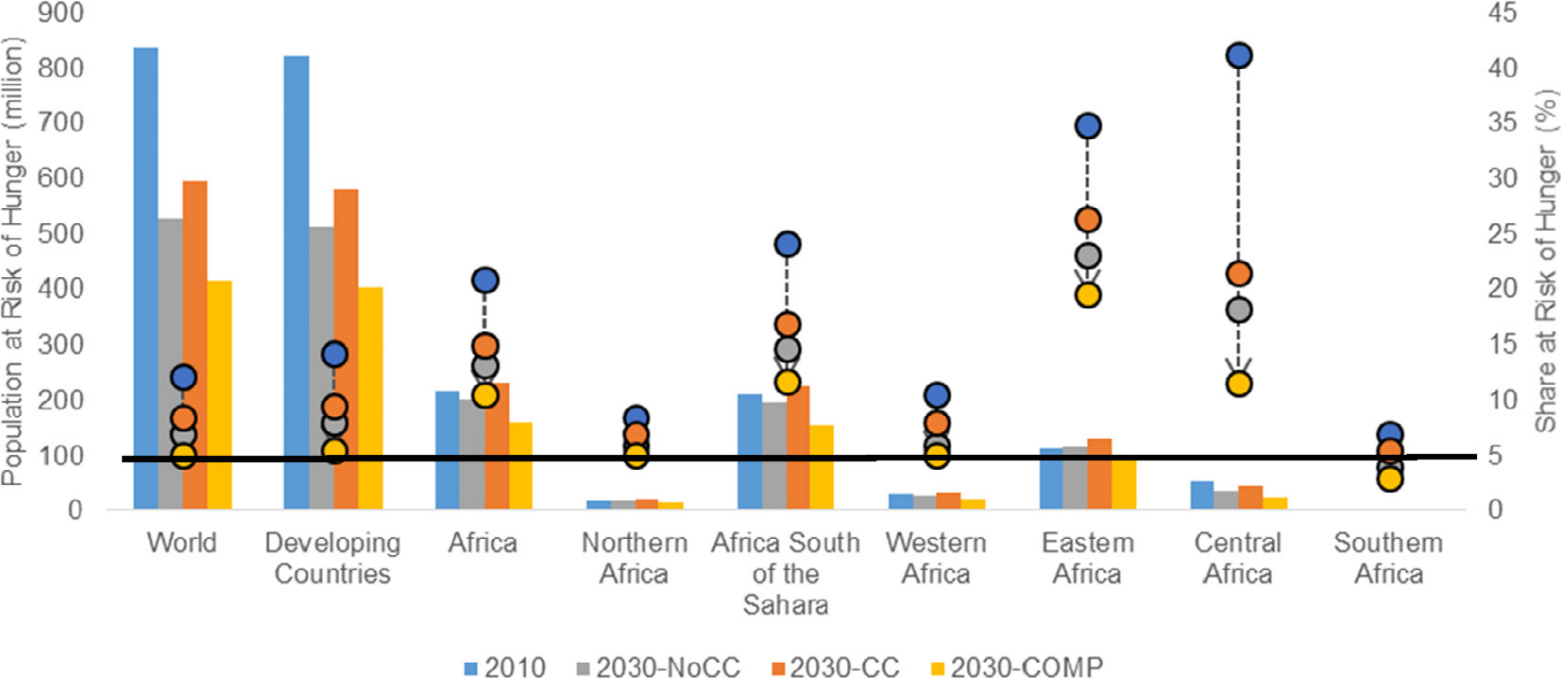
Prevalence of hunger in millions of people and as a share of the total population (%). *Note:* Clustered bars represent the number of people at risk of hunger in each region (left axis). The enlarged dots represent the share of the region’s total population at risk of hunger (right axis). The dotted lines reflect the change in the share at risk of hunger over time and across scenarios. The solid black line represents a target threshold of five percent of the population at risk of hunger (right axis).

The general trend towards increasing calorie availability leads to a decline in the prevalence of hunger as measured by the share of the total population in the developing world. However, in raw terms, this decline is less noted in Africa where rapid population growth keeps the number of people at risk of hunger in 2030 near 2010 levels even though shares are declining. This is particularly true for Northern and Eastern Africa where the number of people at risk of hunger increases in 2030 compared to 2010 even without taking into account the negative effects of climate change (Fig. 9). Once climate change is included, the population at risk of hunger in 2030 is greater than 2010 levels in Northern, Western, and Central Africa, with only Southern Africa seeing a decline in the population at risk of hunger from 2010. Nevertheless, it is important to note that in all regions the share of the population at risk of hunger is declining. Particularly large reductions are observed in Eastern and Central Africa where the share falls from 35 and 41 percent in 2010 to 26 and 22 percent in 2030, respectively.

Under the **COMP** scenario, these trends toward declining rates of hunger are accelerated and help drive down the prevalence of hunger across the three most food insecure regions (Western, Eastern, and Central Africa) that contribute almost 90 percent of those at risk of hunger in Africa and almost a quarter of the global population at risk in 2010. By 2030, the added investments in agriculture help to halve the number of people at risk of hunger and drive down the share of the world’s population at risk of hunger from over 12 percent to just over 5 percent. In Africa, these additional investments lead to the share at risk of hunger falling from 21 percent in 2010 to about 10 percent by 2030, a reduction of about 55 million people, compared to a 16 million increase by 2030 in the **CC** scenario. Three sub-regions (Northern, Western, and Southern Africa) achieve the target of five percent at risk of hunger, but the share at risk remains above 10 percent in Eastern and Central Africa, despite significant reductions. In total, 16 of the 48 African countries modeled in IMPACT approach or surpass the five percent target by 2030, with another six countries at or below 10 percent at risk of hunger. Without the additional investments in **COMP**, only 12 countries would achieve the five percent target, with another five countries below 10 percent at risk of hunger.

## 4 Discussion

### 4.1 Model results in context

Numerous estimates have been made of the cost of achieving various development goals, such as ending hunger, though methods and targets are often specified differently and therefore careful interpretation is necessary when comparing across studies. Estimates vary for different reasons related to the specific questions being asked; the objective of the study; sectors covered; whether climate change is considered; the methods, models and assumptions used; geographical coverage; and numerous other factors. Because different studies ask different questions and employ different methods, estimates are not always directly comparable. Here we compare and contrast our results with those from a 2015 study by FAO, IFAD, and WFP (referred to here as the *Achieving Zero Hunger* estimates) as well as with joint IISD and IFPRI work (Laborde, Bizikova, Lallemant, & Smaller, 2016).

Estimates of the additional investments needed are largest for the 2015 *Achieving Zero Hunger* analysis and smallest for Laborde et al. (2016) with the results presented in this paper in-between. This is largely due to differences in the questions being asked and the methods used to answer them (Table 7). The *Achieving Zero Hunger* analysis eliminates hunger in all countries by eliminating poverty. Laborde et al. (2016) instead consider the minimum public investment needed to end hunger. Both of these studies consider investments in and outside of agriculture and do not include the impacts of climate change. Our analysis, instead of costing the eradication of hunger, attempts to estimate the potential hunger reduction from investments only in the agriculture sector and includes the potential impacts of climate change on hunger eradication efforts.

**Table 7:** Comparing present analysis to other estimates of the cost of eradicating hunger.

Cost estimates	Present analysis	*Achieving Zero Hunger* (FAO, IFAD, & WFP, 2015b)	*Ending Hunger: What would it cost?* (Laborde et al., 2016)
Question asked and time frame	• What are the impacts on hunger from increases in agriculture investments in developing countries by 2030?	• What additional transfers and investments are needed to eradicate poverty and hunger in all countries by 2030?	• What is the minimum cost of ending hunger for vulnerable households in all countries by 2030?
Sectors for investment	• Increased investments in agricultural R&D, irrigation expansion, water use efficiency, soil management, and infrastructure	• Public investments in poverty gap transfers, irrigation, genetic resources, mechanization, agro-processing, infrastructure, institutions, and agricultural R&D	• Investment in social safety nets, farm support, and rural development
Climate change impacts included	• Effects of climate change are considered	• Climate change impacts are not included	• Climate change impacts are not included
Estimated annual costs	• $52 billion annually from 2015 to 30 • $15 billion of which is in Africa	• $265 billion annually from 2016 to 30 • Most of that is in Africa south of the Sahara excluding South Africa.	• $11 billion annually, of which $4 billion is from donors • Most investment in Africa.

In the 2015 *Achieving Zero Hunger* report, the Rome-based UN agencies (FAO, IFAD, & WFP, 2015a) posited that poverty and hunger could be eradicated in all developing countries by 2030. To do so would involve additional public investment in social protection combined with public and private efforts to raise investment levels in productive sectors—especially in rural areas and particularly in agriculture. Over the period 2016–2030, the additional poverty gap transfers as well as increased investments in productive sectors would need to total US$265 billion per year on average. This represents 0.3 percent of global GDP in 2014. It would fund social protection and additional targeted pro-poor investments with rural areas receiving most (US$181 billion) of the annual investments. Hunger would be reduced to below five percent prevalence rates in all developing countries considered by raising incomes of the poor above the poverty line. Climate change impacts are not considered in the *Achieving Zero Hunger* analysis.

The joint analysis by IISD and IFPRI estimates the minimum cost of ending hunger by 2030, defined as reducing the population consuming less than the Minimum Dietary Energy Requirement (MDER) to five percent or less. This study found the cost to be $11 billion annually from 2015 to 2030, four billion of which would be required from donors (Laborde et al., 2016). The pathway for ending hunger focuses on additional investments in social safety nets, farm support, and rural development. The target recipients are vulnerable households and the impacts of climate change are not included. The model used was the MIRAGRODEP model—using a mixture of multisector CGE modelling and bottom up estimates based on household survey data for seven countries in Africa. The split between donors and private financing was determined using a co-funding rule; this involved regression of government spending on ODA to determine the relationship which is assumed to stay the same into the future.

The results presented in this paper are estimates of possible outcomes of different levels of investment on a range of indicators, including agricultural productivity and hunger. Under baseline productivity scenarios, direct investments in agriculture (excluding infrastructure) across developing countries average $20 billion annually from 2010 to 2030. Including infrastructure, the total baseline investments across developing countries is about $45.5 billion. Under the **COMP** scenario, additional investments are $52 billion annually from 2015 to 2030 and are allocated to agricultural R&D, expanding irrigation, improved water use efficiency and soil management, and improved transportation and energy infrastructure. Most of the additional investments needed in agricultural R&D takes place in SSA while investments in some other sectors are more evenly spread among regions.

The impacts of additional investments under **COMP** are increased average incomes, improved agricultural productivity, decreased food prices, and improved food security. The prevalence of hunger would be reduced to five percent on average across developing countries. In addition to reducing hunger, other goals are also achieved, such as increased agricultural productivity, and increased incomes. Incomes in Africa increased by about five percent compared to the baseline in 2030, and agricultural productivity was more than 1.5 times as high as it was in 2010. Nevertheless, despite the share at risk of hunger declining to the five percent threshold in the developing world as a whole and in several African sub-regions, Africa in aggregate persists at a level closer to 10 percent with progress lagging in Eastern and Central Africa. This suggests that additional investments in agriculture could be important pieces of a broad portfolio of investments and interventions, but will need to be supplemented by additional social safety net and pro-poor investments similar to those suggested by Laborde et al. if the goal of achieving SDG2 is to be achieved.

### 4.2 Caveats and critical assessment

In this paper, we explore the potential contributions of a scenario of higher future investments within the CGIAR with a particular focus on the impacts on the population at risk of hunger. We have taken a novel approach attempting to integrate the R&D and agricultural productivity literature directly to a global economic modeling framework to better link projected economy-wide gains in agricultural production to the kinds of investments needed to achieve these gains. Our modeling efforts include a broad multi-model ensemble designed to capture key feedback dynamics between agriculture and other sectors of the economy by integration of GLOBE into the IMPACT framework. The inclusion of this linkage allowed for a better assessment of some of the potential benefits of investments in agriculture in Africa, which suggested an estimated reduction of 1.2% (2 million) fewer people at risk of hunger in Africa than would have been estimated without income feedbacks from GLOBE (for more detailed comparison of results with and without connection to GLOBE in Appendices Table S5).

Our objective with this multi-model ensemble was to expand the scope of analysis, but we recognize that results are in part dependent on the models selected. Research from AgMIP suggests that there can be significant differences in outcomes between models even when assuming the same scenario inputs (Rosenzweig et al., 2014, von Lampe et al., 2014). A multi-model ensemble that included multiple similar models (crop, economic, climate), would allow us to explore model uncertainty at each coupling point. Baseline productivity growth and investment levels, as well as future food demand are directly tied to scenarios of economic growth. This analysis only used SSP2, and we would expect the magnitude of the challenge of eradicating hunger would vary significantly based on the socioeconomic scenarios used. Nevertheless, given recent results from work by Hasegawa et al. (2018), which explores future hunger across a broader range of economic and climate scenarios, and including land-use and trade policies that were not included in this analysis, we believe our conclusion that increased investments in agriculture are necessary but insufficient to achieve SDG2 would be robust in such an exercise.

The potential gains from Agricultural R&D are also susceptible to uncertainty in future state capacity and fragility. The SSPs focus their narratives on long run trends in the global economy, which, while helpful for exploring scenarios around climate change and long run drivers in the food system like agricultural R&D, do not include other drivers that are important to global food security. For example, extreme events, both social and environmental, will almost certainly complicate progress towards SDG2. These events and crises can displace millions while destroying capital (human, physical, natural, social) and limiting the capacity of societies to effectively function. Poor governance and weak and corrupt states can prevent the effective application of science, technology, and best practices. The recent uptick in global food insecurity can be attributed in large part due to state failure and internal conflicts (FAO, 2017, FSIN, 2018). Peace and stability, broadly, are prerequisites for development, without which gains from research are unlikely to be achieved.

Uncertainty in this analysis also stems from the fact that global economic models rely on aggregated national statistics and must represent detailed economic behavior in a fairly stylized way. The global food system is rapidly transforming with the development of more complex and integrated processing value chains (Reardon et al., 2018). We believe that by coupling a highly disaggregated agricultural partial equilibrium model like IMPACT to GLOBE was an important advance to better assess economy-wide impacts of investments. However, we also recognize that the kinds of investments we considered are focused more on primary production, even as we tried to simulate certain investments like improved infrastructure that would see their impact more generally throughout the value chain. Future research linking global models like IMPACT and GLOBE to more granular analysis of value chains could highlight important complimentary interventions that could ensure there is full capacity throughout the value chain for the increased production modeled in this work. Hunger in this analysis, as well as those we’ve compared to provide context define hunger in terms of calories. For a better assessment on changes to food security, which should be the ultimate goal for policymakers, more research will be needed to move beyond calorie supply, and to consider dietary and nutritional quality. Both IMPACT and GLOBE simulate economic behavior of a single representative consumer. National averages can mask significant distributional differences within countries and there would be significant gains from trying to bridge scales of analysis through linking to more detailed value chain and disaggregated household analyses.

On the side of cost estimation, we had to work in a data sparse environment with limited long-run data on investments, which complicates the empirical estimation of returns to R&D (Esposti & Pierani, 2003). We applied a methodology with a structural theory of how investments in R&D are converted to changes in sector productivity, the PIM. However, this approach has its drawbacks in that the parameters needed to represent research lags and knowledge decay are not observed directly and are difficult to estimate as they are endogenous with respect to investment decisions (Bitzer and Stephan, 2007, Hall et al., 2010). Baker and Shittu (2007) highlighted the importance of better understanding endogenous technical change, and further research is still needed to better understand the way that research institutions respond to changes in R&D investment levels and how basic research and absorptive capacity can increase or decrease with respect to investment decisions. Better understanding these relationships will be critical for closing the modeling feedback loop, to begin to consider the potential effects of income changes and economic development on R&D investment streams.

Additionally, with our focus on public investments through the CGIAR, we assumed that private and other public investments (e.g. NARS) stayed at baseline levels and would not adjust to higher spending in the CGIAR. However, it is unlikely that the rest of the agricultural R&D ecosystem would not adjust their behavior. More research would be needed to explore this. It is possible that these increases could crowd out other investments, decreasing non-CGIAR investments, which in turn could diminish regional capacity to fully benefit from research done in the CGIAR. Alternatively, it could spur complementary research investments, leading to an even greater impact from additional investments. From a policy perspective, the latter case is obviously preferred, so applied research in this realm would be useful to ensure greater impacts and efficacy.

## 5 Conclusions

In this research, we used a quantitative foresight modeling system (IMPACT) to examine the potential impact of increasing investments in agricultural R&D, with a particular focus on the potential impact of climate change. Agricultural research, resource management, and infrastructure improvement can help address the challenges of meeting future food demands, but it is unlikely that hunger can be eliminated by focusing on the agricultural sector alone. Complementary efforts will be required outside of agriculture. The costs of investments focused on the agricultural sector are in line with historical trends and should be manageable with increasing growth in global and regional economies. Nevertheless, reducing hunger in Africa will be a challenge moving forward. To make progress towards this goal we should recognize the following:

1. *Climate change has impeded and will continue to impede progress on reducing hunger*.
  Climate change could increase the number of people at risk of hunger in 2030 relative to a scenario without climate change. In Africa, the combined effects of population growth and climate change mean that the number of people at risk of hunger is projected to increase even relative to today’s levels, i.e. more than offsetting reductions projected in 2030 due to increased incomes and other factors. Reducing hunger will be particularly challenging in Eastern and Central Africa.
2. *Increased investment in agriculture in Africa and the rest of the world can accelerate productivity growth for important crops and livestock and counteract the effects of climate change*.
  Increased investments in agricultural research, resource management, and infrastructure can more than offset the negative effects of climate change, increasing crop productivity in 2030 by 40 percent globally and by over 50 percent in Africa compared to 2010 levels.
3. *Increased investments in agricultural R&D will result in large declines in the share and number of hungry people in Africa*.
  Increased productivity growth in agriculture boosts food supply, reduces food prices, and increases incomes. This increases access to food and helps reduce the share of people at risk of hunger in 2030 to five percent or less in Northern, Western, and Southern Africa. However, the share is projected to remain at 10 percent or more in Eastern and Central Africa even with investments in agricultural R&D.
4. *Additional investments in Africa to achieve these results under climate change are estimated to cost about $15 billion per year between 2015 and 2030*^4^ *(compared to the baseline scenario).*
  While the costing methodology in this research produced estimates that differ from other research on this topic, the research questions and approaches make these studies mutually reinforcing, despite their apparent contrast. The joint IISD and IFPRI work by Laborde et al. (2016) targeted vulnerable households only and did not consider the impacts of climate change and produced lower estimate for annual cost. The FAO et al. (2015a) *Achieving Zero Hunger* report estimated a costlier goal of raising incomes and eliminating poverty more generally. In all cases, the research implies necessary increases over current levels of government and international spending on agriculture in Africa.
5. *Increased investment outside of the agricultural sector is likely needed to end hunger in Africa by 2030*.
  While agriculture is an important cornerstone for most of Africa, its overall importance is declining as economies grow. Investments in the agricultural sector will help build the foundation for a more prosperous and stable Africa, but complementary investments throughout value chains within the food system and in the non-agricultural sector will be necessary for resilient economies and achievement of the SDGs, especially for eliminating hunger. Future work will examine the scope for further progress in ending hunger through investments outside of the agricultural sector, such as clean water and sanitation, health, education, and the manufacturing and services sectors.

## Conflicts of interest

Several authors are or were employed by the International Food Policy Research Institute, which is a member of the CGIAR. Non-IFPRI authors declare no interests.

## Footnotes

1 While these investment numbers are estimates of total amounts subject to different methods of imperfect data collection, they do provide a fair indication of equivalence and relative magnitude.

2 To test the sensitivity of the climate model selected, we simulated scenarios under an additional climate model from the Institut Pierre Simon Laplace’s Earth System Model (Dufresne et al., 2013) and found that our results were robust across this alternative climate realization.

3 Due to data constraints, it is not possible to separate West Asia from the aggregated numbers for West Asia and North Africa (WANA).

## Footnotes

4 As part of a larger, global package of additional investments costing around USD 52 billion, compared to the baseline scenario, in developing countries as a whole.

## References

[b0005] Aghion P., Jaravel X. (2015). Knowledge spillovers, innovation and growth. The Economic Journal.

[b0010] Alston J.M. (2018). Reflections on agricultural R&D, productivity, and the data constraint: unfinished business, unsettled issues. American Journal of Agricultural Economics.

[b0015] Alston J.M., Andersen M.A., James J.S., Pardey P.G. (2011). The economic returns to U.S. public agricultural research. American Journal of Agricultural Economics.

[b0020] Alston J.M., Pardey P.G. (2001). Attribution and other problems in assessing the returns to agricultural R&D. Agricultural Economics.

[b0025] Alston J.M., Pardey P.G., James J.S., Andersen M.A. (2009). A Review of Research on the Economics of Agricultural R&D. Annual Reviews of Resource Economics.

[b0030] ASTI (Agricultural Science and Technology Indicators) (2016). ASTI Database.

[b0035] Baker E., Shittu E. (2007). Uncertainty and endogenous technical change in climate policy models. Energy Economics.

[b0040] Ball E., Schimmelpfenning D., Wang S.L. (2013). Is U.S. agricultural productivity growth slowing?. Applied Economic Perspectives and Policy.

[b0045] Beintema N., Stads G., Fuglie K., Heisey P. (2012). ASTI global assessment of agricultural R&D spending.

[b0050] Bitzer J., Stephan A. (2007). A Schumpeter-inspired approach to the construction of R&D capital stocks. Applied Economics.

[b0055] Bodirsky B.L., Rolinski S., Biewald A., Weindl I., Popp A., Lotze-Campen H. (2015). Global food demand scenarios for the 21st century. PLoS ONE.

[b0060] Buchner, B.K., Trabacchi, C., Mazza, F., Abramskiehn, D., & Wang, D. (2015). Global landscape of climate finance 2015: A CPI report. Available at: http://climatepolicyinitiative.org/wp-content/uploads/2015/11/Global-Landscape-of-Climate-Finance-2015.pdf.

[b0065] Chandy L., Kato H., Kharas H., Chandy L., Kato H., Kharas H. (2015). From a billion to zero: Three key ingredients to end extreme poverty. The last mile in ending extreme poverty.

[b0070] Clarke L., Edmonds J., Jacoby H., Pitcher H., Reilly J., Richels R. (2007). Scenarios of Greenhouse Gas Emissions and Atmospheric Concentrations. Sub-report 2.1A of Synthesis and Assessment Product 2.1 by the U.S. Climate Change Science Program and the Subcommittee on Global Change Research.

[b0075] Delgado C.L., Rosegrant M.W., Steinfeld H., Ehui S., Courbois C. (2001). Livestock to 2020: The next food revolution. Outlook on Agriculture.

[b0080] Dellink R., Chateau J., Lanzi E., Magné B. (2017). Long-term economic growth projections in the share socioeconomic pathways. Global Environmental Change.

[b0085] Dercon S., Gollin D. (2014). Agriculture in African development: Theories and strategies. Annual Review of Resource Economics.

[b0090] Dufresne J.L., Foujols M.A., Denvil S., Caubel A., Marti O., Aumont O., Vuichard N. (2013). Climate change projections using the IPSL-CM5 earth system model: From CMIP3 to CMIP5. Climate Dynamics.

[b0095] Esposti M., Pierani F. (2003). Building the knowledge stock: Lags, depreciation, and uncertainty in R&D investment and link with productivity growth. Journal of Productivity Analysis.

[b0100] Evenson R.E., Gollin D. (2003). Assessing the impact of the Green Revolution, 1960 to 2000. Science.

[b0105] Evenson R.E., Rosegrant M.W., Evenson R.E., Gollin D. (2003). The economic consequences of crop genetic improvement programmes. Crop variety improvement and its effect on Productivity: The impact of international agricultural research.

[b0110] FAO (2012). The state of food and agriculture: Investing in agriculture for a better future.

[b0115] FAO (2015). Regional overview of food insecurity: African food security prospects brighter than ever.

[b0120] FAO (2016). FAOSTAT. Online statistical database.

[b0125] FAO (2016). AQUASTAT.

[b0130] FAO (2017). The state of food security and nutrition in the world 2017: Building resilience for peace and food security.

[b0135] FAO, IFAD, WFP (2015). The State of Food Insecurity in the World 2015. Meeting the 2015 international hunger targets: taking stock of uneven progress.

[b0140] FAO, IFAD, WFP (2015). Achieving Zero Hunger: The critical role of investments in social protection and agriculture.

[b0145] Fischer G., Shah M., Tubiello F.N., van Velhuizen H. (2005). Socio-economic and climate change impacts on agriculture: An integrated assessment. Philosophical Transactions of the Royal Society B.

[b0150] FSIN. (2018). Global report on food crisis 2018. Retrieved from https://www.wfp.org/content/global-report-food-crises-2018.

[b0155] Fuglie K.O., Fuglie K.O., Wang S.L., Ball V.E. (2012). Productivity growth and technology capital in global agriculture. Productivity growth in agriculture: An international perspective.

[b0160] Fuglie K.O. (2017). R&D capital, R&D spillovers, and productivity growth in world agriculture. Applied Economic Perspectives and Policy.

[b0165] Fujino J., Nair R., Kainuma M., Masui T., Matsuoka Y. (2006). Multi-gas mitigation analysis on stabilization scenarios using AIM global model. Multigas Mitigation and Climate Policy. The Energy Journal.

[b0170] Griliches Z. (1958). Research cost and social returns: Hybrid corn and related innovations. Journal of Political Economy.

[b0175] Griliches Z. (1979). Issues in Assessing the contribution of research and development to productivity growth. The Bell Journal of Economics.

[b0180] Griliches Z. (1994). Productivity, R7D, and the data constraint. American Economic Review.

[b0185] GYGWPA (2017). Global yield gap and water productivity Atlas. Available at: www.yieldgap.org. (Accessed 2/5/2017).

[b0190] Hall B.H., Mairesse J., Mohnen P., Hall B.H., Rosenberg N. (2010). Measuring the returns to R&D.

[b9015] Hasegawa T., Fujimori S., Havlik P., Valin H., Bodirsky B., Doelman J., Witzke P. (2018). Risk of Increased Food Insecurity under Stringent Global Climate Change Mitigation Policy. Nature Climate Change.

[b0200] Hijioka Y., Matsuoka Y., Nishimoto H., Masui M., Kainuma M. (2008). Global GHG emissions scenarios under GHG concentration stabilization targets. Journal of Global Environmental Engineering.

[b0205] Hoogenboom G., Jones J.W., Wilkens P.W., Porter C.H., Boote K.J., Hunt L.A., Koo J. (2012). Decision support system for Agrotechnology Transfer (DSSAT) Version 4.5.

[b0210] Huffman W.E., Kalaitzandonakes N., Carayannis E., Grigoroudis E., Rozakis S. (2018). Public agricultural research and its contributions to agricultural productivity. From agriscience to agribusiness. Innovation, technology, and knowledge management.

[b0220] IFPRI. (2015). Statistics on Public Expenditures for Economic Development (SPEED). doi: 10.7910/DVN/INZ3QK.

[b0225] Ignaciuk A., D’Croz D., Islam S. (2015). Better drip than flood: Reaping the benefits of efficient irrigation. EuroChoices.

[b0230] Ignaciuk, A., & Mason-D'Croz, D. (2014). Modelling adaptation to climate change in agriculture. OECD Food, Agriculture and Fisheries Papers, No. 70, OECD Publishing. doi: 10.1787/5jxrclljnbxq-en.

[b0235] IIASA (2013) SSP Database version 1.0. Accessed at: https://tntcat.iiasa.ac.at/SspDb (Accessed on 2015-05-10 23:13:16).

[b0240] IIASA (2015) RCP Database version 2.0.5. Accessed at: http://www.iiasa.ac.at/web-apps/tnt/RcpDb (Accessed on 2015-05-10 23:13:16).

[b0245] Inocencio A., Kikuchi M., Tonosaki M., Maruyama A., Merrey D., Sally H., de Jong I. (2007). Cost of performance of irrigation projects: A comparison of Sub-Saharan Africa and other developing regions.

[b0250] IPCC (2013). Climate Change 2013: The Physical Science Basis. Contribution of Working Group I to the Fifth Assessment Report of the Intergovernmental Panel on Climate Change.

[b0255] Islam S., Cenacchi N., Sulser T.B., Hareau G., Kleinwechter U., Mason-D’Croz D., Wiebe K. (2016). Structural frameworks for modeling climate change adaptation technologies for food security. Global Food Security.

[b0260] Jiang L., O’Neill B.C. (2017). Global urbanization projections for the shared socioeconomic pathways. Global Environmental Change.

[b0265] Jones J.W., Hoogenboom G., Porter C.H., Boote K.J., Batchelor W.D., Hunt L.A., Ritchie J.T. (2003). DSSAT cropping system model. European Journal of Agronomy.

[b0270] Jones C.D., Hughes J.K., Bellouin N., Hardiman S.C., Jones G.S., Knight J., Zerroukat M. (2011). The HadGEM2-ES implementation of CMIP5 centennial simulations. Geoscientific Model Development.

[b0275] Kc S., Lutz W. (2017). The human core of the shared socioeconomic pathways: Population scenarios by age, sex, and level of education for all countries to 2100. Global Environmental Change.

[b0280] Laborde D., Bizikova L., Lallemant T., Smaller C. (2016). Ending Hunger: What would it cost?.

[b0285] Lowder S., Carisma B., Skoet J. (2015). Who invests how much in agriculture in low- and middle-income countries? An empirical review. European Journal of Development Research.

[b0290] Markowitz H.M. (1952). Portfolio selection. Journal of Finance.

[b0295] Markowitz H.M. (1991). Foundations of portfolio theory. Journal of Finance.

[b0300] Mason-D’Croz D., Vervoort J., Palazzo A., Islam S., Lord S., Helfgott A., Lipper L. (2016). Multi-factor, multi-state, multi-model scenarios: Exploring food and climate futures for Southeast Asia. Environmental Modelling and Software.

[b0305] McArthur J.W., Chandy L., Kato H., Kharas H. (2015). Agriculture’s role in ending extreme poverty. The last mile in ending extreme poverty.

[b0310] McDonald S., Thierfelder K., Robinson S. (2007). Globe: A SAM Based Global CGE Model using GTAP Data.

[b0315] Moss R.H., Edmonds J.A., Hibbard K.A., Manning M.R., Rose S.K., van Vuuren D.P., Wilbanks T.J. (2010). The next generation of scenarios for climate change research and assessment. Nature.

[b0320] Narayanan B., Aguiar A., McDougall R. (2012). Global trade, assistance, and production: The GTAP 8 data base.

[b0325] Nelson G.C., Rosegrant M.W., Palazzo A., Gray I., Ingersoll C., Robertson R., Msangi S. (2010). Food security, farming, and climate change to 2050: Scenarios, results, policy options.

[b0330] Nelson G.C., Valin H., Sands R.D., Havlík P., Ahammad H., Deryng D., Willenbockel D. (2014). Climate change effects on agriculture: Economic responses to biophysical shocks. Proceedings of the National Academy of Science.

[b0340] Nin-Pratt A., Greene W.H., Khalaf L., Sickles R.C., Veall M., Voia M.C. (2016). Inputs, productivity and agricultural growth in sub-Saharan Africa. Productivity and efficiency analysis.

[b0345] Nin-Pratt A., Falconi C., Ludena C., Martel P. (2015). Productivity and the performance of agriculture in Latin America and the Caribbean: From the lost decade to the commodity boom.

[b0350] Nordmann A. (2014). Responsible innovation, the art and craft of anticipation. Journal of Responsible Innovation.

[b0355] O’Neill B.C., Kriegler E., Ebi K.L., Kemp-Benedict E., Riahi K., Rothman D.S., Solecki W. (2017). The roads ahead: Narratives for shared socioeconomic pathways describing world futures in the 21st century. Global Environmental Change.

[b0360] ODI (2015). Climate Funds Update dataset. Available at: http://www.climatefundsupdate.org.

[b0365] OECD. (2016). Climate change: OECD DAC external development finance statistics: Project level data for every climate-related development finance project in 2013–14. Available at: http://www.oecd.org/dac/stats/climate-change.htm.

[b0370] O'Neill B.C., Kriegler E., Riahi K., Ebi K.L., Hallegatte S., Carter T.R., van Vuuren D.P. (2014). A new scenario framework for climate change research: The concept of shared socioeconomic pathways. Climatic Change.

[b9020] Palazzo A., Vervoort J.M., Mason-D’Croz D., Rutting L., Havlík P., Islam S., Zougmore R. (2017). Linking regional stakeholder scenarios and shared socioeconomic pathways: Quantified West African food and climate futures in a global context. Global Environmental Change.

[b0375] Pingali P. (2007). Westernization of Asian diets and the transformation of food systems: Implications for research and policy. Food Policy.

[b0380] Pingali P. (2012). Green revolution: Impacts, limits, and the path ahead. Proceedings of the National Academy of Science.

[b0385] Reardon T., Echeverria R., Berdegue J., Minten B., Liverpool-Tasie S., Tschirley D., Zilberman D. (2018). Rapid transformation of food systems in developing regions: Highlighting the role of agricultural research & innovations. Agricultural Systems.

[b0390] Renkow M., Byerlee D. (2010). The impacts of CGIAR research: A review of recent evidence. Food Policy.

[b0395] Riahi, K., & Nakicenovic, N. (eds.) (2007). Greenhouse gases – integrated assessment, technological forecasting and social change, Special Issue, 74(7), September 2007, p. 234 (ISSN 0040-1625).

[b0400] Robinson S., Mason-D’Croz D., Islam S., Sulser T.B., Robertson R., Zhu T., Rosegrant M.W. (2015). The International Model for Policy Analysis of Agricultural Commodities and Trade (IMPACT): Model description, Version 3..

[b0405] Robinson S., van Meijl H., Willenbockel D., Valin H., Fujimori S., Masui T., von Lampe M. (2014). Comparing supply-side specifications in models of global agriculture and the food system. Agricultural Economics.

[b0410] Rockström J., Steffen W., Noone K., Persson Å., Chapin F.S., Lambin E., Foley J. (2009). Planetary boundaries: Exploring the safe operating space for humanity. Ecology and Society.

[b0415] Rosegrant M.W., Cai X., Cline S.A. (2002). World water and food to 2025: Dealing with scarcity.

[b0425] Rosegrant M.W., Koo J., Cenacchi N., Ringler C., Robertson R.D., Fisher M., Sabbagh P. (2014). Food security in a world of natural resource scarcity: The role of agricultural technologies.

[b0430] Rosegrant M.W., Leach N., Gerpacio R.V. (1999). Alternative futures for world cereal and meat consumption. Nutrition Society.

[b0435] Rosegrant M., Magalhaes E., Valmonte-Santos R., Mason-D’Croz D., Lomborg B. (2018). Returns to investment in reducing postharvest food losses and increasing agricultural productivity growth. Prioritizing development: A cost benefit analysis of the United Nations' sustainable development goals.

[b0440] Rosegrant M.W., Paisner M.S., Meijer S., Witcover J. (2001). 2020 Global food outlook: Trends and alternative futures.

[b0445] Rosegrant M.W., Sulser T.B., Mason-D’Croz D., Cenacchi N., Nin-Pratt A., Dunston S., Willaarts B. (2017). Quantitative foresight modeling to inform the CGIAR research portfolio. Project Report for USAID.

[b0450] Rosenzweig C., Elliott J., Deryng D., Ruane A.C., Müller C., Arneth A., Neumann K. (2014). Assessing agricultural risks of climate change in the 21st century in a global gridded crop model intercomparison. Proceedings of the National Academy of Sciences.

[b0455] Ruane A.C., Antle J., Elliott J., Folberth C., Hoogenboom G., Mason-D’Croz D., Rosenzweig C. (2018). Biophysical and economic implications for agriculture of +1.5 and +2.0 °C global warming using AgMIP coordinated global and regional assessments. Climate Research.

[b0460] Schumpeter J. (1942). The process of creative destruction. Chapter 7 in capitalism, socialism, and democracy.

[b0465] Smith L.C., Haddad L.J. (2000). Explaining child malnutrition in developing countries: A cross-country analysis.

[b0470] Smith S.J., Wigley J.M.L. (2006). Multi-gas forcing stabilization with MiniCam. The Energy Journal.

[b0475] Springmann M., Mason-D’Croz D., Robinson S., Garnett T., Godfray H.C.J., Gollin D., Scarborough P. (2016). Global and regional health effects of future food production under climate change. The Lancet.

[b0480] Sulser T.B., Mason-D’Croz D., Islam S., Robinson S., Wiebe K., Rosegrant M.W., Badiane O., Makombe T., Benin S. (2015). Africa in the global agricultural economy in 2030 and 2050. Towards a middle income Africa: Long term growth outlook and strategies. ReSAKSS annual trends and outlook report 2014.

[b0485] Sulser T.B., Nestorova B., Rosegrant M.W., van Rheenen T. (2011). The future role of agriculture in the Arab region’s food security. Food Security.

[b0490] Tassey G. (2005). Underinvestment in public good technologies. Journal of Technology Transfer.

[b0495] Taylor K.E., Stouffer R.J., Meehl G.A. (2012). An overview of CMIP5 and the experiment design. Bulletin of the American Meteorological Society.

[b0500] Tilman D., Clark M. (2014). Global diets link environmental sustainability and human health. Nature.

[b0505] Timmer C.P. (2002). Agriculture and economic development. Handbook of Agricultural Economics.

[b0510] UNEP (2016). The Adaptation Finance Gap Report (2016).

[b0515] United Nations (2015). The millennium development goals report 2009.

[b0520] United Nations (2015). Transforming our world: The 2030 agenda for sustainable development.

[b0525] USDA. (2015). USDA food patterns – Estimated calorie deeds per day – Energy levels used for assignment of individuals to USDA Food Patterns. Accessed at: https://www.cnpp.usda.gov/sites/default/files/usda_food_patterns/EstimatedCalorieNeedsPerDay.pdf.

[b0530] van Ittersum M., Bussel L.G.J., Wolf J., van Wart J., Guilpart N., Claessen L., Cassman K.G. (2016). Can sub-Saharan Africa feed itself?. Proceedings of the National Academy of Sciences.

[b0535] van Vuuren D., den Elzen M., Lucas P., Eickhout B., Strengers B., van Ruijven B., van Houdt R. (2007). Stabilizing greenhouse gas concentrations at low levels: An assessment of reduction strategies and costs. Climatic Change.

[b0540] van Vuuren D.P., Eickhout B., Lucas P.L., den Elzen M.G.J. (2006). Long-term multi-gas scenarios to stabilise radiative forcing – Exploring costs and benefits within an integrated assessment framework. The Energy Journal.

[b0545] Vervoort J., Gupta A. (2018). Anticipating climate futures in a 1.5 °C era: The link between foresight and governance. Current Opinion in Environmental Sustainability.

[b0550] Vervoort J.M., Thornton P.K., Kristjanson P., Förch W., Ericksen P.J., Kok K., Jost C. (2014). Challenges to scenario-guided adaptive action on food security under climate change. Global Environmental Change.

[b0555] von Lampe M., Willenbockel D., Ahammad H., Blanc E., Cai Y., Calvin K., van Meijl H. (2014). Why do global long-term scenarios for agriculture differ? An overview of the AgMIP Global Economic Model Intercomparison. Agricultural Economics.

[b0560] Warszawski L., Frieler K., Huber V., Piontek F., Serdeczny O., Schewe J. (2014). The Inter-Sectoral Impact Model Intercomparison Project (ISI-MIP): Project framework. Proceedings of the National Academy of Sciences.

[b0565] Wiebe K., Lotze-Campen H., Sands R., Tabeau A., van der Meensbrugghe D., Biewald A., Willenbockel D. (2015). Climate change impacts on agriculture in 2050 under a range of plausible socioeconomic and emissions scenarios. Environmental Research Letters.

[b0570] Willenbockel, D., Robertson, R. D., Mason-D’Croz, D., Rosegrant, M. W., Sulser, T., Dunston, S., & Cenacchi, N. (2018). Dynamic computable general equilibrium simulations in support of quantitative foresight modeling to inform the CGIAR research portfolio: Linking the IMPACT and GLOBE models. (IFPRI Discussion Papers No. 1738). Washington D.C. Retrieved from: http://ebrary.ifpri.org/cdm/ref/collection/p15738coll2/id/132757.

[b0575] Wise M.A., Calvin K.V., Thomson A.M., Clarke L.E., Bond-Lamberty B., Sands R.D., Edmonds J.A. (2009). Implications of limiting CO_2_ concentrations for land use and energy. Science.

