# Supplementary material for: Agricultural investments and hunger in Africa modeling potential contributions to SDG2 – Zero Hunger
Source: World Dev. 2019 Apr;116:38–53. doi: 10.1016/j.worlddev.2018.12.006 (PMC6358118; doi:10.1016/j.worlddev.2018.12.006)
Supplement: Supplementary Data 1 [file mmc1.docx]

Supplementary Appendices

Supplement to: “Agriculture investments and hunger in Africa; Modeling potential contributions to SDG2 – Zero Hunger”

#

Table of Contents

[S1. Definition of Private Investment in Agriculture 1](#_Toc522567497)

[S2. Summary Description of IMPACT 2](#_Toc522567498)

[S3. Summary Description of GLOBE-Energy 18](#_Toc522567499)

[S4. Linking IMPACT to GLOBE 33](#_Toc522567500)

[S5. Agricultural Research and Productivity Growth 39](#_Toc522567501)

[S6. Costing Irrigation Expansion and Improvement 43](#_Toc522567502)

[S7. Infrastructure Investment Costs 45](#_Toc522567503)

# Definition of Private Investment in Agriculture

The estimates of private investment in agriculture, or gross fixed capital formation (GFCF), are from a new agricultural capital stock dataset recently made available on FAOSTAT which follows the system of national accounts approach (United Nations et al., 1993 and 2009). Agriculture refers to crop production, livestock, forestry, and fisheries, corresponding to ISIC (revision 3) codes A and B (United Nations, 2016). Investment, or GFCF, is defined as the value of a producer’s acquisitions and improvements to assets (such as improvements to structures, buildings, or land) minus disposals of assets. Assets included in the estimation are those used for one year or more, including farm buildings and structures, machinery and equipment, livestock, land improvements, tree crops, and other perennials. Hand tools are not included in the estimation unless they are purchased infrequently and represent the majority of assets in a country.

The new FAOSTAT estimates of investment in agriculture are conservative since they are made using only national accounts. For many countries, treestock and livestock are not included in national accounts and, as such, the new FAOSTAT estimates are far lower than those previously released on FAOSTAT. Several researchers have estimated investment in agriculture by supplementing national accounts estimates with inventory-based estimates of treestock and livestock (see Crego et al., 1998; Larson et al., 2011; and Anriquez and Daidone, 2011).

# Summary Description of IMPACT

The following description is a summary of the documentation for the International Model for Policy Analysis for Agricultural Commodities and Trade (IMPACT; Robinson et al., 2015), which is available in full at: <http://ebrary.ifpri.org/cdm/ref/collection/p15738coll2/id/129825>

IMPACT is a system of linked models centered on a multimarket, partial equilibrium representation of the agriculture sector. The multimarket model simulates the operation of national and international markets, solving for production, demand, and prices that equate supply and demand across the globe through net trade. The core model is linked to a number of modules that include climate, water (hydrology, water basin management, and water stress models), crop simulation, value chain (e.g. sugar, oils, livestock), and land use models. IMPACT simulates global markets for 62 agricultural commodities through 158 linked national markets. Region and commodity aggregations used in this analysis are defined in Table S1 and Table S2 respectively.

## Crop Production

Crop production in IMPACT is simulated through area[[1]](#footnote-1) and yield response functions. The choice of specifying crop production in this way has a long history in IMPACT and facilitates interaction with commodity experts and land-use specialists, who work in natural units (hectares, tons per hectare). Crop production in IMPACT is specified sub-nationally with the area and yield functions at the level of 320 food production unit (FPUs)[[2]](#footnote-2). This regional disaggregation permits linking with water models and provides the added benefit of smaller geographical units for aggregating climate change results, which can vary significantly from one location to another. Land used for crop production is divided into irrigated and rainfed systems, capturing the significant differences in yields observed across these cultivation systems and linking directly with the water models, which treat irrigated and rainfed water supplies separately.

IMPACT version 3 simulates simplified land markets to manage competing demands for agricultural land from different crops. It allows total area supply to be determined separately from individual crop area demands and allows equilibrium conditions to determine the best economic use of the available land. The total supply of land is assumed to be a function of the scarcity value or shadow price index of land, which can also be considered a summary of changes in crop prices. The shadow price (WF) is indexed to 1 in the first year and changes based on changing demands from all crops for land area.

$$\begin{matrix} QFS_{\text{fpu,lnd}}=QFSInt_{\text{fpu,lnd}}\times QFSInt2_{\text{fpu,lnd}} \\ QFS=\text{Land supply} \\ QFSInt=\text{Land supply intercept (base year supply)} \\ QFSInt2=\text{Land supply growth multiplier} \\ fpu=\text{Food production unit} \\ lnd=\text{Land type (i.e. irrigated, rainfed)} \end{matrix}$$

The supply of land is considered exogenous within each year, meaning that farmers are not allowed to adjust the total crop area in the middle of the year. The total land supply over time is driven by exogenous trends on the availability of area for agriculture as well as endogenous responses to changes in area demand, which is handled in between years. The following equation is applied at the end of each year before solving for a new year.

Crop area is specified as an area demand function with respect to changes in the marginal revenue product, changes in land cost, and exogenous non-price trends in harvested area. Crop area elasticities simulate the supply response to changes in the marginal revenue of land represented by the following equation as the interaction of the net price of an activity and the productivity of the activity in using an additional hectare of land.

$$\begin{matrix} MRP_{\text{j,fpu,lnd}}=Yld_{\text{j,fpu,lnd}}\times PNET_{\text{j,cty}} \\ MRP=\text{Marginal revenue product of land} \\ Yld=\text{Crop yield} \\ PNET=\text{Net price for the activity at the country-level mapped to fpu} \\ j=\text{ Activity (crop)} \\ cty=\text{Country} \end{matrix}$$

The exogenous trend in harvested area captures changes in area resulting from factors other than direct market effects, such as government programs encouraging cropping expansion, contraction due to soil degradation, or conversion of land from agriculture to nonagricultural uses. The combination of these endogenous and exogenous factors in area demand are described in the following equation.

$$\begin{matrix} Area_{\text{j,fpu,lnd}}=AreaInt_{\text{j,fpu,lnd}}\times AreaInt2_{\text{j,fpu,lnd}}\times WF_{\text{fpu,lnd}}^{WF\varepsilon}\times\left(\frac{MRP_{\text{j,fpu}}}{MRP0_{\text{j,fpu}}}\right)^{A\varepsilon} \\ Area=\text{Final crop area} \\ AreaInt=\text{Crop area intercept (base year crop area)} \\ AreaInt2=\text{Exogenous crop area growth multiplier} \\ WF\varepsilon=\text{Elasticity of demand with respect to land shadow price} \\ MRP0=\text{Base year marginal revenue product (used to index prices)} \\ A\varepsilon=\text{Elasticity of area demand with respect to marginal revenue product} \end{matrix}$$

Assumptions for exogenous trends are determined by a combination of historical changes in land use and expert judgment on potential future regional dynamics. They are represented as compound growth from the base and are applied between years.

Competing demands from different crops are handled through an equilibrium equation that determines land allocation and ensures that all crop area demand sums up to the total land supply for each FPU.

$$QFS_{fpu,lnd}=\sum_{j} Area_{j,fpu,lnd}$$

Crop yields are a function of commodity prices, prices of inputs, available water, climate, and exogenous trend factors. The IMPACT model includes five ways that changes in yields are achieved. First, the model assumes a scenario of underlying improvements in yields over time that include, to varying degrees, continuation of past trends and expert opinion on future yield potential. These long-run trends, or intrinsic productivity growth rates (IPRs), are intended to reflect the expected increases in inputs, improved seeds, and improvements in management practices. These underlying trends vary by crop and region and are assumed to level off somewhat during the next 50 years as the pace of technological improvements in developed countries slows and as developing countries catch up to yield potentials in developed countries.

Second, the IMPACT model includes a short-run (annual), endogenous, response of yields to changes in both input and output prices. These yield response functions specify the change in yield as a constant elasticity function of the changes in output prices, with elasticity parameters that can vary by crop and region. The underlying assumption is that farmers will respond to changes in prices by varying the use of inputs, such as fertilizer, chemicals, and labor that will, in turn, change yields.

Third, climate is assumed to affect yields through two mechanisms. The first is through the effects of changes in temperature and weather due to climate change on crop yields for rainfed and irrigated crops, as calculated from the solution of a crop simulation model (DSSAT, see Hoogenboom et al., 2012; Jones et al., 2003) for different climate change scenarios and different crops. The DSSAT model is run with detailed time, geographic, and crop disaggregation for different climate change scenarios that are downscaled to include weather variation in small geographic areas. This analysis gives changes in average yields due to climate change that are then averaged to generate yield shocks by crop and region (FPU) in the IMPACT model. These long-run climate scenarios generate yield shocks that are assumed to follow simple trends over time and do not consider extreme events such as droughts.

The fourth mechanism by which yields are affected is through variation in water availability for agriculture year by year in different climate scenarios. This mechanism is modeled through the use of the IMPACT water models. These include (1) a global hydrology model that determines runoff to the river basins included in the IMPACT model; (2) water basin management models for each FPU that optimally allocate available water to competing nonagricultural and agricultural uses, including irrigation; and (3) a water allocation and stress model that allocates available irrigation water to crops and, when the water supply is less than demand by crop, computes the impact of the water shortage on crop yields, accounting for differences among crops and varieties. These yields shocks are then passed to the IMPACT model, affecting year-to-year crop yields.

$$\begin{matrix} Yield_{\text{j,fpu,lnd}}=YieldInt_{\text{j,fpu,lnd}}\times YieldInt2_{\text{j,fpu,lnd}}\times WatShk_{\text{j,fpu,lnd}}\times CliShk_{\text{j,fpu,lnd}}\times\left(\frac{PNET_{\text{j,cty}}}{PNET0_{\text{j,cty}}}\right)^{Y\varepsilon}\times PF^{F\varepsilon} \\ Yield=\text{Final yield} \\ YieldInt=\text{Yield intercept (base year yield)} \\ YieldInt2=\text{Exogenous yield growth multiplier} \\ WatShk=\text{Water stress shock (from water models)} \\ CliShk=\text{Climate change shock (from water and crop models)} \\ Y\varepsilon=\text{Yield supply elasticity with respect to net price} \\ PF=\text{Input prices} \\ F\varepsilon=\text{Yield supply elasticity with respect to input prices} \end{matrix}$$

Final crop production for each FPU and crop (j) is estimated as the product of the solution for its respective area and yield equations, with national production (*QS*_j,cty_) equal to the summation of the production in all of the relevant FPUs in that country.

$$QS_{\text{j,cty}}=\sum_{\text{fpu,lnd}} \left(Area_{\text{j,fpu,lnd}}\times Yield_{\text{j,fpu,lnd}}\right)$$

## Livestock Production

Livestock production is modeled at the FPU level and includes animal numbers, with associated feed demands, and meat/dairy production based on processing. Similar to the crop sector, this specification allows for easier translation of information from livestock experts who are used to working with herd-size and feeding requirements. In the current version of the model herd size over time is set exogenously.

Feed demand is a function of the livestock’s own price, the prices of intermediate (feed) inputs, and a trend variable reflecting growth in livestock herds (slaughter rates are implicitly assumed to stay more or less constant over time). The price elasticities in the livestock supply function are derived in a fashion similar to how the crop area and yield elasticities are derived.

$$\begin{matrix} Animals_{\text{j,fpu,livsys}}=AnimalInt_{\text{j,fpu,livsys}}\times AnimalInt2_{\text{j,fpu,livsys}}\times\left(\frac{PNET_{\text{j,cty}}}{PNET0_{\text{j,cty}}}\right)^{AN\varepsilon}\times\prod_{\text{cfeeds}} \left(\frac{PC_{\text{c,cty}}}{PC0_{\text{c,cty}}}\right)^{Feed\varepsilon} \\ Animals=\text{Number of producing animals} \\ AnimalInt=\text{Animal intercept (initial number of animals)} \\ AnimalInt2=\text{Exogenous population growth} \\ PC=\text{Consumer prices} \\ PC0=\text{Intial consumer prices} \\ Feed\varepsilon=\text{Supply elasticity with respect to changes in feed prices} \\ livsys=\text{Livestock production systems} \\ cfeeds=\text{Feed commodities demanded by livestock sector} \end{matrix}$$

Livestock yields are determined through exogenous growth due to improved animals and management practices. Currently, all price responses in the livestock sector are accounted for in the animal number equations.

$$\begin{matrix} AnimalYield_{\text{j,fpu,livsys}}=AnimalYieldInt_{\text{j,fpu,livsys}}\times AnimalYieldInt2_{\text{j,fpu,livsys}} \\ AnimalYield=\text{Animal yields} \\ AnimalYieldInt=\text{Initial animal yields} \\ AnimalYieldInt2=\text{Exogenous yield growth} \end{matrix}$$

Total national production (*QS*_j,cty_) is calculated by multiplying the number of slaughtered animals by the yield per head and summing across FPU and livestock system.

$$QS_{\text{j,cty}}=\sum_{\text{fpu,livsys}} \left(Animals_{\text{j,fpu,livsys}}\times AnimalYield_{\text{j,fpu,livsys}}\right)$$

## Production of Processed Goods

Modeling of processed goods is handled through the implementation of an activity-commodity framework, which allows for a general handling of all processed goods in IMPACT through input-output matrixes and the use of net prices. The input-output matrixes represent technical coefficients on input requirements, are specified by quantities of inputs per unit of output (i.e. metric tons of soybeans per metric tons of soybean oil), and are calculated from the base data. The net price is the price the producer receives net of input costs. The net price will equal the producer price of the activity whenever there are no intermediate inputs.[[3]](#footnote-3)

$$\begin{matrix} PNET_{\text{j,cty}}=PP_{\text{j,cty}}-\sum_{\text{inputs}} \left(IOMAT_{\text{inputs,j,cty}}\times\left(1-CSEI_{\text{inputs,cty}}\right)\times PC_{\text{inputs,cty}}\right) \\ PNET=\text{Net price} \\ PP=\text{Producer price} \\ PC=\text{Consumer price of inputs} \\ CSEI=\text{Consumer support estimate on intermediate inputs} \\ IOMAT=\text{Input-output matrix} \\ inputs=\text{Set of commodities (c) that are inputs into activity j} \end{matrix}$$

Production of processed goods is then simulated by a supply function that incorporates both endogenous price effects and exogenous technological change. As opposed to crop and livestock production, processed goods are modeled at the country level instead of at the FPU.

$$\begin{matrix} QS_{\text{j,cty}}=QSInt_{\text{j,cty}}\times QSINT2_{\text{j,cty}}\times\left(\frac{PNET_{\text{j,cty}}}{PNET0_{\text{j,cty}}}\right)^{QS\varepsilon} \\ QS=\text{Total production} \\ QSInt=\text{Initial production} \\ QSInt2=\text{Exogenous productivity growth} \\ QS\varepsilon=\text{Supply elasticity with respect to net price} \end{matrix}$$

## Commodity Supply and Demand

Total supply of commodities requires mapping from output of production activities to supply of commodities. The mapping is given by the following:

$$\begin{matrix} QSUP_{\text{c,cty}}=\sum_{\text{j}} JCRatio_{\text{j,c}}\times QS_{\text{j,cty}} \\ QSUP=\text{Total commodity supply} \\ JCRatio=\text{Activity to commodity mapping} \\ c=\text{Commodity} \\ cty=\text{Country} \end{matrix}$$

The parameter *JCRatio* maps from the activity output to commodities. Usually, each activity produces a matched commodity (e.g. wheat-growing activity produces the commodity wheat and nothing else). The specification, however, is general. There can be many activities producing the same commodity (e.g. different rice-growing activities producing the same rice commodity) or a single activity producing more than one commodity (e.g. oil seed processing yielding both oil and meal). By convention, the units of j agree with the units of the main commodity produced by the activity (e.g. output of the wheat activity yields the commodity wheat, in the same units), so that the *JCRatio* for this mapped commodity always equals 1. Other outputs, if any, from an activity in *JCRatio* are measured as ratios to the output of the main activity (e.g. tons of meal per ton of production of oil in an oilseed-processing activity).

Total domestic demand for a commodity is the sum of household food demand, agricultural intermediate demand (feed and processed goods), and intermediate demand from other sectors (i.e. for biofuels and industrial uses).

$$\begin{matrix} QD_{\text{c,cty}}=\sum_{\text{h}} \left(QH_{\text{c,h,cty}}\right)+QInterm_{\text{c,cty}}+QL_{\text{c,cty}}+QBF_{\text{c,cty}}+QOTH_{\text{c,cty}} \\ QD=\text{Total commodity demand} \\ QH=\text{Household food demand} \\ QInterm=\text{Intermediate demand from Ag-processing sector} \\ QL=\text{Feed demand from livestock sector} \\ QBF=\text{Intermediate demand for biofuel feedstock} \\ QOth=\text{All other demand} \\ h=\text{Household type} \end{matrix}$$

Food demand is a function of the price of the commodity and the prices of other competing commodities, per capita income, and total population. Per capita income and population increase annually according to country-specific population and income growth rates. Population and gross domestic product (GDP) trends vary by scenario. The IMPACT demand elasticities are originally based on United States Department of Agriculture–estimated elasticities and adjusted to represent a synthesis of average, aggregate elasticities for each region, given the income level and distribution of urban and rural populations (United States Department of Agriculture 1998). Trends in the elasticities adjust to accommodate the gradual shift in demand from staples to high-value commodities like meat, especially in developing countries. This assumption is based on expected economic growth, increased urbanization, and continued commercialization of the agricultural sector. IMPACT currently treats household demand with one representative consumer per country.

Feed demand is a derived intermediate demand. It is determined by two components: (1) animal feed requirements determined by livestock production and livestock feed requirements and (2) price effects that take into account potential substitution possibilities among different feeds. The equation also incorporates a technology parameter that indicates improvements in feeding efficiencies over time.

$$\begin{matrix} QL_{\text{c,cty}}=\sum_{\text{jlvst}} \left(QS_{\text{jlvst,cty}}\times\mathrm{Re}q_{\text{jlvst,c,cty}}\right)\times\prod_{\text{cfeeds}} \left(\frac{PC_{\text{c,cty}}}{PC0_{\text{c,cty}}}\right)^{LFD\varepsilon} \\ QL=\text{Total feed demand for livestock sector} \\ QS=\text{Total production of each livestock activity} \\ \mathrm{Re}q=\text{Feed requirements for each livestock activity} \\ LFD\varepsilon=\text{Price elasticity of demand for feed} \\ jlvst=\text{Set of livestock producing activities} \end{matrix}$$

Intermediate demand is a derived demand based on the demand for final processed goods, such as vegetable oils and sugar. The input-output matrix determines the proportions of inputs (c) required for each producing activity (j).

$$\begin{matrix} QDInterm_{\text{c,cty}}=\sum_{\text{j}} \left(IOMat_{\text{c,j,cty}}\times QS_{\text{j,cty}}\right) \\ QDInterm=\text{Intermediate demand} \\ IOMat=\text{Input-Output matrix} \end{matrix}$$

Biofuel feedstock demand is determined through exogenous growth rates, which represent government mandates to encourage the production of biofuels though adjusted where the mandates are, according to expert judgement, infeasible or to reflect the roles of first- or second-generation biofuels. The biofuel feedstock demand equation also allows for a price response for biofuels to allow for substitution across different potential feedstocks as well as to reflect the reality that increasing food prices would likely lead to easing biofuel of mandates.

$$\begin{matrix} QBF_{\text{c,cty}}=QBFInt_{\text{c,cty}}\times QBFINT2_{\text{c,cty}}\times\prod_{\text{c}} \left(\frac{PC_{\text{c,cty}}}{PC0_{\text{c,cty}}}\right)^{BF\varepsilon} \\ QBF=\text{Biofuel feedstock demand} \\ QBFInt=\text{Initial demand from biofuel sector} \\ QBFInt2=\text{Exogenous growth in demand from biofuels} \\ BF\varepsilon=\text{Price elasticity of demand for biofuel feedstock} \end{matrix}$$

Other demand summarizes all other demands for agricultural products from sectors outside of the focus of IMPACT (e.g. seeds, industrial use). It is simulated under two equations. The primary method follows the household food demand equation and is sensitive to changes in income, population, and prices.

$$\begin{matrix} QOth_{\text{c,cty}}=QOthInt_{\text{c,cty}}\times\left(\frac{pcGDP_{\text{cty}}}{pcGDP0_{\text{cty}}}\right)^{IOth\varepsilon}\times\left(\frac{POP_{\text{cty}}}{POP0_{\text{cty}}}\right)\times\prod_{cc} \left(\frac{PC_{\text{c,cty}}}{PC0_{\text{c,cty}}}\right)^{POth\varepsilon} \\ QOth=\text{Other Demand} \\ QOthInt=\text{Initial other demand} \\ IOth\varepsilon=\text{Income demand elasticity for other demand} \\ POth\varepsilon=\text{Price demand elasticity for other demand} \end{matrix}$$

The second method is used in a few cases where other demand historically has not shown much of a response to prices and is instead a function of changes in per capita GDP from the previous year (*pcGDP*1).

$$\begin{matrix} QOth_{\text{c,cty}}=QOth1_{\text{c,cty}}\times\frac{pcGDP_{\text{cty}}}{pcGDP1_{\text{cty}}} \\ QOth=\text{Other demand} \\ QOth1=\text{Lagged other demand} \\ pcGDP1=\text{Lagged per capita GDP} \end{matrix}$$

## Markets, Trade, and Equilibrium Prices

The system of equations is written in the GAMS programming language (GAMS 2017). The solution of these equations is achieved by the Path solver. This procedure finds a set of domestic and world prices for all crops that clear domestic and international commodity markets. The world price of a commodity is the equilibrating mechanism for traded commodities—when an exogenous shock is introduced in the model, world price will adjust to clear world markets, and each adjustment is passed back to the effective producer and consumer prices via the price transmission equations. Changes in domestic prices subsequently affect commodity supply and demand, necessitating their iterative readjustments until world supply and demand balance and world net trade again equals 0. For nontraded commodities, domestic prices in each country adjust to equate supply and demand within the country.

IMPACT assumes a closed world economy—at the end of every year the world’s production must equal the world’s demand. This constraint is ensured by the following equation, where the sum of net trade over the globe must equal 0.

$$\begin{matrix} \sum_{\text{cty}} NT_{\text{c,cty}}=0 \\ NT=\text{Net Trade} \end{matrix}$$

National production and demand for tradable commodities are linked to world markets through trade. Commodity trade by country (cty) is a function of domestic production, domestic demand, and stock change.[[4]](#footnote-4) Regions with positive net trade are net exporters, while those with negative values are net importers. This specification does not permit a separate identification of international bilateral trade by country of origin and destination—all countries export to and import from a single global market.

$$\begin{matrix} NT_{\text{c,cty}}=QSUP_{\text{c,cty}}-QD_{\text{c,cty}}-QSt_{\text{c,cty}} \\ NT=\text{Net trade} \\ QSt=\text{Change in stocks} \end{matrix}$$

Prices are endogenous in the system of equations for food and are calibrated to 2005 commodity prices (OECD Agricultural Market Access Database 2010). Prices are in constant 2005 US dollars. Domestic prices of tradable commodities are a function of world prices, adjusted by the effect of trade policy represented by taxes and tariffs, with price policies expressed in terms of producer support estimates (PSEs), consumer support estimates (CSEs), and the cost of moving products from one market to another represented by marketing margins (MMs). Export taxes and import tariffs are drawn from data from the Global Trade Analysis Project database at Purdue University and reflect trade policies at the national level (Narayanan and Walmsley 2008; International Trade Center 2006; Boumellassa et al., 2009). PSEs and CSEs represent public policies to support production and consumption by creating wedges between world and domestic prices. PSEs and CSEs are based on Organisation for Economic Co-operation and Development (OECD) estimates and are adjusted by expert judgment to reflect regional trade dynamics (OECD 2014). MMs reflect other factors such as transport and marketing costs of getting goods to various markets and are based on expert opinion on the quality and availability of transportation, communication, and market infrastructure.

In the model, PSEs, CSEs, and MMs are expressed as percentages (ad valorem) of the world price. To calculate producer prices the appropriate wedges are applied to the domestic consumer prices (*PC*) and represent the markup observed in domestic markets from the farm-- or factory-gate prices producers receive. The producer price of an activity is the weighted sum of the prices of the commodities associated with that activity.

$$\begin{matrix} PP_{\text{j,cty}}\times\left(1+MMJ_{\text{j,cty}}\right)=\left(1+PSE_{\text{j,cty}}\right)\times\sum_{\text{c}} JCRatio_{\text{j,c,cty}}\times PC_{\text{c,cty}} \\ PP=\text{Producer price} \\ MMJ=\text{Farm(factory)-gate to domestic market Marketing Margin (MM)} \\ PSE=\text{Producer support estimate, ad valorem component} \\ JCRatio\text{ = mapping from activities (j) to commodities (c)} \end{matrix}$$

Commodities can be specified as either tradable or non-tradable. Traded commodity prices are determined in international markets. Non-traded commodities are those commodities whose prices are determined in national markets, without direct links to international markets. An example is sugarcane, where all demand is treated as intermediate demand from the domestic sugar processing sector. These commodity prices are determined endogenously by country and ensure domestic supply and demand equal.

$$QSUP_{\text{c,cty}}=QD_{\text{c,cty}}$$

Nontraded commodities are indirectly linked to world markets through the demand for final products (i.e. sugar).

Table S1 Regional Aggregations

| IMPACT Country | | Aggregations | | |
| --- | --- | --- | --- | --- |
| Code | Name | Economic | Global Regions | Africa Regions |
| AFG | Afghanistan | Developing | Asia |  |
| AGO | Angola | Developing | Africa | Middle Africa |
| ALB | Albania | Developing | Europe |  |
| ARG | Argentina | Developing | Americas |  |
| ARM | Armenia | Developing | Asia |  |
| AUS | Australia | Developed | Oceania |  |
| AUT | Austria | Developed | Europe |  |
| AZE | Azerbaijan | Developing | Asia |  |
| BDI | Burundi | Developing | Africa | Eastern Africa |
| BEN | Benin | Developing | Africa | Western Africa |
| BFA | Burkina Faso | Developing | Africa | Western Africa |
| BGD | Bangladesh | Developing | Asia |  |
| BGR | Bulgaria | Developing | Europe |  |
| BLR | Belarus | Developing | Europe |  |
| BLT | Baltic States | Developed | Europe |  |
| BLX | Belgium-Luxembourg | Developed | Europe |  |
| BLZ | Belize | Developing | Americas |  |
| BOL | Bolivia | Developing | Americas |  |
| BRA | Brazil | Developing | Americas |  |
| BTN | Bhutan | Developing | Asia |  |
| BWA | Botswana | Developing | Africa | Southern Africa |
| CAF | Central African Rep. | Developing | Africa | Middle Africa |
| CAN | Canada | Developed | Americas |  |
| CHL | Chile | Developing | Americas |  |
| CHM | China | Developing | Asia |  |
| CHP | Switzerland | Developed | Europe |  |
| CIV | Ivory Coast | Developing | Africa | Western Africa |
| CMR | Cameroon | Developing | Africa | Middle Africa |
| COD | DRC | Developing | Africa | Middle Africa |
| COG | Congo | Developing | Africa | Middle Africa |
| COL | Colombia | Developing | Americas |  |
| CRB | Other Caribbean | Developing | Americas |  |
| CRI | Costa Rica | Developing | Americas |  |
| CUB | Cuba | Developing | Americas |  |
| CYP | Cyprus | Developed | Asia |  |
| CZE | Czech Republic | Developed | Europe |  |
| DEU | Germany | Developed | Europe |  |
| DJI | Djibouti | Developing | Africa | Eastern Africa |
| DNK | Denmark | Developed | Europe |  |
| DOM | Dominican Republic | Developing | Americas |  |
| DZA | Algeria | Developing | Africa | Northern Africa |
| ECU | Ecuador | Developing | Americas |  |
| EGY | Egypt | Developing | Africa | Northern Africa |
| ERI | Eritrea | Developing | Africa | Eastern Africa |
| ETH | Ethiopia | Developing | Africa | Eastern Africa |
| FJI | Fiji | Developing | Oceania |  |
| FNP | Finland | Developed | Europe |  |
| FRP | France | Developed | Europe |  |
| GAB | Gabon | Developing | Africa | Middle Africa |
| GEO | Georgia | Developing | Asia |  |
| GHA | Ghana | Developing | Africa | Western Africa |
| GIN | Guinea | Developing | Africa | Western Africa |
| GMB | Gambia | Developing | Africa | Western Africa |
| GNB | Guinea-Bissau | Developing | Africa | Western Africa |
| GNQ | Equatorial Guinea | Developing | Africa | Middle Africa |
| GRC | Greece | Developed | Europe |  |
| GRL | Greenland | Developed | Americas |  |
| GSA | Guyanas | Developing | Americas |  |
| GTM | Guatemala | Developing | Americas |  |
| HND | Honduras | Developing | Americas |  |
| HRV | Croatia | Developed | Europe |  |
| HTI | Haiti | Developing | Americas |  |
| HUN | Hungary | Developed | Europe |  |
| IDN | Indonesia | Developing | Asia |  |
| IND | India | Developing | Asia |  |
| IRL | Ireland | Developed | Europe |  |
| IRN | Iran | Developing | Asia |  |
| IRQ | Iraq | Developing | Asia |  |
| ISL | Iceland | Developed | Europe |  |
| ISR | Israel | Developed | Asia |  |
| ITP | Italy | Developed | Europe |  |
| JAM | Jamaica | Developing | Americas |  |
| JOR | Jordan | Developing | Asia |  |
| JPN | Japan | Developed | Asia |  |
| KAZ | Kazakhstan | Developing | Asia |  |
| KEN | Kenya | Developing | Africa | Eastern Africa |
| KGZ | Kyrgyzstan | Developing | Asia |  |
| KHM | Cambodia | Developing | Asia |  |
| KOR | South Korea | Developed | Asia |  |
| LAO | Laos | Developing | Asia |  |
| LBN | Lebanon | Developing | Asia |  |
| LBR | Liberia | Developing | Africa | Western Africa |
| LBY | Libya | Developing | Africa | Northern Africa |
| LKA | Sri Lanka | Developing | Asia |  |
| LSO | Lesotho | Developing | Africa | Southern Africa |
| MDA | Moldova | Developing | Europe |  |
| MDG | Madagascar | Developing | Africa | Eastern Africa |
| MEX | Mexico | Developing | Americas |  |
| MLI | Mali | Developing | Africa | Western Africa |
| MMR | Myanmar | Developing | Asia |  |
| MNG | Mongolia | Developing | Asia |  |
| MOR | Morocco | Developing | Africa | Northern Africa |
| MOZ | Mozambique | Developing | Africa | Eastern Africa |
| MRT | Mauritania | Developing | Africa | Western Africa |
| MWI | Malawi | Developing | Africa | Eastern Africa |
| MYS | Malaysia | Developing | Asia |  |
| NAM | Namibia | Developing | Africa | Southern Africa |
| NER | Niger | Developing | Africa | Western Africa |
| NGA | Nigeria | Developing | Africa | Western Africa |
| NIC | Nicaragua | Developing | Americas |  |
| NLD | Netherlands | Developed | Europe |  |
| NOR | Norway | Developed | Europe |  |
| NPL | Nepal | Developing | Asia |  |
| NZL | New Zealand | Developed | Oceania |  |
| OAO | Other Atlantic | Developing | Europe |  |
| OBN | Other Balkans | Developed | Europe |  |
| OIO | Other Indian Ocean | Developing | Asia |  |
| OPO | Other Pacific Ocean | Developing | Oceania |  |
| OSA | Other Southeast Asia | Developing | Asia |  |
| PAK | Pakistan | Developing | Asia |  |
| PAN | Panama | Developing | Americas |  |
| PER | Peru | Developing | Americas |  |
| PHL | Philippines | Developing | Asia |  |
| PNG | Papua New Guinea | Developing | Oceania |  |
| POL | Poland | Developed | Europe |  |
| PRK | North Korea | Developing | Asia |  |
| PRT | Portugal | Developed | Europe |  |
| PRY | Paraguay | Developing | Americas |  |
| PSE | Palestine | Developing | Asia |  |
| RAP | Rest of Arabia | Developed | Asia |  |
| ROU | Romania | Developing | Europe |  |
| RUS | Russia | Developing | Europe |  |
| RWA | Rwanda | Developing | Africa | Eastern Africa |
| SAU | Saudi Arabia | Developed | Asia |  |
| SDN | Sudan | Developing | Africa | Northern Africa |
| SEN | Senegal | Developing | Africa | Western Africa |
| SLB | Solomon Islands | Developing | Oceania |  |
| SLE | Sierra Leon | Developing | Africa | Western Africa |
| SLV | El Salvador | Developing | Americas |  |
| SOM | Somalia | Developing | Africa | Eastern Africa |
| SPP | Spain | Developed | Europe |  |
| SVK | Slovakia | Developed | Europe |  |
| SVN | Slovenia | Developed | Europe |  |
| SWE | Sweden | Developed | Europe |  |
| SWZ | Swaziland | Developing | Africa | Southern Africa |
| SYR | Syria | Developing | Asia |  |
| TCD | Chad | Developing | Africa | Middle Africa |
| TGO | Togo | Developing | Africa | Western Africa |
| THA | Thailand | Developing | Asia |  |
| TJK | Tajikistan | Developing | Asia |  |
| TKM | Turkmenistan | Developing | Asia |  |
| TLS | Timor L'Este | Developing | Asia |  |
| TUN | Tunisia | Developing | Africa | Northern Africa |
| TUR | Turkey | Developing | Asia |  |
| TZA | Tanzania | Developing | Africa | Eastern Africa |
| UGA | Uganda | Developing | Africa | Eastern Africa |
| UKP | UK | Developed | Europe |  |
| UKR | Ukraine | Developing | Europe |  |
| URY | Uruguay | Developing | Americas |  |
| USA | USA | Developed | Americas |  |
| UZB | Uzbekistan | Developing | Asia |  |
| VEN | Venezuela | Developing | Americas |  |
| VNM | Vietnam | Developing | Asia |  |
| VUT | Vanuatu | Developing | Oceania |  |
| YEM | Yemen | Developing | Asia |  |
| ZAF | South Africa | Developing | Africa | Southern Africa |
| ZMB | Zambia | Developing | Africa | Eastern Africa |
| ZWE | Zimbabwe | Developing | Africa | Eastern Africa |

Table S2 Commodity Aggregations

| Commodity Group | | Commodity |
| --- | --- | --- |
| Animal Products | Meat | Beef |
|  |  | Lamb |
|  |  | Pork |
|  |  | Poultry |
|  | Other | Dairy |
|  |  | Eggs |
| Crops | Cereals | Barley |
|  |  | Maize |
|  |  | Millet |
|  |  | Other Cereals |
|  |  | Rice |
|  |  | Sorghum |
|  |  | Wheat |
|  | Fruits and Vegetables | Banana |
|  |  | Plantain |
|  |  | Temperate Fruit |
|  |  | Tropical Fruit |
|  |  | Vegetables |
|  | Oilseeds | Groundnut |
|  |  | Other Oilseeds |
|  |  | Palm Fruit |
|  |  | Palm Kernel |
|  |  | Rapeseed |
|  |  | Soybean |
|  |  | Sunflower |
|  | Other Crops | Cacao |
|  |  | Coffee |
|  |  | Cotton |
|  |  | Other |
|  |  | Tea |
|  | Pulses | Beans |
|  |  | Chickpeas |
|  |  | Cowpeas |
|  |  | Lentils |
|  |  | Other Pulses |
|  |  | Pigeon peas |
|  | Root and Tubers | Cassava |
|  |  | Other Roots |
|  |  | Potato |
|  |  | Sweet Potato |
|  |  | Yams |
|  | Sugar Crops | Sugar beet |
|  |  | Sugarcane |
| Processed Commodities | Oilmeals | Groundnut meal |
|  |  | Other meals |
|  |  | Palm Kernel Meal |
|  |  | Rapeseed Meal |
|  |  | Soybean Meal |
|  |  | Soybean Oil |
|  |  | Sunflower Meal |
|  | Processed Food Oils | Groundnut Oil |
|  |  | Other Oils |
|  |  | Palm Fruit Oil |
|  |  | Palm Kernel Oil |
|  |  | Rapeseed Oil |
|  |  | Soybean Oil |
|  |  | Sunflower Oil |
|  | Other | Sugar |

# Summary Description of GLOBE-Energy

The following description is a summary of the documentation for the GLOBE-Energy model, an extended dynamic version of the comparative-static standard GLOBE model originally developed by McDonald, Thierfelder and Robinson (2007). Apart from the incorporation of capital accumulation, population growth, labor force growth and technical progress, the extended model features a stylized representation of the technical substitution possibilities among different energy sources in production using a state-of the-art KLEM (Capital (**K**), **Labor**, **E**nergy, **M**aterials) technology specification.

GLOBE is a multi-country computable general equilibrium (CGE) model that consists of a set of individual country or region blocs that together provide complete coverage of the global economy and that are linked through international trade and capital flows. The modeling system solves the within country models and between country trade relationships simultaneously to ensure full global consistency among all variables. Each region bloc represents the whole economy of that region at a sectorally disaggregated level. The economic interactions among producers, consumers and the government, as well as economic transactions with other regions, are explicitly captured. Producers in each region combine primary factors (that is, skilled and unskilled labor, physical capital, land, and other natural resources) and intermediate inputs obtained from the same and other production sectors at home and abroad to produce output. The output is sold to domestic households, the domestic government, to domestic producers (for use as intermediate input or as an addition to the productive capital stock) and to the rest of the world.

The production process generates factor income in the form of wages, other in-kind returns to labor, land and natural resource rents, and returns to capital as well as production tax income for the government. The factor income flows to households. Households use their income to pay income taxes, to buy consumer goods, and to save for future consumption. The government receives additional tax revenue from sales taxes including revenue from import duties.

The model parameters governing household, producer, and government decisions are set in line with observed data for the reference year 2007, so that the model equilibrium, in the absence of policy changes or other exogenous shocks, exactly replicates the reference year data.

The model is initially calibrated to the GTAP 8 database (Narayanan, Aguiar, & McDougall, 2012) that combines detailed bilateral trade and protection data reflecting economic linkages among regions with individual country input-output data, which account for intersectoral linkages within regions, for the benchmark year 2007.

## Production, Input Demand, and Factor Markets

Production relationships by activity are characterized by constant returns to scale and specified by nested Constant Elasticity of Substitution (CES) production functions. In the standard version, activity output is a CES composite of an aggregate intermediate input and aggregate value added, where the aggregate intermediate input is a Leontief aggregate of the individual intermediate commodity inputs and aggregate value added is a CES composite of primary factors. The determination of product supply and input demand is based on the assumption of profit maximizing behavior.

For each region bloc, the model allows adoption of either a standard neoclassical factor market closure or a closure with labor underemployment. Under the former, factor markets in all regions are characterized by inelastic factor supplies and the model solves for market-clearing factor prices. The primary factors, except sector-specific natural resource endowments, are mobile across production activities but immobile across borders. Under the latter closure option, the wage for unskilled labor is fixed relative to the domestic consumer price index and the supply of unskilled labor is perfectly elastic.

## Energy Production and Intermediate Use in the Extended Model

In energy-focused CGE modelling, technology specifications belonging to the generic class of KLEM (Capital (**K**), **Labor**, **E**nergy, **M**aterials) production functions are commonly employed to capture substitution possibilities among energy and non-energy inputs and among different energy sources. GLOBE-Energy follows this established standard approach. The sectoral KLEM production functions for activities selected by the user take the form of nested multi-level functions with a (positive or zero) constant elasticity of substitution (CES) among inputs grouped together within the same nest. Figure S1 displays the input nesting hierarchy.

Figure S1 Production Function Nesting Structure

In each sector, the production of a given output quantity requires non-energy inputs and a value-added/energy composite in fixed or variable proportions. For the few sectors that use crude oil directly as an input (i.e. the refined fuels industry and the chemical industry), crude oil inputs are a fixed proportion of output. The value added/energy composite requires energy and primary factors in variable proportions. Thus, when the price index of energy rises relative to primary factor prices, energy inputs are replaced to some extent by additional inputs of capital and labor.

Required energy inputs are composed of electricity purchases from the electricity sector in the model and direct use of fossil fuels. The model allows substitution of these primary fossil energy for electricity in sectors where the input-output matrices of the GTAP database record intermediate purchases of fossil fuels. At the bottom of the input substitution hierarchy, the sectoral production functions allow for imperfect substitutability between coal, refined oil, and natural gas.

The ease of substitution between energy and non-energy inputs, and among energy inputs, is governed by elasticities of substitution for the various nests. At the top level, the default option is perfect complementarity between the value-added-energy composite and material non-energy inputs. For the crucial elasticities of substitution between value added and the energy composite, the recent empirical evidence suggests plausible values well below unity.[[5]](#footnote-5) For the bottom-level fossil fuel nest, the default option is a unitary inter-fuel substitution elasticity, which is broadly in line with the results of the meta-analysis by Stern (2012) covering 47 individual empirical studies.

## Final Domestic Demand by Commodity

The commodity composition of government consumption demand and investment demand is fixed using the observed demand patterns from the benchmark data set, while the determination of the aggregate levels for these final demand components in each region depends on the choice of macro closure, as explained in following sections. Households are utility maximizers who respond to changes in relative prices and disposable incomes. In this version of the model, the utility functions for private households take the Stone-Geary form and, hence, consumer demand by commodity is described by a Linear Expenditure System (LES) specification.

## International Trade

Domestically produced commodities are assumed to be imperfect substitutes for traded goods. Import demand is modelled via a series of nested constant elasticity of substitution (CES) functions; imported commodities from different source regions to a destination region are assumed to be imperfect substitutes for each other and are aggregated to form composite import commodities that are assumed to be imperfect substitutes for their counterpart domestic commodities. The composite imported commodities and their counterpart domestic commodities are then combined to produce composite consumption commodities, which are the commodities demanded by domestic agents as intermediate inputs and final demand (private consumption, government, and investment). Export supply is modeled via a series of nested constant elasticity of transformation (CET) functions; the composite export commodities are assumed to be imperfect substitutes for domestically consumed commodities, while the exported commodities from a source region to different destination regions are assumed to be imperfect substitutes for each other. The composite exported commodities and their counterpart domestic commodities are then combined as composite production commodities. The use of nested CET functions for export supply implies that domestic producers adjust their export supply decisions in response to changes in the relative prices of exports and domestic commodities. This specification is desirable in a global model with a mix of developing and developed countries that produce different kinds of traded goods with the same aggregate commodity classification and yields more realistic behavior of international prices than models assuming perfect substitution on the export side.

## Macro Closure

Current account balances for all regions are assumed to be fixed at initial benchmark levels in terms of a global numeraire and real exchange rates adjust to maintain external equilibrium. Under the default macro-closure, changes in aggregate absorption are assumed to be shared equally (to maintain the shares from the base data) among private consumption, government, and investment demands. Household and government saving rates adjust residually to establish the macroeconomic saving-investment balance in each region.

## The Algebra of GLOBE

### Exports

1. PEDEF Composite export price for commodity c of origin r

$${PE}_{c,r}={\sum_{w} \left( {PER}_{c,w,r}* {QER}_{c,w,r} \right)}/{{QE}_{c,r}} , c \in ce, r \in rgn$$

2. PERDEF2 Domestic price of exports of commodity c of origin r to region w

$${PER}_{c,w,r}= {PWE}_{c,w,r} * \left( 1 - {TE}_{c,w,r} \right) * {ER}_{r} , c \in cer$$

3. CET Upper-level CET function (QXC = CET(QE,QD)

${QXC}_{c,r}= {at}_{c,r}* \left( \gamma_{c,r}* \left( {QE}_{c,r} \right)^{\rho_{c,r}^{t}} + \left( 1-\gamma_{c,r} \right) * \left( {QD}_{c,r} \right)^{\rho_{c,r}^{t}} \right)^{1/{\rho_{c,r}^{t}}}$, $c \in\left( cd \cap ce \right), r \in rgn$

1. PXCDEF Price of composite output

$${PXC}_{c,r}* {QXC}_{c,r}= \left( {PD}_{c,r} * {QD}_{c,r} \right) + \left( {PE}_{c,r} * {QE}_{c,r} \right) , c \in cx, r \in rgn$$

2. ESUPPLY Export supply (FOC)

${QE}_{c,r}= {QD}_{c,r}* \left( \left( {{PE}_{c,r}}/{{PD}_{c,r}} \right) * \left( \left( 1-\gamma_{c,r} \right)/{\gamma_{c,r}} \right) \right)^{1/\left( \rho_{c,r}^{t} -1 \right)}$*,* $c \in\left( cd \cap ce \right), r \in rgn$

1. CETLEV2 Export supply of c of origin r to region w (FOC)

${QER}_{c,w,r}= {QE}_{c,r}* \left( {{PER}_{c,w,r}}/\left( {PE}_{c,r}* \gamma_{c,w,r}^{r} * \left( {atr}_{c,r} \right)^{\rho_{c,r}^{e}} \right) \right)^{1/\left( \rho_{c,r}^{e} -1 \right)}$, $c \in cer, r \in rgn$

NB:The elasticity of transformation between exports to different destinations is ${\tau_{c,r}^{e}=1}/\left( \rho_{c,r}^{e} -1 \right)$.

1. CETALT QXC for commodities not exported by r or only produced for exports by r

${QXC}_{c,r}= {QD}_{c,r}+{QE}_{c,r} , c \in\left( cd\cap cen \right) \cup\left( cdn\cap ce \right), r\in rgn$

### Trade and Transport Margins

1. TSHIP Trade margin service exports by globe equal its trade margin imports

$${QE}_{ct2,globe}={QM}_{ct2,globe}$$

2. CETREQG Aggregate margin exports by globe

$${QE}_{ct2,globe}=\sum_{w} {QER}_{ct2,w,globe}$$

3. PERDEFHG Trade margin service export price

$${PER}_{ct2,w,globe}={PE}_{ct2,globe}$$

4. QTEQ Total ct2 margin quantity on overall imports by r of origin w

$${QT}_{w,ct2,rgn}=\sum_{c} {margcor}_{w,ct2,c,rgn}{QMR}_{w,c,rgn}$$

5. GLOBEQUIL Region r's margin demand from globe equals globe’s supply to r

$$\sum_{w} {QT}_{w,ct2,rgn}={QER}_{ct2,rgn,globe}$$

6. PTDEF Price of trade margin commodity

$${PT}_{ct2,rgn}={PWE}_{ct2,rgn,globe}$$

7. KAPREQUIL2 Region r’s net margin service imports from globe

$${KAPREG}_{globe,r}= \sum_{ct2} \sum_{w} {PT}_{ct2,r}{QT}_{w,ct2,r}-\sum_{ct2} {PWE}_{ct2,globe,r}{QER}_{ct2,globe,r}$$

8. ARMINGTON Upper-Level Armington aggregator

${QQ}_{c,r}= {ac}_{c,r}* \left( \delta_{c,r}* \left( {QM}_{c,r} \right)^{{-\rho}_{c,r}^{c}} + \left( 1-\delta_{c,r} \right) * \left( {QD}_{c,r} \right)^{{-\rho}_{c,r}^{c}} \right)^{{-1}/{\rho_{c,r}^{c}}}$, $c \in\left( cx\cap cm \right), r \in rgn$

N.B: The elasticity of substitution between composite imports and the domestic substitute (Armington elasticity) is $\sigma_{c,r}^{c}=1/{(1+\rho_{c,r}^{c}}).$

1. COSTMIN Optimal import ratio (FOC)

${QM}_{c,r}= {QD}_{c,r}* \left( \left( {{PD}_{c,r}}/{{PM}_{c,r}} \right)* \left( {\delta_{c,r}}/\left( 1-\delta_{c,r} \right) \right) \right)^{1/{(1+\rho_{c,r}^{c})}}$, $c \in\left( cx\cap cm \right), r \in rgn$

1. PQSDEF Supply price of Armington composite

$${PQS}_{c,r}*{QQ}_{c,r}=\left( {PD}_{c,r}*{QD}_{c,r} \right)+\left( {PM}_{c,r}*{QM}_{c,r} \right) , c\in\left( cd\cup cm \right), r\in rgn$$

2. PQDDEF User price of Armington composite

$${PQD}_{c,r}= {PQS}_{c,r} * \left( 1 + {TS}_{c,r} \right) , c \in\left( cd \cup cm \right), r \in rgn$$

3. ARMLEV2 Import demand for c by r of origin w

$${QMR}_{w,c,r}= {QML}_{c,r}* \left( \left( {{PMR}_{w,c,r}* \left( {acr}_{c,r} \right)^{\rho_{c,r}^{m}}}/{{PML}_{c,r}}* \delta_{w,c,r}^{r} \right) \right)^{{-1}/{1+\rho_{c,r}^{m}}} , c \in cmrl$$

NB:The elasticity of substitution between imports from different origins is $\sigma_{c,r}^{m}=1/{(1+\rho_{c,r}^{m})}$ .

1. PMDEF Composite price of imports of c by region r

$${PM}_{c,r}* {QM}_{c,r}= \left( {PML}_{c,r} * {QML}_{c,r} \right) + \left( {PMS}_{c,r} * {QMS}_{c,r} \right) , c \in cm$$

2. PMLDEF Composite price of large-share imports of c by region r

$${PML}_{c,r}={\sum_{w\in cmrl\left( w,c,r \right)} \left( {PMR}_{w,c,r}* {QMR}_{w,c,r} \right)}/{{QML}_{c,r}} , c \in cml(c,r)$$

NB: The summation is over origin regions with a market share > *smimpsh* in region r’s total import bill for commodity c. Here *smimpsh* is a user-defined cut-off point to separate large and small share import flows.

1. QMLEQ Total large-share imports of c by r

$${QML}_{c,r}= {ioqmlqm}_{c,r} * {QM}_{c,r} , c \in cml$$

2. QMSEQ Total small-share imports of c by r

$${QMS}_{c,r}= {ioqmsqm}_{c,r} * {QM}_{c,r} , c \in cms$$

3. QMREQ Demand for imports with a tiny share in r’s total imports of c

$${QMR}_{w,c,r}= {ioqmrqms}_{w,c,r} * {QMS}_{c,r} , c \in cmrs(w,c,r)$$

4. PMSDEF Aggregate price of imports with a tiny share in r’s total imports of c

$${PMS}_{c,r}=\sum_{w} {ioqmrqms}_{w,c,r}* {PMR}_{w,c,r} , c \in cms(c,r)$$

5. PWMDEF CIF price of imports by r of origin w

$${PWM}_{w,c,r}= {PWMFOB}_{w,c,r} +\sum_{cp\in ct2} {margcor}_{w,cp,c,r}* {PT}_{cp,r} , c\in cmr(w,c,r)$$

6. PMRDEF2 Domestic price of imports by r of origin w

${PMR}_{w,c,r}= {PWM}_{w,c,r} \cdot\left( 1 + {TM}_{w,c,r} \right) \cdot{ER}_{r} , c \in cmr$(w,c,r)

1. TRCONP PWMFOB equals PWE plus iceberg ntb wedge

${PWMFOB}_{w,c,r}= {PWE}_{c,w,r}\cdot\left( 1+{ntb}_{w,c,r} \right) ,$

$c \in cmr$

1. TRCONQ QMR – QER correspondence in presence of iceberg transport cost

$${QMR}_{w,c,r}\cdot\left( 1+{ntb}_{w,c,r} \right)= {QER}_{c,w,r} , c \in cmr$$

2. ARMALT QQ for commodities not imported or not produced by r

$${QQ}_{c,r}={QD}_{c,r}+{QM}_{c,r} , c\in(cx\cap cmn)\cup(cxn\cap cm)$$

### Production Block

QINTDEF Demand for composite intermediate input (Leontief technology case)

${QINT}_{a,r}={ioqintqx}_{a,r} * {QX}_{a,r}$

$a \in aqxn, r \in rgn$

1. QVADEF Real value added (Leontief technology case)

${QVA}_{a,r}={ioqvaqx}_{a,r} * {QX}_{a,r}$

$a \in aqxn, r \in rgn$

1. QXPRODFN Gross output production function when VA and QINT are substitutes

$${QX}_{a,r}={ADX}_{a,r} \left( \left( \delta_{a,r}^{x}* {QVA}_{a,r} \right)^{{-\rho}_{a,r}^{x}}+ \left( \left( 1-\delta_{a,r}^{x} \right) *{QINT}_{a,r} \right)^{{-\rho}_{a,r}^{x}} \right)^{{-1}/{\rho_{a,r}^{x}}}, a \in aqx, r \in rgn$$

2. QXFOC Optimal QVA-QINT ratio when VA and QINT are substitutes

${QVA}_{a,r}={QINT}_{a,r} {*\left( {{PINT}_{a,r}}/{{PVA}_{a,r}} \right)*\left( {\delta_{a,r}^{x}}/\left( 1-\delta_{a,r}^{x} \right) \right)}^{1/\left( 1+\rho_{a,r}^{x} \right)}$ , $a \in aqx, r \in rgn$

NB: The elasticity of substitution between value added and the composite intermediate input is ${\sigma_{a, r}^{x} =1}/\left( 1+\rho_{a,r}^{x} \right)$.

1. PINTDEF Price of intermediate input composite

$${PINT}_{a,r}=\sum_{c} \left( {ioqint}_{c,a,r}* {PQD}_{c,r} \right) , r \in rgn$$

2. QINTDEQ Total intermediate input demand for commodity c

$${QINTD}_{c,r} = \sum_{a} {ioqint}_{c,a,r}*{QINT}_{a,r} , c \in cintd, r \in rgn$$

3. QVAPRODFN Value-added production function

$${QVA}_{a,r}= {ADVA}_{a,r}* \left( \sum_{f} {\delta_{f,a,r}^{va} *\left( {ADFD}_{f,a,r}*{FD}_{f,a,r} \right)}^{-\rho_{a,r}^{va}} \right)^{{-1}/{\rho_{a,r}^{va}}}, r\in rgn$$

NB: The elasticity of substitution between production factors is ${\sigma_{a, r}^{va} =1}/\left( 1+\rho_{a,r}^{va} \right)$.

1. QVAFOC Optimal factor demand

$${WF}_{f,r}*{WFDIST}_{f,a,r}* \left( 1+{TF}_{f,a,r} \right) = \frac{{PVA}_{a,r} * {QVA}_{a,r} * \left( \delta_{f,a,r}^{va} * {ADFD}_{f,a,r} \right)^{-\rho_{a,r}^{va}} * \left( {FD}_{f,a,r} \right)^{-\rho_{a,r}^{va}-1}}{\sum_{fp} \delta_{fp,a,r}^{va} * \left( {ADFD}_{f,a,r}* {FD}_{fp,a,r} \right)^{-\rho_{a,r}^{va}}}$$

$$r \in rgn$$

2. PVADEF Value added price determination

$${PX}_{a,r} * \left( 1 - {TX}_{a,r} \right)* {QX}_{a,r}=\left( {PVA}_{a,r} * {QVA}_{a,r} \right) + \left( {PINT}_{a,r} * {QINT}_{a,r} \right) , r \in rgn$$

3. PXDEF Composite price of output by activity in region r

$${PX}_{a,r} = \sum_{c} {ioqxcqx}_{a,c,r}*{PXC}_{c,r} , r \in rgn$$

4. COMOUT Activity – commodity output correspondence

$${QXC}_{c,r} = \sum_{a} {ioqxcqx}_{a,c,r}*{QX}_{a,r} , r \in rgn$$

5. ADVAEQ Total factor productivity

$${ADVA}_{a,r}= {advab}_{a,r}{ADVASHFT}_{a,r} , a\in acx, r\in rgn$$

6. ADFDEQ Factor-augmenting technical progress

$${ADFD}_{f,a,r}={adfdb}_{f,a,r}{ADFDSHFT}_{f,r} , a\in acx, r\in rgn$$

7. ADXEQ

$${ADX}_{a,r}={adxb}_{a,r} , a\in aqx , r\in rgn$$

8. QVAEPRDFN1 Production function for value-added-energy composite

$${QVAE}_{a,r}={zvae}_{a,r}\left( {dva}_{a,r}{QVA}_{a,r}^{{-\rho vae}_{a}}+{dene}_{a,r}{QENE}_{a,r}^{{-\rho vae}_{a}} \right)^{-\frac{1}{{\rho vae}_{a}}} ,$$

$$a\in aene, r\in rgn$$

9. QVAEPRDFN2 Dummy QVAE for activities without KLEM technology

$${QVAE}_{a,r}={QVA}_{a,r} , a\neg\in aene , r\in rgn$$

10. QENEPRDFN Energy composite

$${QENE}_{a,r}={zene}_{a,r}\left( {dele}_{a,r}{QELE}_{a,r}^{-{\rho ene}_{a}}+{dff}_{a,r}{QFF}_{a,r}^{{-\rho ene}_{a}} \right)^{-1/{\rho ene}_{a}} , a\in aene , r\in rgn$$

11. QFFPRDFN Fossil fuels composite

$${QFF}_{a,r}={zff}_{a,r}\prod_{cff} {QFFC}_{cff,a,r}^{{dffc}_{cff,a,r}} , a\in aene , r\in rgn$$

12. QVAEEQ Optimal VA-energy ratio

$${QENE}_{a,r}=\left( \frac{{dene}_{a,r}}{{dva}_{a,r}}\frac{{PVA}_{a,r}}{{PENE}_{a,r}} \right)^{1/(1+{\rho vae}_{a})}{QVA}_{a,r} , a\in aene , r\in rgn$$

13. QENEEQ Optimal ratio of electricity to direct fossil fuel use

$${QELE}_{a,r}=\left( \frac{{dff}_{a,r}}{{dele}_{a,r}}\frac{{PQD}_{cele,r}}{{PFF}_{a,r}} \right)^{1/(1+{\rho ene}_{a})}{QFF}_{a,r} , a\in aene , r\in rgn$$

14. QFFCEQ Optimal fossil fuel use by type

$${QFFC}_{cff,a,r}=\frac{{dffc}_{cff,a,r}{PFF}_{a,r}{QFF}_{a,r}}{{PQD}_{cff,r}} , a\in aene , r\in rgn$$

15. PENEEQ Composite energy price

${PENE}_{a,r}{QENE}_{a,r}={PFF}_{a,r}{QFF}_{a,r}+{PQD}_{cele,r}{QELE}_{a,r} , a\in aene , r\in rgn$

1. PVAEEQ1 Price of value-added-energy composite

$${PVAE}_{a,r}{QVAE}_{a,r}={PVA}_{a,r}{QVA}_{a,r}+{PENE}_{a,r}{QENE}_{a,r} , a\in aene , r\in rgn$$

2. PVAEEQ2 Dummy PVAE for activities without KLEM technology

$${PVAE}_{a,r}={PVA}_{a,r} , a\neg\in aene , r\in rgn$$

3. QFFLEON Intermediate demand for fossil fuel composite (Leontief option – eneflag = 0)

$${QFF}_{a,r}={ioff}_{a,r}{QENE}_{a,r} , a\in aene , r\in rgn$$

4. QELELEON Intermediate demand for electricity (Leontief option – eneflag = 0)

$${QELE}_{a,r}={ioele}_{a,r}{QENE}_{a,r} , a\in aene , r\in rgn$$

5. QINTDEQ1 Total non-energy intermediate input demand in r

$${QINTD}_{cnener,r}=\sum_{a} {ioqint}_{cnener,a,r}{QINT}_{a,r} , r\in rgn$$

6. QINTDEQ2 Total intermediate demand for fossil fuel type cff in r

$${QINTD}_{cff,r}=\sum_{aene} {QFFC}_{cff,aene,r}+ \sum_{a\neg\epsilon aene} {ioqint}_{cff,a,r}{QINT}_{a,r} , r\in rgn$$

7. QINTDEQ3 Total intermediate electricity demand in r

$${QINTD}_{cele,r}=\sum_{aene} {QELE}_{aene,r}+ \sum_{a\neg\epsilon aene} {ioqint}_{cele,a,r}{QINT}_{a,r} , r\in rgn$$

### Household Block

1. YFEQ Gross factor income

$${YF}_{f,r} = \sum_{a} {WF}_{f,r}*{WFDIST}_{f,a,r}* {FD}_{f,a,r} , r \in rgn$$

2. YFDISTEQ Net factor income

$${YFDIST}_{f,r} = \left( {YF}_{f,r} - \left( {deprec}_{f,r}* {YF}_{f,r} \right) \right)* \left( 1 - {TYF}_{f,r} \right) , r \in rgn$$

3. YHEQ Household income

$${YH}_{h,r} = \sum_{f} {hvash}_{h,f,r} * {YFDIST}_{f,r}+\sum_{rp} ({hhtrans}_{r,rp}*\frac{{ER}_{r}}{{ER}_{rp}}-{hhtrans}_{rp,r}) , r \in rgn$$

4. HEXPEQ Aggregate household expenditure

$${HEXP}_{h,r} = {YH}_{h,r} * \left( 1 - {TYH}_{h,r} \right) * \left( 1 - {SHH}_{h,r} \right) , r \in rgn$$

5. QCDEQ Household consumption demand by commodity

${QCD}_{c,h,r}* = {qcdconst}_{c,h,r}+ \beta_{c,h,r} *\left( {HEXP}_{h,r} - \sum_{c} \left( {qcdconst}_{c,h,r} * {PQD}_{c,r} \right) \right) /{PQD}_{c,r}$ , $r \in rgn$

### Government Block

1. MTAXEQ Import tariff revenue

$${MTAX}_{r}= \sum_{c,w} {TM}_{w,c,r}* {PWM}_{w,c,r} * {ER}_{r} * {QMR}_{w,c,r} , r \in rgn$$

2. ETAXEQ Export tax revenue

$${ETAX}_{r}= \sum_{c,w} {TE}_{c,w,r}* {PWE}_{c,w,r} * {ER}_{r} * {QER}_{c,w,r} , r \in rgn$$

3. STAXEQ Sales tax revenue

$${STAX}_{r}= \sum_{c} {TS}_{c,r}* {PQS}_{c,r} *\left( {QINTD}_{c,r} + \sum_{h} {QCD}_{c,h,r} + {QGD}_{c,r} + {QINVD}_{c,r} \right) , r \in rgn$$

4. ITAXEQ Indirect production tax revenue net of production subsidies

$${ITAX}_{r}= \sum_{a} {TX}_{a,r}* {PX}_{a,r} * {QX}_{a,r} , r \in rgn$$

5. FTAXEQ Factor use tax revenue

$${FTAX}_{r}= \sum_{f,a} {TF}_{f,a,r}* {WF}_{f,r} * {WFDIST}_{f,ar} * {FD}_{f,a,r} , r \in rgn$$

6. HTAXEQ Household income tax revenue

$${HTAX}_{r}= \sum_{h} {TYH}_{h,r}* {YH}_{h,r} , r \in rgn$$

7. FYTAXEQ Factor-specific factor income tax revenue

$${FYTAX}_{r}= \sum_{f} {TYF}_{f,r}* \left( {YF}_{f,r} - \left( {deprec}_{f,r} * {YF}_{f,r} \right) \right) , r \in rgn$$

8. YGEQ Total government revenue

$${YG}_{r} = {MTAX}_{r} + {ETAX}_{r} + {STAX}_{r} + {ITAX}_{r} + {FYTAX}_{r} + {HTAX}_{r} + {FTAX}_{r}, r \in rgn$$

The following equations for tax rates are specified to support model closures with fixed government savings and a proportional or additive endogenous shift in user-selected tax rates to match a fixed government savings volume. Consider TEDEF for example. The parameter cube teb contains the observed export tax rates in the benchmark year. In a standard closure with flexible government savings and fixed tax rates, dabte_c,w,r_ = 0, the multiplicative tax rate shift variable TEADJ is fixed at unity and the additive tax rate shift variable is fixed at zero.

In a closure with fixed government savings and a proportional endogenous adjustment in TE, TEADJ would be flexed while maintaining dabte_c,w,r_ = 0. Non-proportional tax adjustments can be specified by choosing non-zero value for selected dabte entries. For uniform additive endogenous shifts in user-selected TE rates, the user would set the corresponding te01 entries to unity, flex DTE and fix TEADJ at unity while maintaining dabte_c,w,r_ = 0.

1. TEDEF Export tax rate

$${TE}_{c,w,r}=\left( {teb}_{c,w,r}+{dabte}_{c,w,r} \right)*{TEADJ}_{r}+{te01}_{c,w,r}*{DTE}_{r} , c\in cer$$

2. TMDEF Import tariff rate

$${TM}_{w,c,r}=\left( {tmb}_{w,c,r}+{dabtm}_{w,c,r} \right)*{TMADJ}_{r}+{tm01}_{w,c,r}*{DTM}_{r} , c\in cmr$$

3. TSDEF Sales tax rate

$${TS}_{c,r}=\left( {tsb}_{c,r}+{dabts}_{c,r} \right)*{TSADJ}_{r}+{ts01}_{c,r}*{DTS}_{r} , c\in(cd\cup cm)$$

4. TXDEF Production tax-subsidy rate

$${TX}_{a,r}=\left( {txb}_{a,r}+{dabtx}_{a,r} \right)*{TXADJ}_{r}+{tx01}_{a,r}{*DTX}_{r}$$

5. TYFDEF Factor-specific factor income tax rate

${TYF}_{f,r}=\left( {tyfb}_{f,r}+{dabtyf}_{f,r} \right)*{TYFADJ}_{r}+{tyf01}_{a,r}{*DTYF}_{r}$

6. TYHDEF Household income tax rate

$${TYH}_{h,r}=\left( {tyhb}_{h,r}+{dabtyh}_{h,r} \right)*{TYHADJ}_{r}+{tyh01}_{h,r}{*DTYH}_{r}$$

7. TFDEF Factor use tax rate

$${TF}_{f,a,r}=\left( {tfb}_{f,a,r}+{dabtf}_{f,a,r} \right)*{TFADJ}_{r}+{tf01}_{f,a,r}{*DTF}_{r}$$

8. EGEQ Total government consumption

$${EG}_{r} = \sum_{c} {QGD}_{c,r}* {PQD}_{c,r} , r \in rgn$$

9. QGDEQ Government consumption by commodity

$${QGD}_{c,r} = {qgdconst}_{c,r}* {QGDADJ}_{r} , r \in rgn$$

### Market Clearing Block

1. QEQUIL Commodity market equilibrium for Armington composite

$${QQ}_{c,r} = {QINTD}_{c,r}+\sum_{h} {QCD}_{c,h,r}+{QGD}_{c,r}+{QINVD}_{c,r}+{qds}_{c,r} , c \in\left( cd \cup cm \right), r \in rgn$$

2. FMEQUIL Factor market equilibrium

$${FS}_{f,r}= \sum_{a} {FD}_{f,a,r} , r \in rgn$$

3. FSEQ Factor supply for non-activity-specific factors

$${FS}_{f,r}={FS0}_{f,r}*\left( \frac{{WF}_{f,r}}{{WF0}_{f,r}} \right)^{{fselas}_{f,r}} , f\in\neg spfac, r\in rgn$$

4. SPECFACEQ Factor supply for activity-specific factors (natural resources)

${FD}_{f,a,r}={FD0}_{f,a,r}*\left( \frac{{WF}_{f,r}{WFDIST}_{f,a,r}}{{WF0}_{f,r}{WFDIST0}_{f,a,r}} \right)^{{fdelas}_{f,a,r}} , f\in spfac, r\in rgn$

### Macro Closure Block

1. QINVDEQ Investment demand by commodity

$${QINVD}_{c,r} = \sum_{c} {qinvdconst}_{c,r}*{IADJ}_{r} , r \in rgn$$

2. INVESTEQ Aggregate investment demand

$${INVEST}_{r} = \sum_{c} {PQD}_{c,r}*{QINVD}_{c,r} , r \in rgn$$

3. TOTSAVEQ Total Saving

$${TOTSAV}_{r} = \left( \sum_{h} \left( {YH}_{h,r}*\left( 1-{TYH}_{h,r} \right)*{SHH}_{h,r} \right) \right)+\left( \sum_{f} \left( {deprec}_{f,r}*{YF}_{f,r} \right) \right){+ KAPGOV}_{r}+\left( {KAPWOR}_{r}*{ER}_{r} \right) , r \in rgn$$

4. SHHDEF Household propensity to save

$${SHH}_{h,r}={shhb}_{h,r}*{SADJ}_{r} , r\in rgn$$

5. KAPGOVEQ Government saving

$${KAPGOV}_{r}= {YG}_{r} - {EG}_{r} , r \in rgn$$

6. KAPREGEQ Region r’s trade deficit with region w

$${KAPREG}_{w,r}= \sum_{c} \left( {PWMFOB}_{w,c,r}* {QMR}_{w,c,r} \right)- \sum_{c} \left( {PWE}_{c,w,r}*{QER}_{c,w,r} \right) , w \in wgn$$

7. KAPEQUIL Region r’s total current account deficit (Foreign saving)

$${KAPWOR}_{r}= \sum_{w} {KAPREG}_{w,r}+\sum_{rp} ({hhtrans}_{rp,r}-{hhtrans}_{r,rp})$$

8. VFDOMDEQ Nominal absorption

$${VFDOMD}_{r}= \sum_{c} {PQD}_{c,r}* \left( \left( \sum_{h} {QCD}_{c,h,r} \right)+{QGD}_{c,r}+ {QINVD}_{c,r} \right) , r \in rgn$$

9. VGDSHEQ Government share in absorption

$${VGDSH}_{r}* {VFDOMD}_{r}= \sum_{c} \left( {PQD}_{c,r}* {QGD}_{c,r} \right) , r \in rgn$$

10. INVESTSHEQ Investment share in absorption

$${INVESTSH}_{r}* {VFDOMD}_{r}= {INVEST}_{r} , r \in rgn$$

11. CPIDEF Consumer price index

$${CPI}_{r}= \sum_{c} \left( {comtotsh}_{c,r}* {PQD}_{c,r} \right) , r \in rgn$$

12. PPIDEF Producer price index

$${PPI}_{r}= \sum_{c} \left( {vddtotsh}_{c,r}* {PD}_{c,r} \right) , r \in rgn$$

13. ERPIDEF

$$ERPI= \sum_{ref} \left( {tradtotsh}_{r}* {ER}_{ref} \right)$$

### System Consistency Checks

1. COMTRADE Global margin service import value must match margin export value

$$\sum_{w} {PWM}_{w,ct2,globe}{QMR}_{w,ct2,globe}=\sum_{w} {PWE}_{ct2,w,globe}{QER}_{ct2,w,globe}+GLOBESLACK$$

2. WALRASEQ Total savings must match total investment including stock changes

$${TOTSAV}_{r}={INVEST}_{r}+\sum_{c} {PQD}_{c,r}{qds}_{c,r}+WALRAS , r\in rgn$$

3. SYSEQUIL Global sum of regional current account balances must be zero

$KAPWORSYS= \sum_{rgn} {KAPWOR}_{rgn}$ .

# Linking IMPACT to GLOBE

The following description is a summary of the coupling of IMPACT and GLOBE. More detailed documentation of this coupling can be found in Willenbockel et al. (2018) which is available in full at: <http://ebrary.ifpri.org/cdm/ref/collection/p15738coll2/id/132757>

GLOBE (McDonald et al., 2007), a global dynamic computable general equilibrium (CGE) model, is coupled with IMPACT (Robinson et al., 2015), a global partial equilibrium multi-market model, to estimate the economy-wide repercussions on aggregate households in scenarios of alternative agricultural futures. Agricultural productivity shocks simulated in IMPACT are aggregated and passed to GLOBE, which estimates knock-on effects in the broader economy that are summarized as changes in real household income and aggregated to the national level. The changes to national incomes are passed back to IMPACT to be run as an income shock effect for the given alternative agricultural scenario (Figure S2).

Figure S2 Coupling IMPACT and GLOBE to capture spillovers from agricultural productivity scenarios

First Run of IMPACT

GLOBE

Second Run of IMPACT

- GLOBE uses aggregate changes in agricultural productivity from the first run of IMPACT as Total Factor Productivity shocks
- The results of the simulation compared to calibrated GLOBE baseline to estimate changes in household incomes
- IMPACT re-simulates scenario, with change in national income aggregated from GLOBE
- IMPACT simulates changes in agricultural productivity

The agricultural sector is aligned between the two models by calibrating a baseline scenario in GLOBE that replicates IMPACT’s aggregated baseline (SSP2-NoCC) scenario. The paths for exogenous driver variables in IMPACT, including GDP and population growth, agricultural land supply, and commodity price projections, are aggregated to match GLOBE’s regions and sectors (see Table S4 and Table S5 for concordances between IMPACT and GLOBE). The calibrated baseline for GLOBE provides the reference to estimate changes caused by new scenarios from IMPACT. The comparison of two GLOBE solutions provides indications of the direction and order of magnitude of knock-on effects for non-agricultural and macroeconomic variables.

The aggregate real income effects associated with the agricultural climate change impacts and adaptation investment scenarios generated by GLOBE are downscaled to the IMPACT regions. To downscale the real income effects from aggregate GLOBE regions to IMPACT countries, we use the relationship between household real income change and change in share of food production in value-added GDP, which explains 94 percent of the variation in real income effects (Figure S3). We calculate the initial food value-added shares in GDP for all 135 regions in the fully disaggregated GTAP 8.1 database (Narayanan et al., 2012) and use these to downscale the real income effects from the 15 GLOBE regions. These results are then recalibrated to ensure the weighted average of the GTAP regions match the GLOBE regions. Finally, we map the changes in income to the IMPACT regions and run these as an income shock in the specified scenario.

Figure S3 Correlation between real income change and the share of food production value-added GDP in 2050

Note: Percent change is calculated in 2050 between the climate change scenario using HGEM, and the NoCC scenario

Once the changes in per capita GDP are introduced to IMPACT we can observe demand-side effects. Changes in average income lead to changes in demand, which will in turn lead to changes in calorie consumption and hunger. These shifts in demand will lead to price changes then feedback to the supply-side. Table S3 summarizes differences for selected variables and regions with and without GLOBE in 2030.

Table S3 Comparing results for COMP scenario in 2030 with and without connection to GLOBE

| Variable | Region | Without Globe | With Globe | % Difference |
| --- | --- | --- | --- | --- |
| Average Price ($/mt) | World | 591 | 599 | 1.37% |
| Aggregate Yield  (mt/ha) | World | 7.60 | 7.62 | 0.37% |
|  | Developing Countries | 7.62 | 7.65 | 0.40% |
|  | Africa | 5.05 | 5.06 | 0.30% |
|  | Northern Africa | 6.23 | 6.26 | 0.34% |
|  | Africa South of the Sahara | 4.65 | 4.66 | 0.29% |
|  | Western Africa | 4.49 | 4.50 | 0.30% |
|  | Eastern Africa | 4.49 | 4.50 | 0.29% |
|  | Central Africa | 5.07 | 5.08 | 0.18% |
|  | Southern Africa | 12.64 | 12.69 | 0.40% |
| Calorie Availability (kcal/person) | World | 3,133 | 3,145 | 0.37% |
|  | Developing Countries | 3,069 | 3,083 | 0.45% |
|  | Africa | 2,823 | 2,834 | 0.39% |
|  | Northern Africa | 3,284 | 3,291 | 0.20% |
|  | Africa South of the Sahara | 2,704 | 2,715 | 0.42% |
|  | Western Africa | 3,009 | 3,028 | 0.63% |
|  | Eastern Africa | 2,426 | 2,434 | 0.33% |
|  | Central Africa | 2,576 | 2,577 | 0.06% |
|  | Southern Africa | 3,162 | 3,187 | 0.81% |
| Population at Risk  (million) | World | 422.39 | 416.49 | -1.40% |
|  | Developing Countries | 409.47 | 403.16 | -1.54% |
|  | Africa | 163.31 | 161.34 | -1.20% |
|  | Northern Africa | 15.98 | 15.97 | -0.07% |
|  | Africa South of the Sahara | 157.10 | 155.14 | -1.25% |
|  | Western Africa | 22.19 | 21.83 | -1.62% |
|  | Eastern Africa | 99.39 | 97.96 | -1.44% |
|  | Central Africa | 23.06 | 23.06 | 0.00% |
|  | Southern Africa | 2.69 | 2.52 | -6.57% |
| GDP per Capita  (000 USD) | World | 17.29 | 17.60 | 1.81% |
|  | Developing Countries | 12.49 | 12.90 | 3.27% |
|  | Africa | 4.97 | 5.10 | 2.51% |
|  | Northern Africa | 9.90 | 10.20 | 3.04% |
|  | Africa South of the Sahara | 3.81 | 4.00 | 5.09% |
|  | Western Africa | 3.88 | 4.10 | 5.58% |
|  | Eastern Africa | 2.28 | 2.30 | 0.77% |
|  | Central Africa | 3.14 | 3.20 | 1.77% |
|  | Southern Africa | 16.58 | 17.30 | 4.35% |

Table S4 Concordance between GLOBE, GTAP 8.1, and IMPACT regions

| **GLOBE-15** | | **GTAP 8.1** | | **IMPACT** | |
| --- | --- | --- | --- | --- | --- |
| **Code** | **Name** | **Code** | **Name** | **Code** | **Name** |
| EAfrica | Eastern Africa | ETH | Ethiopia | ETH | Ethiopia |
|  |  | KEN | Kenya | KEN | Kenya |
|  |  | MDG | Madagascar | MDG | Madagascar |
|  |  | MOZ | Mozambique | MOZ | Mozambique |
|  |  | MWI | Malawi | MWI | Malawi |
|  |  | RWA | Rwanda | RWA | Rwanda |
|  |  | TZA | Tanzania | TZA | Tanzania |
|  |  | UGA | Uganda | UGA | Uganda |
|  |  | XAC | South Central Africa | AGO | Angola |
|  |  |  |  | COD | Democratic Republic of Congo |
|  |  | XCF | Central Africa | CAF | Central African Republic |
|  |  |  |  | COG | Congo |
|  |  | XCF | Gabon | GAB | Gabon |
|  |  | XCF | Rest of Central Africa | GNQ | Equatorial Guinea |
|  |  |  |  | TCD | Chad |
|  |  | XEC | Rest of East Africa | BDI | Burundi |
|  |  |  |  | DJI | Djibouti |
|  |  |  |  | ERI | Eritrea |
|  |  |  |  | SDN | Sudan |
|  |  |  |  | SOM | Somalia |
|  |  | ZMB | Zambia | ZMB | Zambia |
|  |  | ZWE | Zimbabwe | ZWE | Zimbabwe |
| MENA | Middle East  and North Africa | ARE | United Arab Emirates | RAP | Rest of Arab Peninsula |
|  |  | EGY | Egypt | EGY | Egypt |
|  |  | IRN | Iran | IRN | Iran |
|  |  | ISR | Israel | ISR | Israel |
|  |  | MAR | Morocco | MOR | Morocco |
|  |  | SAU | Saudi Arabia | SAU | Saudi Arabia |
|  |  | TUN | Tunisia | TUN | Tunisia |
|  |  | TUR | Turkey | TUR | Turkey |
|  |  | XNF | Rest of North Africa | DZA | Algeria |
|  |  |  |  | LBY | Libya |
|  |  | XWS | Rest of West Asia | IRQ | Iraq |
|  |  |  |  | JOR | Jordan |
|  |  |  |  | LBN | Lebanon |
|  |  |  |  | PSE | Occupied Palestinian Territory |
|  |  |  |  | SYR | Syria |
|  |  |  |  | YEM | Yemen |
| SAfrica | Southern Africa | BWA | Botswana | BWA | Botswana |
|  |  | NAM | Namibia | NAM | Namibia |
|  |  | XSC | Rest of SACU | LSO | Lesotho |
|  |  | XSC | Swaziland | SWZ | Swaziland |
|  |  | ZAF | South Africa | ZAF | South Africa |
|  |  |  |  |  |  |
|  |  |  |  |  |  |
|  |  |  |  |  |  |
|  |  |  |  |  |  |
| WAfrica | Western Africa | BEN | Benin | BEN | Benin |
|  |  | BFA | Burkina Faso | BFA | Burkina Faso |
|  |  | CIV | Ivory Coast | CIV | Ivory Coast |
|  |  | CMR | Cameroon | CMR | Cameroon |
|  |  | GHA | Ghana | GHA | Ghana |
|  |  | GIN | Guinea | GIN | Guinea |
|  |  | NGA | Nigeria | NGA | Nigeria |
|  |  | SEN | Senegal | SEN | Senegal |
|  |  | TGO | Togo | TGO | Togo |
|  |  | XWF | Rest of West Africa | GMB | Gambia |
|  |  |  |  | GNB | Guinea-Bissau |
|  |  |  |  | LBR | Liberia |
|  |  |  |  | MLI | Mali |
|  |  |  |  | MRT | Mauritania |
|  |  |  |  | NER | Niger |
|  |  |  |  | SLE | Sierra Leone |

Table S5 Concordance between GLOBE, GTAP 8.1, and IMPACT regions

| Food  Group | GTAP | | IMPACT | |
| --- | --- | --- | --- | --- |
|  | Code | Name | Code | Name |
| Animal  Products | CTL | Bovine cattle, sheep, goats, and horses | CBEEF | Cattle |
|  |  |  | CLAMB | Sheep and Goats |
|  | OAP | Animal products nec | CEGGS | Eggs |
|  |  |  | CPORK | Pigs |
|  |  |  | CPOUL | Poultry |
|  | RMK | Raw milk | CMILK | Dairy |
| Cereals | GRO | Cereal grains nec | CBARL | Barley |
|  |  |  | CMAIZ | Maize |
|  |  |  | CMILL | Millet |
|  |  |  | COCER | Other Cereals |
|  |  |  | CSORG | Sorghum |
|  | PDR | Paddy rice | CRICE | Rice |
|  | WHT | Wheat | CWHEA | Wheat |
| Fruits,  Vegetables,  Pulses,  Roots, and  Tubers | V_F | Vegetables, fruit, nuts | CBANA | Bananas |
|  |  |  | CBEAN | Beans |
|  |  |  | CCASS | Cassava |
|  |  |  | CCHKP | Chickpeas |
|  |  |  | CCOWP | Cowpeas |
|  |  |  | CLENT | Lentils |
|  |  |  | COPUL | Other Pulses |
|  |  |  | CORAT | Other Roots & Tubers |
|  |  |  | CPIGP | Pigeonpeas |
|  |  |  | CPLNT | Plantains |
|  |  |  | CPOTA | Potato |
|  |  |  | CSUBF | (Sub)-Tropical Fruits |
|  |  |  | CSWPT | Sweet Potatoes |
|  |  |  | CTEMF | Temperate Fruits |
|  |  |  | CVEGE | Vegetables |
|  |  |  | CYAMS | Yams |
| Oilseeds | OSD | Oilseeds | CGRND | Groundnuts |
|  |  |  | CPALM | Oil Palm Fruit |
|  |  |  | CRPSD | Rapeseed |
|  |  |  | CSNFL | Sunflower Seeds |
|  |  |  | CSOYB | Soybeans |
|  |  |  | CTOLS | Total Other Oilseeds |
| Vegetable  Oils | VOL | Vegetable oils and fats | CGDML | Groundnut Meal |
|  |  |  | CGDOL | Groundnut Oil |
|  |  |  | CPKML | Palm Kernel Meal |
|  |  |  | CPKOL | Palm Kernel Oil |
|  |  |  | CPLOL | Palm Oil |
|  |  |  | CRPML | Rapeseed Meal |
|  |  |  | CRPOL | Rapeseed Oil |
|  |  |  | CSBML | Soybean Meal |
|  |  |  | CSBOL | Soybean Oil |
|  |  |  | CSFML | Sunflower Meal |
|  |  |  | CSFOL | Sunflower Oil |
|  |  |  | CTOML | Total Other Oilseed Meal |
|  |  |  | CTOOL | Total Other Oils |
| Sugar | C_B | Sugar cane, sugar beet | CSUGB | Sugar beet |
|  |  |  | CSUGC | Sugarcane |
|  | SGR | Sugar | CSUGR | Sugar |
| Other | OCR | Crops nec | CCAFE | Coffee |
|  |  |  | CCOCO | Cocoa |
|  |  |  | COTHR | Other Crops |
|  |  |  | CTEAS | Tea |
|  | PFB | Plant-based fibers | CCOTT | Cotton |

# Agricultural Research and Productivity Growth

In IMPACT, improvements in agricultural productivity are represented by exogenous growth rates that embody historical trends and expert opinion of how the agriculture sector will develop over the long run. One of the primary drivers of long run productivity growth is technology development. We have applied an R&D cost estimation methodology to assess the potential investment costs required to achieve projected agricultural productivity in IMPACT scenarios.

Investments in research (R) take time to bear fruit, as new ideas can take years to develop, apply and diffuse widely. To capture these lags, our cost estimation model is based on the analytical framework used by Esposti and Pierani (2003) that relied on the perpetual inventory methodology (PIM) to calculate knowledge stocks (K) over time. Knowledge itself can decay (δ) as older technologies become obsolete or irrelevant (e.g. cold tolerant technologies in an increasingly warm world). In the long run, productivity (P) grows if the stock of knowledge increases, which can only happen if R&D investment grows at a faster rate than the rate of decay of the stock of knowledge.

Assuming a production function based exclusively on technology, where changes in total factor productivity (TFP) is driven mostly by changes in K and the TFP elasticity with respect to changes in the stock of knowledge.

|  | $P=TFP$ | (1) |
| --- | --- | --- |

We can take the log of this production function to estimate growth in productivity (g):

|  | $g=\frac{dP}{P}=\gamma\frac{dK}{K}$ | (2) |
| --- | --- | --- |

The stock of knowledge can be estimated as the stock of knowledge from the previous period (t-1) left after the decay of knowledge (δ) plus research investments (R).

|  | $K=\left( 1-\delta\right)K_{t-1}+R$ | (3) |
| --- | --- | --- |

The change in stock of knowledge can be expressed as the difference from the current stock of knowledge and the stock from the previous period:

|  | $dK=K-K_{t-1}$ | (4) |
| --- | --- | --- |

Combining equations 3 and 4 allows us to express the change in the stock of knowledge in relation to research investments and the decay of knowledge.

|  | $dK=R-\delta K$ | (5) |
| --- | --- | --- |

To solve for the research levels required to achieve a specific level of productivity growth we solve for dK in equation 2 and set it equal to equation 5, which leaves us with the following relationship between research, and productivity growth.

|  | $R=K\times\left( \delta+\frac{g}{\gamma} \right)$ | (6) |
| --- | --- | --- |

Due to data limitations we do not have detailed investment streams by crop globally, which prevents us from individually estimating investment costs by commodity. Instead, we must estimate the costs of achieving the aggregate increases in productivity across all agricultural commodities,. While we cannot at present disaggregate investment costs by crop, we are able to recognize the differences in research capacity and efficiency by region and type of public research institution. To reflect these differences, we disaggregated agricultural R&D using statistics compiled by ASTI (Beintema et al., 2012; ASTI, 2016) and using elasticities of productivity (γ) with respect to research investments from literature (Evenson and Gollin 2003; Nin-Pratt 2015; Nin-Pratt et al., 2015; and Nin-Pratt 2016). Table S6 summarizes the starting elasticities used to estimate research investment costs in IMPACT

Table S6 TFP-Knowledge Stock Elasticities

|  | CGIAR | NARS |
| --- | --- | --- |
| Africa South of the Sahara | 0.075 | 0.05 |
| Northern Africa and West Asia | 0.120 | 0.10 |
| East Asia | 0.140 | 0.14 |
| South Asia | 0.100 | 0.10 |
| Latin America and Caribbean | 0.100 | 0.10 |

Source: Authors calculation using information from Evenson and Gollin (2003).

IMPACT baseline productivity trends for the SSP 2 scenario assume that developing regions shrink global yield gaps leading to convergence of agricultural productivity (Robinson et al., 2015). To be consistent with this underlying assumption and the economic growth projected under SSP 2 we have applied a time trend on R&D elasticities, with R&D elasticities converging in developing regions towards those observed in developed regions. This reflects in a stylized fashion the role that the returns to R&D investments are in part endogenous to investment decisions (Aghion and Jaravel 2015; Baker and Shittu, 2007), and that if historic trends of increased investment continue, and if we observe future convergence, that we would also expect to see the research capacity in developing regions to increase.

Additionally, knowledge is an intangible and non-rival good that, when disseminated, can spread far and wide. To capture this behavior, we have borrowed from Alston et al. (2011) to incorporate spillover effects. We use spillover elasticities to represent differences in regional capacity to access and apply outside knowledge along with distance metrics to estimate the applicability of outside research to regional agriculture (i.e. new technologies developed for maize in Kenya are more likely to be applicable in Tanzania than to maize production in France or, even less so, to coffee production in Indonesia).

The average investment cost for the CGIAR between 2010 and 2030 is about $1.16 billion per year, starting from less than 0.6 billion per year in 2010 to almost 1.7 billion in 2030 (an average annual growth rate of 5.1 percent). This projected growth assumes that investment levels follow historical trends and generally track projected GDP growth between 2010 and 2050 (Table S7), though generally higher in the first 20 years than the latter.

Table S7 Comparing the average annual growth rate of CGIAR investments and GDP by region (percent per year)

| Region | 2010-2030 | | 2030-2050 | | 2010-2050 | |
| --- | --- | --- | --- | --- | --- | --- |
|  | CGIAR | GDP | CGIAR | GDP | CGIAR | GDP |
| Africa South of the Sahara | 6.7 | 5.6 | 1.9 | 5.2 | 4.3 | 5.4 |
| North Africa and West Asia | 2.9 | 4.1 | 1.8 | 3.2 | 2.3 | 3.7 |
| Africa and West Asia | 6.3 | 4.7 | 1.9 | 4.1 | 4.1 | 4.4 |
| Other Developing Countries | 3.2 | 5.4 | 1.4 | 2.7 | 2.3 | 4.1 |
| All Developing Countries | 5.1 | 5.3 | 1.8 | 3.0 | 3.4 | 4.1 |

Projections for NARS investments are based on historical trends (Beintema et al., 2012; ASTI, 2016) as well as literature on the returns to investments from NARS (Evenson and Gollin, 2003). Figure S4 highlights the estimated NARS R&D investments for the world, developing countries, and the two regions that we report for Africa. Globally, baseline investment costs increase from around $18 billion per year in 2010 to nearly $21 billion per year by 2050. Both African regions are projected to grow at a faster rate than the developing country average, with growth of 65 and 42 percent by 2030 for Africa South of the Sahara and North Africa and West Asia, respectively. Combined, the total investment in agricultural R&D in the baseline scenario amounts to almost $20 billion per year between 2010 and 2030 (Table S8).

Figure S4 Projected NARS research investments (billion 2005 USD)


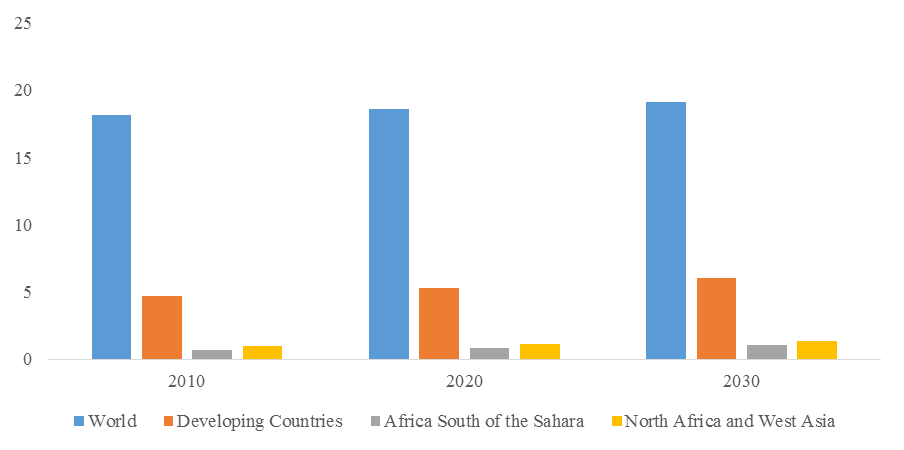


Table S8 Average annual baseline investments (2010-2030) in Agricultural R&D (billion 2005 USD)

| Region | CGIAR | NARS | Total |
| --- | --- | --- | --- |
| Africa South of the Sahara | 0.68 | 0.85 | 1.53 |
| North Africa and West Asia | 0.06 | 1.12 | 1.19 |
| Africa and West Asia | 0.74 | 1.97 | 2.72 |
| Other Developing Countries | 0.41 | 3.38 | 3.79 |
| All Developing Countries | 1.16 | 5.35 | 6.51 |
| Developed Countries | - | 13.26 | 13.26 |
| World | 1.16 | 18.61 | 19.77 |

Note: CGIAR investments are focused on the developing world

# Costing Irrigation Expansion and Improvement

Improvements in water resource management come with costs of developing and implementing new technologies and infrastructure (canals, damns, drip irrigation, etc.). We use data for estimated unit total cost for new construction in Inocencio *et al.* (2007) along with unit irrigation investment costs in FAO’s AquaStat database (FAO 2016b). The cost of the water projects in the FAO database are summarized as costs per hectare, with the cost of expansion differentiated by six developing regions that align with our reporting regions (Table S9).

Table S9 Unit cost of irrigation expansion (2005 USD per hectare) by region

| Region | Cost |
| --- | --- |
| Eastern Asia | 9,236 |
| Southern Asia | 3,812 |
| Former Soviet Union | 9,237 |
| Africa South of the Sahara | 16,226 |
| Middle East and North Africa | 9,748 |
| Latin America and Caribbean | 5,512 |

Source: Unit total cost for new construction in 2000 US dollar from Inocencio *et al.* (2007).

We assume that improvements in water use efficiency (WUE) are achieved by increased adoption of high efficiency irrigation technologies and improved management. The investment cost of WUE improvement is calculated as the increase in the share of irrigated area using high efficiency technologies multiplied by unit area cost of irrigation modernization using sprinklers as a proxy (Table S10).

Table S10 Unit cost (2005 USD per hectare) of increasing water use efficiency by region

| Region | Cost |
| --- | --- |
| Asia^1^ | 2,408 |
| Africa South of the Sahara | 4,843 |
| Middle East and North Africa | 1,071 |
| Latin America and Caribbean | 4,843 |

Note: ^1^ Asia costs were mapped to EAP, SAS, and FSU regions

Table S11 summarizes the assumed changes in irrigated area, water use, and associated investments costs between 2010 and 2030.

Table S11 Summary of Assumptions on Expansion of Irrigated Area and Water Use Efficiency by Region and Associated Costs

|  | Irrigated Area | | | | Water Use | | | |
| --- | --- | --- | --- | --- | --- | --- | --- | --- |
| Region | 2010^1^ | 2030 | Annual  Growth^2^ | Annual  Cost^3^ | 2010^4^ | 2030 | Annual  Growth | Annual  Cost |
| Africa South of the Sahara | 9 | 12 | 1.9% | 2.98 | 16,556 | 15,526 | -0.3% | 0.13 |
| North Africa and West Asia | 23 | 26 | 0.5% | 0.81 | 16,962 | 16,663 | -0.1% | 0.07 |
| Africa and West Asia | 32 | 38 | 0.9% | 3.79 | 16,854 | 16,294 | -0.2% | 0.20 |
| Other Developing Countries | 283 | 313 | 0.5% | 3.77 | 8,803 | 8,401 | -0.2% | 2.02 |
| All Developing Countries | 315 | 352 | 0.5% | 7.56 | 9,617 | 9,263 | -0.2% | 2.22 |
| Developed Countries | 36 | 39 | 0.4% | 0.56 | 11,505 | 11,061 | -0.2% | 0.17 |
| World | 351 | 391 | 0.5% | 8.12 | 9,808 | 9,446 | -0.2% | 2.39 |

Notes: ^1^ Irrigated Area in 2010 and 2030 are in millions of hectares of harvested area

^2^ Annual growth rate is the average growth rate between 2010 and 2030

^3^ Annual cost is the average annual investment in billion 2005 USD per year

^4^ Water use in 2010 and 2030 are in m^3^/hectare

Soil management technologies increase the availability of water in the soil. Baseline information on investments in soil-water management technologies is speculative and, as there are no globally differentiated cost estimates for such measures, we use the estimate developed for the Agricultural Water Management Solutions Project of $179 per hectare (AWMS 2012). Developed countries are excluded from these estimates and this cost is applied to developing countries only for a total cost of $3..62 billion per year, with regional variation detailed in Table S12.

Table S12 Soil Management Investments in the Baseline (billion 2005 USD per year)

| Region | Average Annual Investment |
| --- | --- |
| Africa South of the Sahara | 0.88 |
| North Africa and West Asia | 0.64 |
| Africa and West Asia | 1.52 |
| Other Developing Countries | 2.10 |
| All Developing Countries | 3.62 |
| Developed Countries | - |
| World | 3.62 |

Note: Currently we have only estimated baseline investments in soil management technologies for developing countries

# Infrastructure Investment Costs

Assumptions for economic growth under SSP2 includes investments in new infrastructure and maintenance of existing infrastructure. Based on previous work on estimating infrastructure investments for agriculture (Rosegrant et al., 2018), we calculate baseline investments across developing countries of $25.57 billion per year. The largest investments across developing countries ($11.36 billion per year) are projected to be in expanding and upgrading electric grids followed by investments in improving and extending road networks ($9.05 billion per year). Table S13 breaks down the baseline infrastructure costs by region and infrastructure type.

Table S13 Baseline infrastructure investments by region (billion 2005 USD per year)

| Region | Roads  (Paving and Capacity) | Rail  Capacity | Electrification | Total |
| --- | --- | --- | --- | --- |
| Africa South of the Sahara | 0.02 | 0.02 | 0.14 | 0.17 |
| North Africa and West Asia | 0.05 | 0.02 | 0.84 | 0.90 |
| Africa and West Asia | 0.07 | 0.04 | 0.98 | 1.07 |
| Other Developing Countries | 8.98 | 4.16 | 11.36 | 24.50 |
| All Developing Countries | 9.05 | 4.20 | 12.34 | 25.57 |
| Developed Countries | 5.01 | 2.32 | 19.64 | 26.92 |
| World | 14.06 | 6.52 | 31.98 | 52.49 |

Works Cited in Supplementary Materials

Aghion, P. & Jaravel, X. (2015). Knowledge spillovers, innovation and growth. *The Economic Journal,* 125: 533–583. doi: 10.1111/ecoj.12199

Alston, J.M., Andersen, M.A., James, J.S. & Pardey, P.G. (2011). The economic returns to U.S. public agricultural research. *American Journal of Agricultural Economics,* 93(5): 1257-1277. doi: 10.1093/ajae/aar044

Anriquez, G., & Daidone, S. (2011). An Extended Cross-Country Database for Agricultural Investment and Capital. FAO ESA Working Paper 11-16. Available at: <http://www.fao.org/3/a-am640e.pdf>

ASTI (Agricultural Science and Technology Indicators). (2016) *ASTI Database*. Washington, DC: International Food Policy Research Institute. Available at: <https://www.ifpri.org/program/agricultural-science-and-technology-indicators-asti>

AWMS [Agricultural Water Management Solutions]. (2012). *AgWater Solutions Watershed Database*. <http://agwaterdb.iwmi.org/login.php>.

Baker E., & Shittu, E. (2007). Uncertainty and endogenous technical change in climate policy models. *Energy Economics,* 30: 2817–2828. doi: 10.1016/j.eneco.2001.10.001

Beintema, N., Stads, G., Fuglie, K., & Heisey P. (2012). *ASTI Global Assessment of Agricultural R&D Spending*. Washington, DC: International Food Policy Research Institute. doi: 10.2499/9780896298026

Boumellassa, H., Laborde, D., & Mitaritonna, C. (2009). A Picture of Tariff Protection across the World in 2004: MAcMap-HS6, Version 2. IFPRI Discussion Paper 903. Washington, DC: International Food Policy Research Institute. [www.ifpri.org/sites/default/files/publications/ifpridp00903.pdf](http://www.ifpri.org/sites/default/files/publications/ifpridp00903.pdf).

Crego, A., Larson, D., Butzer, R., & Mundlak, Y. (1998). "A New Database on Investment and Capital for Agriculture and Manufacturing." World Bank Policy Research Working Paper 2013.

Esposti, M., & Pierani, F. (2003). Building the Knowledge Stock: Lags, Depreciation, and Uncertainty in R&D Investment and Link with Productivity Growth. *Journal of Productivity Analysis,* 19(1): 33–58. doi: 10.1023/A:102181801

Dissou, Y., Karnizova, L., & Sun, Q. (2015) Industry-level Econometric Estimates of Energy-Capital-Labor Substitution with a Nested CES Production Function. *Atlantic Economic Journal,* 43(1), 107-21.

Evenson, R.E., & Gollin, D. (2003). Assessing the impact of the Green Revolution, 1960 to 2000. *Science*, 300(5620): 758–762. doi: 10.1126/science.1078710

FAO. (2016). *FAOSTAT. Online Statistical Database*. Rome: United Nations Food and Agriculture Organization. Available at: <http://faostat.fao.org/>

GAMS (General Algebraic Modeling System). (2017). *General Algebraic Modeling System (GAMS)*. Washington, DC. Available at: [www.gams.com](http://www.gams.com).

Hoogenboom, G., Jones, J.W., Wilkens, P.W., Porter, C.H., Boote, K.J., Hunt, L.A.,…Koo, J. (2012). *Decision Support System for Agrotechnology Transfer (DSSAT) Version 4.5* [CD-ROM]. Honolulu: University of Hawaii, Honolulu.

IFPRI & CGIAR. (2016). *Public Spending on Ag R&D. Online database.* Retrieved June 2016 at: <https://www.asti.cgiar.org/data/>

Inocencio, A., Kikuchi, M., Tonosaki, M., Maruyama, A., Merrey, D., Sally, H., & de Jong, I. (2007). *Cost of performance of irrigation projects: a comparison of Sub-Saharan Africa and other developing regions*. Colombo, Sri Lanka: International Water Management Institute. doi: 10.3910/2009.109

International Trade Center. (2006). *User Guide—Market Access Map: Making Tariffs and Market Access Barriers Transparent*. Geneva: Market Analysis Section, Division of Product and Market Development, International Trade Center. Available at: [www.macmap.org/User Guides/MAcMap-userguide-EN.pdf](http://www.macmap.org/User%20Guides/MAcMap-userguide-EN.pdf) ss

Jones, J.W., Hoogenboom, G., Porter, C.H., Boote, K.J., Batchelor, W.D., Hunt, L.A., … Ritchie, J.T. (2003). DSSAT Cropping System Model. *European Journal of Agronomy* 18:235–265. doi: 10.1016/S1161-0301(02)00107-7

Koesler, S., & Schymura, M. (2015) Substitution Elasticities in a Constant Elasticity of Substitution Framework – Empirical Estimates Using Nonlinear Least Squares. *Economic Systems Research* 27(1), 101-121.

McDonald, S., Thierfelder, K., & Robinson, S. (2007). Globe: A SAM Based Global CGE Model using GTAP Data. USNA Working Paper 14. US Naval Academy, Annapolis.

Narayanan, G.B. & Walmsley, T.L. (Eds.) (2008)*. Global Trade, Assistance, and Production: The GTAP 7 Data Base*. West Lafayette, Indiana, USA: Center for Global Trade Analysis, Purdue University.

Narayanan, B., Aguiar, A., & McDougall, R. (Eds) (2012). *Global Trade, Assistance, and Production: The GTAP 8 Data Base.* West Lafayette, Indiana, USA: Center for Global Trade Analysis, Purdue University.

Nin-Pratt, A. (2015). Inputs, productivity, and agricultural growth in Africa South of the Sahara. IFPRI Discussion Paper 1432. Washington, D.C.: International Food Policy Research Institute (IFPRI). <http://ebrary.ifpri.org/cdm/ref/collection/p15738coll2/id/129095>

–––––––. (2016). "Inputs, Productivity and Agricultural Growth in Sub-Saharan Africa." Chapter 11 in Greene, W.H., Khalaf L., Sickles R.C., Veall M., & Voia, M.C. (eds.) *Productivity and Efficiency Analysis*. Springer.

Nin-Pratt, A., Falconi, C., Ludena, C. & Martel, P.. (2015). Productivity and the performance of agriculture in Latin America and the Caribbean: From the lost decade to the commodity boom. IDB Working Paper Series 608. Inter-American Development Bank (IDB). <http://cdm15738.contentdm.oclc.org/cdm/ref/collection/p15738coll5/id/5105>

OECD (Organisation for Economic Co-operation and Development). (2010). *Agricultural Market Access Data Base*. Accessed 11/1/2013 at: [www.oecd.org/site/amad](http://www.oecd.org/site/amad)

———. 2014. *Agricultural Policy Monitoring and Evaluation 2014*: OECD Countries. Paris. <http://dx.doi.org/10.1787/agr_pol-2014-en>.

Robinson, S., Mason-D’Croz, D., Islam, S., Sulser, T.B., Robertson, R., Zhu, T., …Rosegrant, M.W. (2015). *The International Model for Policy Analysis of Agricultural Commodities and Trade (IMPACT): Model Description, Version 3*. IFPRI Discussion Paper 1483. Washington, DC: IFPRI. <http://ebrary.ifpri.org/cdm/ref/collection/p15738coll2/id/129825>

Rosegrant, M., Magalhaes, E., Valmonte-Santos, R., & Mason-D’Croz, D. (2018). Returns to Investment in Reducing Postharvest Food Losses and Increasing Agricultural Productivity Growth. In B. Lomborg (Ed.), *Prioritizing Development: A Cost Benefit Analysis of the United Nations' Sustainable Development Goals* (pp. 322-338). Cambridge: Cambridge University Press. DOI: [10.1017/9781108233767.020](https://doi.org/10.1017/9781108233767.020)

Willenbockel, D., Robertson, R. D., Mason-D’Croz, D., Rosegrant, M. W., Sulser, T., Dunston, S., & Cenacchi, N. (2018). *Dynamic computable general equilibrium simulations in support of quantitative foresight modeling to inform the CGIAR research portfolio: Linking the IMPACT and GLOBE models.* (IFPRI Discussion Papers No. 1738). Washington D.C. Retrieved from <http://ebrary.ifpri.org/cdm/ref/collection/p15738coll2/id/132757>

United Nations, European Commission, International Monetary Fund, Organisation for Economic Cooperation and Development and World Bank (1993): System of National Accounts 1993. Brussels/Luxembourg, New York, Paris, Washington, D.C. Available at: <http://unstats.un.org/unsd/nationalaccount/docs/1993sna.pdf>

–––––––. (2009): System of National Accounts 2008. New York. Available at: <http://unstats.un.org/unsd/nationalaccount/docs/SNA2008.pdf>

United Nations Statistics Division, (2016). International Standard Industrial Classification of All Economic Activities, Rev.3. Available at: <http://unstats.un.org/unsd/cr/registry/regcst.asp?Cl=2>

1. In IMPACT, area is treated as harvested area, which is the total area planted and harvested within a year, and may include multi-cropping or multiple harvests and differ from total arable land or reported physical area. [↑](#footnote-ref-1)
2. FPUs are sub-national spatial units that represent the intersection of 154 water basins (e.g. Amazon, Nile) and the 158 national boundaries. [↑](#footnote-ref-2)
3. Crops and livestock currently do not include intermediate inputs in the net price equation and instead directly take input price effects through supply elasticities in the crop yield and animal number equations. [↑](#footnote-ref-3)
4. Note stocks are constant and exogenous. [↑](#footnote-ref-4)
5. These elasticities are customarily labelled (KL)E elasticities in the empirical literature. Van der Werf (2008) reports industry-level estimates ranging from of 0.17 to 0.64 using data for 12 OECD countries. Dissou et al. (2015) report corresponding estimates of 0.1 to 0.48 for Canada. Using data for the 40 countries covered by the new World Input-Output Database WIOD), Koesler and Schymura (2015) find (KL)E elasticity values in a range from 0.04 to 1.07. [↑](#footnote-ref-5)
